# Organometallic Half-Sandwich Complexes of 1,10-Phenanthroline Derivatives with Improved Solubility, Albumin-Binding, and Nanoformulation Potential Targeting Drug Resistance in Cancer

**DOI:** 10.1021/acs.inorgchem.5c01556

**Published:** 2025-07-11

**Authors:** Egon F. Várkonyi, Szilárd Tóth, Tamás Pivarcsik, Orsolya Dömötör, Ottó Berkesi, Nóra V. May, Gergely Szakács, Edit Csapó, Éva A. Enyedy

**Affiliations:** † Department of Molecular and Analytical Chemistry, Interdisciplinary Excellence Centre, 37442 University of Szeged, Dóm tér 7-8, H-6720 Szeged, Hungary; ‡ MTA-SZTE Lendület “Momentum” Noble Metal Nanostructures Research Group, University of Szeged, Rerrich B. Sqr. 1, H-6720 Szeged, Hungary; § Department of Physical Chemistry and Materials Science, Interdisciplinary Excellence Centre, University of Szeged, Rerrich B. Sqr. 1, H-6720 Szeged, Hungary; ∥ Drug Resistance Research Group, Institute of Molecular Life Sciences, 280964 HUN-REN Research Centre for Natural Sciences, Magyar Tudósok krt. 2, H-1117 Budapest, Hungary; ⊥ National Laboratory for Drug Research and Development, Magyar Tudósok krt. 2, H-1117 Budapest, Hungary; # Centre for Structural Science, HUN-REN Research Centre for Natural Sciences, Magyar Tudósok krt. 2, H-1117 Budapest, Hungary; ¶ Center for Cancer Research, 27271 Medical University of Vienna, Borschkegasse 8a, A-1090 Vienna, Austria

## Abstract

The development of Rh­(III)­(η^5^-C_5_Me_5_) and Ru­(II)­(η^6^-*p*-cymene) complexes of 4,7-dichloro-1,10-phenanthroline (DCP) and bathophenanthroline (BP) aims to increase aqueous solubility and potential bioavailability of the lipophilic ligands while also enabling selective activity against multidrug-resistant (MDR) cancer cells. Complexes [M­(η^6^-arene/η^5^-arenyl)­(DCP/BP)­Cl]Cl were prepared and characterized by means of nuclear magnetic resonance, infrared, electrospray ionization mass spectrometry, and single crystal X-ray diffraction for [Rh­(III)­(η^5^-C_5_Me_5_)­(DCP)­Cl]­PF_6_ and [Ru­(II)­(η^6^-*p*-cymene)­(BP)­Cl]­PF_6_. The complexes are highly stable in a wide pH range with increased hydrophilicity, and the Rh complexes showed fast and significant binding to human serum albumin (HSA). Cytotoxicity tests were conducted in various breast cancer cells and in cocultured cell lines of the uterine sarcoma parental MES–SA and its MDR counterparts. Both the ligands and their organorhodium complexes displayed a higher cytotoxicity against the MDR MES-SA/Dx5 cells than against the parental cells. As the complex [Rh­(III)­(η^5^-C_5_Me_5_)­(BP)­Cl]Cl showed the most promising results (IC_50_ = 0.23 μM (MES-SA/Dx5) with selectivity ratio 6.7), it was selected for nanoformulation using HSA and also combined with d-α-tocopheryl polyethylene glycol 1000 succinate and poly­(lactic-*co*-glycolic acid). Both composites showed a good encapsulation efficiency and colloidal stability. Based on the in vitro cytotoxicity assays, the use of HSA as a carrier is a promising strategy to enhance the pharmacological properties of the MDR-selective Rh­(III)­(η^5^-C_5_Me_5_) complexes of 1,10-phenanthroline derivatives.

## Graphical abstract

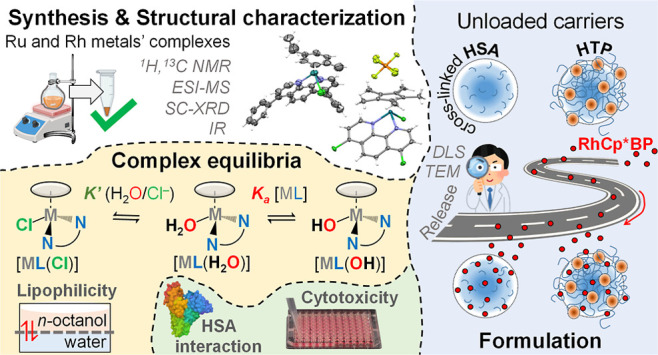

## Introduction

Cancer arises from uncontrolled cell proliferation, leading to tumor formation due to dysregulated cell division and apoptosis. Despite advances in targeted and immunotherapies, conventional chemotherapeutics, usually administered in combination therapies, remain essential in cancer treatment.^1,2^ However, the administration of chemotherapeutic drugs is frequently associated with toxic side effects and the emergence of multidrug resistance (MDR), which is often linked to the increased expression of P-glycoprotein (P-gp or ABCB1). P-gp mediates resistance by the energy-dependent efflux of a broad range of anticancer drugs, providing rescue for cancer cells.^3^ Certain compound families have been shown to selectively target and kill MDR cells.^3^ A common feature among these so-called MDR-selective compounds is the presence of a metal-chelating group.^2,4^ Certain derivatives of isatin-β-thiosemicarbazone, 1,10-phenanthroline (PHEN), and 8-hydroxyquinoline, bearing (O,N,S), (N,N) and (N,O) donor sets, respectively, have been identified as selectively toxic to MDR cells.^2,4–6^ However, solubility issues pose a significant challenge to their in vivo application. Complex formation with proper transition metals of poorly soluble chelators can enhance aqueous solubility and thus bioavailability. Notably, organometallic half-sandwich complexes of ligands from this compound groups with limited aqueous solubility such as 7-(1-piperidinylmethyl) quinolin-8-ol, 5-chloro-7-(pyrrolidin-1-ylmethyl)­quinolin-8-ol and 5-chloro-7-(piperidin-1-ylmethyl)­quinolin-8-ol^7,8^ have been shown to successfully overcome these solubility limitations. In addition, complex formation can lead to altered pharmacokinetics, often as a consequence of stronger binding to human serum albumin (HSA),^8–11^ altered drug distribution, and mechanism of action. Binding to HSA can promote tumor accumulation via the enhanced permeability and retention (EPR) effect.^12,13^

The half-sandwich Rh­(III)­(η^5^-C_5_Me_5_) complex of PHEN showed high stability in solution^14^ and exhibited significant toxicity against the human uterine sarcoma MDR cancer cell line MES-SA/Dx5 (IC_50_ = 2 μM), with a selectivity ratio (SR) of 4, which was dependent on functional P-gp. While the Ir­(III)­(η^5^-C_5_Me_5_) complex of PHEN was reported by Sadler et al. to be inactive (IC_50_ > 100 μM in A2780 cancer cells), the introduction of phenyl or biphenyl substituents on the cyclopentadienyl ring significantly increased cytotoxicity, likely due to enhanced hydrophobicity and resulting higher intracellular accumulation.^15–22^ The use of polypyridyl ligands also enhanced cytotoxic activity.^17,20^ The Ir­(III)­(η^5^-C_5_Me_5_) complex of 4,7-diphenyl derivative of PHEN (bathophenanthroline, BP) displayed significant activity against HeLa (IC_50_ = 1.1 μM) and A2780 (IC_50_ = 0.21 μM) cells, and the related tetramethyl–phenyl–cyclopentadienyl and tetramethyl–biphenyl–cyclopentadienyl complexes could efficiently overcome Pt resistance and presented excellent cancer cell selectivity.^23,24^ Although the latter complex encapsulated in the FDA-approved DSPE-PEG2000-biotin polymer demonstrated strong in vivo anticancer activity, the polymer was found unsuitable for improving its pharmacokinetic and pharmacodynamic properties.^23,24^ The Ru­(II)/Os­(II)­(η^6^-*p*-cymene) complexes of the BP, bearing dichloroacetate as a coligand, were also reported to possess improved cytotoxic activity compared to cisplatin,^19^ and submicromolar activity against highly invasive triple-negative breast cancer cells.^20^ Cu­(II)-dipeptide-BP ternary complexes were reported to be cytotoxic with micromolar/submicromolar activity against triple-negative breast cancer cells as well.^25^ Also PHEN, neocuproine, and BP are often found in the well-known Casiopeinas mixed-ligand Cu­(II) complexes, which shows significant anticancer properties.^26–28^

The objective of this study is to investigate the potential anticancer activity and MDR-selectivity of two disubstituted PHEN derivatives (4,7-dichloro-1,10-phenanthroline (DCP) and BP). As these compounds exhibit poor aqueous solubility, their corresponding Rh­(III)­(η^5^-C_5_Me_5_) (RhCp*) and Ru­(II)­(η^6^-*p*-cymene) (RuCym) complexes were synthesized and thoroughly characterized to increase hydrophilicity. Complex formation can also increase the binding strength toward HSA, which was also studied. The cytotoxicity of compounds was assessed against breast cancer lines and in a coculture of parental and MDR cell lines of uterine sarcoma. Based on cytotoxicity and MDR-selectivity, [RhCp*­(BP)­Cl]­Cl was chosen for further nanoformulation studies using HSA, as a biocompatible protein-based carrier, and HSA combined with d-α-tocopheryl polyethylene glycol 1000 succinate (TPGS), which itself possesses anticancer effect,^29,30^ and poly­(lactic-*co*-glycolic acid) (PLGA), with the aim of improving tumor-specific delivery.

## Results and Discussion

### Synthesis and Characterization of Complexes

For the synthesis, CH_2_Cl_2_ was employed as a solvent, in which the [Ru­(η^6^-*p*-cymene)­Cl_2_]_2_ or [Rh­(η^5^-C_5_Me_5_)­Cl_2_]_2_ dimeric precursor and the ligand (DCP or BP) were dissolved in a 1:2 ratio. Following 24 h of stirring, the solvent was partly evaporated, and a subsequent small portion of diethyl ether was added to facilitate precipitation. The resulting liquid, containing the solid complex, was subjected to vacuum filtration, and the solid material was collected and then dried in an oven for 4 h at 45 °C to obtain the final product. The same procedure was applied to obtain each complex. The four complexes ([RuCym­(BP)­Cl]­Cl, [RuCym­(DCP)­Cl]­Cl, [RhCp*­(BP)­Cl]­Cl, [RhCp*­(DCP)­Cl]­Cl) (Figure 1) were successfully synthesized in the form of chlorido complexes with chloride counterions. The structure and purity of the synthesized complexes were confirmed using ^1^H, ^13^C nuclear magnetic resonance (NMR) and infrared (IR) spectroscopy, in addition to electrospray ionization–mass spectrometry (ESI–MS) (see the Experimental Section). In addition, single crystals suitable for X-ray crystallographic analyses were also grown for the two complexes. Based on the characterization results, the synthesized half-sandwich complexes are pure, free of precursor and uncoordinated bidentate ligand, and their compositions and structures correspond to those shown in Figure 1.

**Figure 1:**
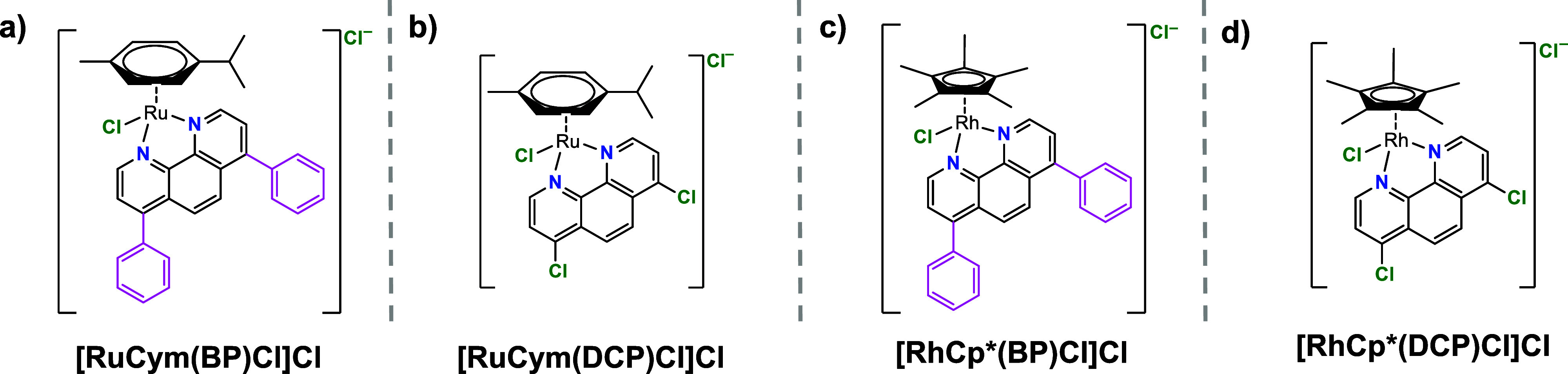
Structures of metal–ligand complexes. (a) [RuCym­(BP)­Cl]­Cl, (b) [RuCym­(DCP)­Cl]­Cl, (c) [RhCp*­(BP)­Cl]­Cl, (d) [RhCp*­(DCP)­Cl]­Cl.

### Infrared Spectroscopic Studies of the Title Complexes

The isolated complexes were studied by IR spectroscopy to confirm the coordination modes; and spectra of the metal precursors and the ligands were also recorded for comparison. The most diagnostic changes were observed in the region of the in-plane aromatic C–C stretching vibrations (1700–1350 cm^–1^), which exhibited clear shifts upon complex formation (Figure S1). These bands were particularly sensitive to coordination and reflected alterations in the electronic environment of the PHEN core. In the case of both BP and DCP complexes, selected aromatic stretching modes shifted to higher wavenumbers compared to those of the free ligands, suggesting an increased electron delocalization and redistribution of π-electron density upon metal coordination.

The C–Cl stretching vibrations, characteristic of the DCP ligand, also shifted to higher wavenumbers in the [RhCp*­(DCP)­Cl]Cl and [RuCym­(DCP)­Cl]­Cl complexes, which correlates with the electron-withdrawing effect of the metal center on the aromatic ring. Additionally, bands observed in the 875–650 cm^–1^ region (Figure S1), assigned to out-of-plane deformations of the aromatic rings, were shifted to lower wavenumbers upon complexation, consistent with changes primarily involving the σ-framework. Far-IR spectra provided further confirmation of successful complex formation, with characteristic M–Cl stretching bands observed for the mononuclear species and bridging chlorido modes detected for the dimeric precursors.

Altogether, these IR spectral features support the presence of coordinated ligands and metal-chloride bonds, in agreement with the proposed structures. A detailed vibrational assignment, supported by π-electron density calculations and further spectral interpretation, using literature data for comparison,^31–37^ is provided in the Supporting Information (Figures S1–S7).

### Structural Studies of Complexes by Single Crystal X-ray Diffraction

Single crystal X-ray diffraction (SC-XRD) analyses were conducted on complexes [RuCym­(BP)­Cl]­PF_6_ × Et_2_O (**I)** and [RhCp*­(DCP)­Cl)]­PF_6_ (**II)** to investigate their structural properties and coordination environments. To facilitate crystal growth, the chloride counterion was replaced with the bulky PF_6_^–^ ions. Both compounds were crystallized by the vapor diffusion method from either acetone or CH_2_Cl_2_ solutions. Crystal data and structure refinement for crystals **I** and **II** were collected and are presented in Table S1, their ORTEP representation are shown in Figure 2, and selected bond distances and angles of the coordination spheres of the complexes are collected in Tables S2 and S3.

**Figure 2:**
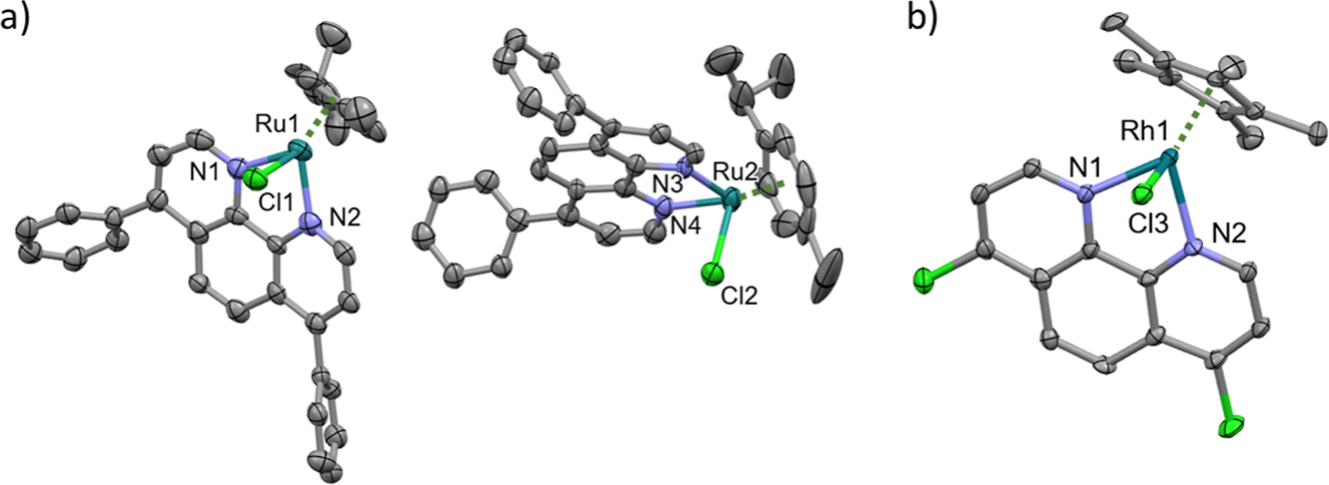
ORTEP representation of the asymmetric unit of (a) [RuCym­(BP)­Cl]­PF_6_ × Et_2_O (**I**) containing two complex molecules, and (b) [RhCp*­(DCP)­Cl]­PF_6_ (**II**), and the atom numbering of the coordinated atoms. The ellipsoids are shown at a 30% probability level. Hydrogen atoms, solvents, and counterions are omitted for clarity.

The [RuCym­(BP)­Cl]­PF_6_ × Et_2_O (**I**) complex crystallizes in the triclinic crystal system with space group *P*1̅, featuring a unit cell volume of 3734.8 Å^3^. The asymmetric unit contains two complexes with slightly different conformations. The two phenyl rings of the BP ligand are not coplanar with the PHEN ring but form significant angles of 55.8° and 68.7° in the Ru1 complex and 55.3° and 64.1° in the Ru2 complex. The Ru center is coordinated with N and Cl atoms as well as carbons from the *p*-cymene ligand. Significant bond distances, such as Ru–N (2.070–2.111 Å) and Ru–Cl (2.382–2.388 Å), indicate a robust metal–ligand framework. Intermolecular interactions, including π···π stacking and P–F···H interactions (Figure S8), contribute to a stable crystal packing with voids accounting for 5.6% of the unit cell volume (Figure S9), suggesting potential implications for solid-state properties.

The crystal structure of [RuCym­(BP)­Cl]­PF_6_ × Et_2_O was published by Colina-Vegas et al.^38^ and refined in the *C*2/*c* space group with unit cell values *a* = 31.2442(17), *b* = 15.0663(8), *c* = 22.350(2), α = γ = 90, β = 133.000(2) (ref. code FUPHAC). It revealed the disordered structures of two conformations of the Ru­(II) complexes in a 77:23 ratio (Figure S10b). A detailed comparison of this crystal structure with crystal **I** reveals that the two forms are polymorphs, and it is likely that they can interconvert as a function of temperature. (Further details of the comparison can be found in the legend of Figure S10, while the packing of the molecules and the comparison of the unit cells are shown in Figure S11.)

[RhCp*­(DCP)­Cl]­PF_6_ (**II**) exhibits an orthorhombic crystal structure with space group *Pbca* and a unit cell volume of 4823.12 Å^3^. The Rh center forms stable coordination bonds with N, Cl, and C atoms, including Rh–N (2.1230 Å) and Rh–Cl (2.3849 Å), reinforcing structural stability. The packing arrangement is stabilized by hydrogen bonding and π···π interactions (Figure S12), crucial for maintaining lattice integrity. The crystal structure of the [RhCp*­(DCP)­Cl]­PF_6_ complex was published in 2023 by Graf et al. (ref. code CIXROU).^39^ The measurement was performed at a significantly higher temperature (173 K) compared to our case, where the measurement temperature was 113 K. This temperature difference is most evident in the cell volume, which is significantly higher at a higher temperature (4875.4 Å^3^) than at a lower temperature (4823.1 Å^3^). The conformation of [RhCp*­(DCP)­Cl]^+^ is the same (Figure 3b), but small differences can be identified in the bond distances (see Table S4). Interestingly, the Rh–Cp* ring distance (M–Cg) measured by Mercury software^40^ was found to be slightly shorter in the higher measurement temperature (1.785 Å) than in the lower temperature (1.791 Å). Using the SC-XRD data obtained for the [RhCp*­(DCP)­Cl]­PF_6_ complex (Rh–centroid distance: 1.791 Å, N1–Rh–N2 angle: 77.34°, methyl group-ring plane torsion angle: 3.94°) its log*K*′ (H_2_O/Cl^–^) constant can be estimated, based on our correlation established previously.^41^ The predicted log*K*′ (H_2_O/Cl^–^) is 2.89, which show a good agreement with value (2.85) determined experimentally (vide infra).

**Figure 3:**
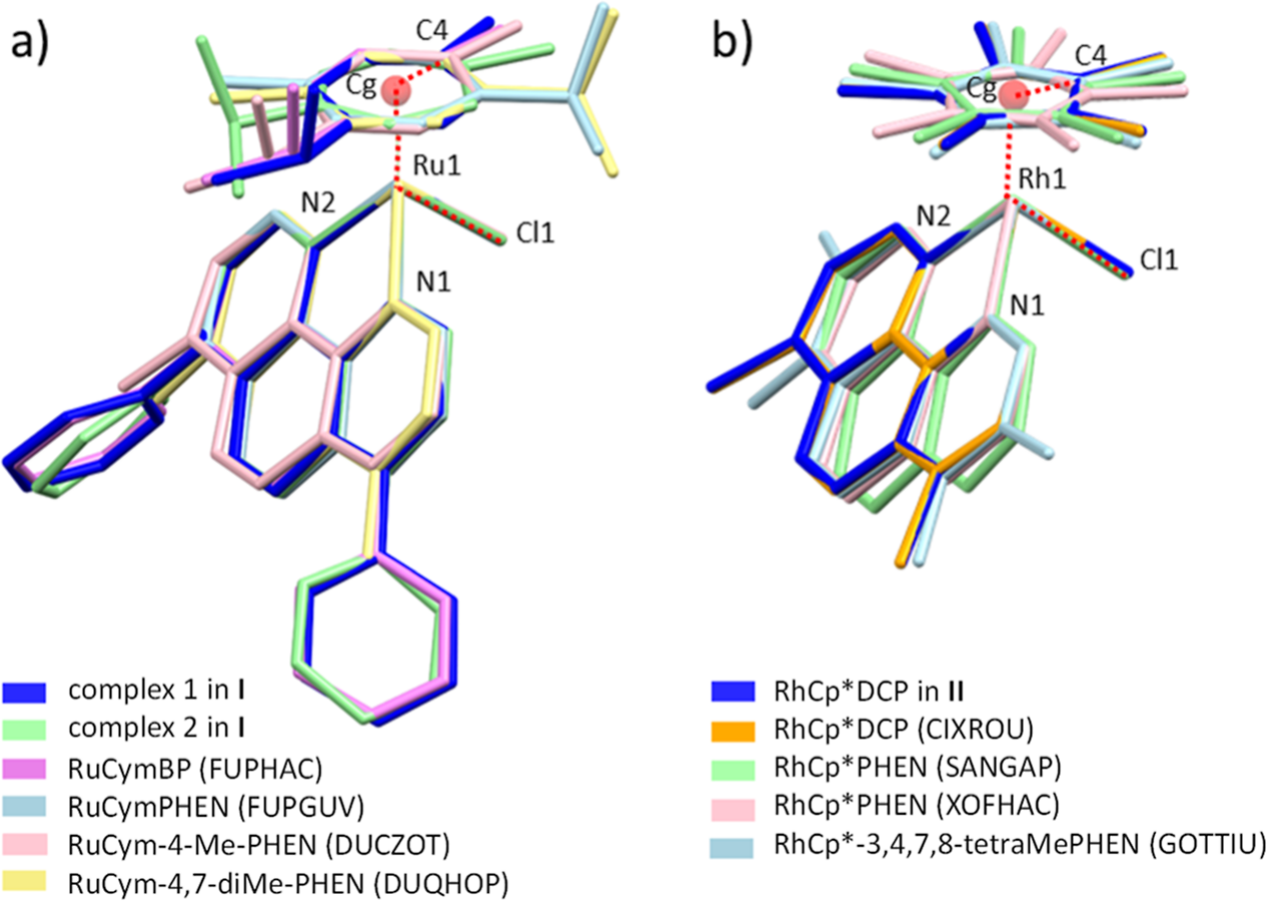
Overlaid structures of (a) [RuCym­(BP)­Cl]^+^ complexes (in crystal **I**) with related 4,7-substituted PHEN derivatives and (b) [RhCp*­(DCP)­Cl]^+^ (in crystal **II**) with related PHEN complexes found in the CSD. Atom numbering used in Table S4 and the torsion angle of C4-Cg–Ru1-Cl1 with red scattered lines are indicated.

In order to examine the variety of Ru and Rh piano-stool structures, a conformational comparison of [RuCym­(BP)­Cl]^+^ complexes (molecule 1 and 2) was performed with relevant crystal structures of [RuCym­(PHEN)­Cl]^+^ (ref. code FUPGUV^38^), [RuCym­(4-methyl-PHEN)­Cl]^+^ (ref. code DUCZOT^42^) and [RuCym­(4,7-PHEN)­Cl]^+^ (ref. code DUQHOP^43^) obtained in the Cambridge Structural Database (CSD) (20.08.2024). Overlaid structures, based on Ru1, N1, N2, and Cl1 atoms, are shown in Figure 3a. Selected bond distances and angles of the coordination sphere are collected in Table S4. The ruthenium and *p*-cymene ring distance (Ru1-Cg) as well as the Ru1–N1, –N2 and –Cl distances are similar to each other. The largest difference between the conformations is observed in the position of the *p*-cymene ring, which is influenced by the steric relations and the orientation of hydrogen bonds of the methyl or *iso*-propyl groups with neighboring molecules in each crystal structure. The methyl group on the *p*-cymene ring is positioned above the Cl^–^ ion in molecules 1 and 2 in crystal **I** and in the cases of [RuCym­(BP)­Cl]^+^ (FUPHAC) and [RuCym­(4-methyl-PHEN)­(Cl)]^+^ (DUCZOT). In contrast, it is on the opposite side from the Cl^–^ ion in [RuCym­(PHEN)­Cl]^+^ (FUPGUV) and [RuCym­(4,7-dimethyl-PHEN)­Cl]^+^ (DUQHOP) complexes. These differences are compared by the means of the C4-Cg–Ru1-Cl torsion angle. There are also significant differences in the values of the angles enclosed by the plane of the *p*-cymene and PHEN rings (see ring–ring angle in Table S4), with the smallest measured value being 47.6° for [RuCym­(4-methyl-PHEN)­Cl]^+^ and the largest value being 58.4° for [RuCym­(PHEN)­Cl]^+^.

The crystal structure of complex [RhCp*­(DCP)­Cl]­PF_6_ (**II**) was compared with relevant structures of [RhCp*­(PHEN)­Cl]­ClO_4_ (ref. code SANGAP^44^), [RhCp*­(PHEN)­Cl]­CF_3_SO_3_ (ref. code XOFHAC^45^), and [RhCp*­(3,4,7,8-tetramethyl-PHEN)­Cl]^+^ (GOTTIU^46^) (Figure 3b). It can be seen that there is also very little variation in the bond length and bond angle values for these complexes. Significant differences are found in the C4-Cg–Rh1-Cl1 torsion angle, which varies between +10.6° and −19.4°, showing the higher degree of freedom of the Cp* ring in these structures. Also, the ring–ring angle between the Cp* and PHEN rings varies significantly between 48.1° and 62.4°. It is interesting to compare some conformational data of [RuCym­(L)­Cl]^+^ with [RhCp*­(L)­Cl]^+^ complexes. For example, the average M-Cg, M-N1 and M-N2 distances of 1.680(6), 2.09(1), and 2.09(1) Å, respectively, are shorter in RuCym complexes than in RhCp* complexes, where these values are 1.783(6), 2.11(1), and 2.12(1) Å. The closely related [IrCp*­(BP)­Cl]^+^ complex and its analogues also show structures very similar to those of these RhCp* complexes (Figure S13 and Table S4).^24,47–49^

### Solution Speciation Studies of the Complexes

When [M­(η^6^-arene/η^5^-arenyl)­(ligand)­Cl]Cl complexes are dissolved in water, they first dissociate into their ions, [M­(η^6^-arene/η^5^-arenyl)­(ligand)­Cl]^+^ and Cl^–^, followed by various equilibrium processes. The chlorido coligand can be replaced by water (aquation), which may subsequently undergo deprotonation as pH increases. In addition, the dissociation of both the arene/arenyl and the bidentate ligands might be also possible; however, based on our previous findings on PHEN complexes,^14^ the release of these ligands is unlikely.

As a first step, time-dependent stability measurements were conducted by UV–visible (UV–vis) for all the metal complexes (Figure S13), focusing on their stability in a modified phosphate buffer saline (PBS’) medium to mimic conditions relevant to intravenous applications regarding the pH (7.4) and the chloride ion concentration (100 mM). Our results demonstrated no significant spectral changes over the 24 h observation period, confirming the stability of the complexes under these conditions, with the coordination bond between the metal ion and the arene/arenyl ring as well as the bidentate ligand remaining intact. ^1^H NMR spectra recorded under the same conditions also showed no changes over 24 h (see spectra of RhCp*BP in Figure S15). This high stability suggests that they are suitable for applications where prolonged systemic exposure is required. Unfortunately, determining the equilibrium constants for direct complex formation was not possible due to the poor solubility of the ligands in water. However, the formation constants determined previously for organoruthenium and organorhodium half-sandwich complexes of the reference ligand PHEN and other related oligopyridine ligands^14,50^ indicate that complexes of PHEN-type ligands exhibit very high stability, with negligible dissociation of the coordination bond between the bidentate (N,N) ligand and the metal ion even in micromolar solutions over a wide pH range. The robust stability of the analogous IrCp* complexes was also reported.^24^

The coordinated chlorido ligand can undergo spontaneous partial exchange upon dissolution, making it essential to determine the equilibrium constant for the [M­(η^6^-arene/η^5^-arenyl)­(ligand)­Cl]^+^ + H_2_O ⇌ [M­(η^6^-arene/η^5^-arenyl)­(ligand)­(H_2_O)]^2+^ + Cl^–^ process (or for the reverse direction) to evaluate the ratio of chlorido to aqua complexes. The log*K*′ (H_2_O/Cl^–^) constants were determined by UV–vis spectrophotometry (Figure 4) due to the changes in the charge transfer bands. Measurements were conducted at pH = 6.0, to characterize the chlorido/water exchange process under conditions where deprotonation of the coordinated aqua ligand does not occur, avoiding side reactions and obtaining comparable data. Importantly, this process is slow for the RuCym complexes, which requires a longer time to reach equilibrium, necessitating careful equilibration in experimental setups before further analysis. Therefore, in the case of the RuCym complexes, individual samples were prepared and their spectra were measured after 1 h, while the spectra for the RhCp* complexes were obtained by direct titration with the KCl solution. The appearance of isosbestic points in the spectra confirms the clear equilibrium between chlorido- and aqua complexes. For RuCym complexes, the log*K*′ (H_2_O/Cl^–^) values were significantly lower than those of the RhCp* complexes, indicating a stronger tendency of the RhCp* complexes to retain the chlorido ligand in the coordination sphere, as it was found for the complexes of PHEN or neocuproine^14^ as well (Table 1).

**Figure 4:**
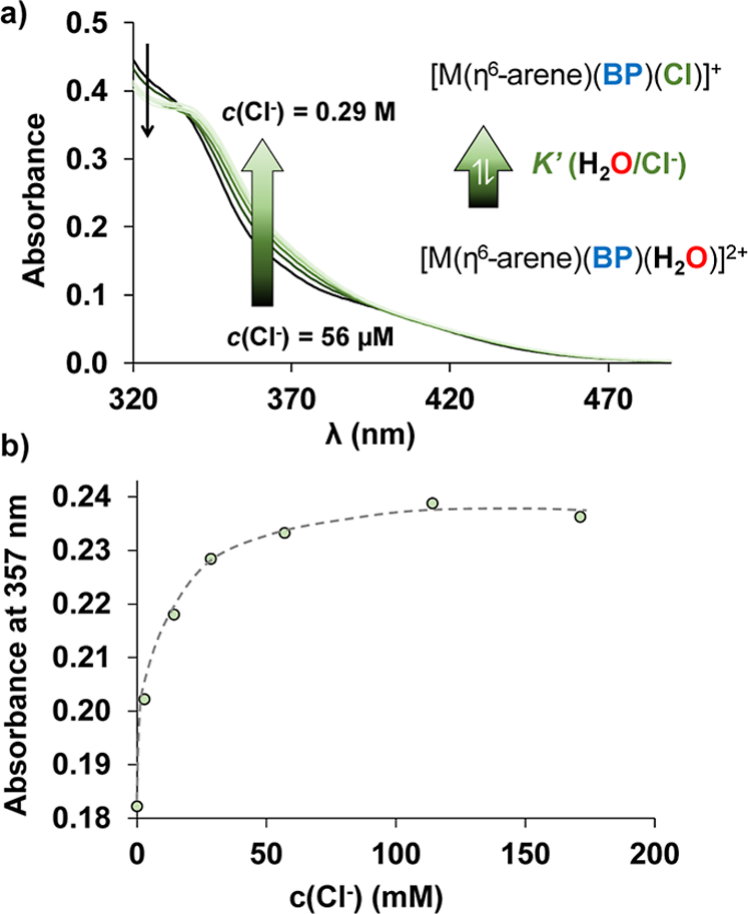
(a) Changes in the UV–vis spectra of complex RuCymBP due to the H_2_O/Cl^–^ coligand exchange process, (b) with absorbance variation at 357 nm plotted as a function of the chloride concentration (○) with the fitted curve (dashed line). Individual samples were prepared and the spectra were recorded after 1 h waiting time. {*c*_complex_ = 28 μM; *c*_Cl_– = 56 μM – 0.29 M; *t* = 25 °C; pH = 6.0 (20 mM phosphate buffer)}.

**Table 1:** Equilibrium Constants Related to the H_2_O/Cl^–^ Co-Ligand Exchange Process and the Deprotonation of the Coordinated Aqua Ligand in the [M­(η^6^-Arene/η^5^-arenyl)­(ligand)­(H_2_O)]^2+^ Complexes (Obtained under Chloride-Free Conditions)^c^

	constant	BP	DCP	PHEN^a^
RuCym	p*K*_a_ [ML(H_2_O)]	7.57 ± 0.06	7.21 ± 0.06	7.59
	log*K*′ (H_2_O/Cl^–^)	2.31 ± 0.13	2.31 ± 0.03	1.79
RhCp*	p*K*_a_ [ML(H_2_O)]	8.81 ± 0.06	8.33 ± 0.05	8.58
	log*K*′ (H_2_O/Cl^–^)	2.98 ± 0.01	2.85 ± 0.01^b^	2.92

a Data taken from ref 14.

b 2.89, predicted on the basis of the SC-XRD data, using the equation established in our previous work.^41^

c Corresponding data for the complexes of PHEN are also shown for comparison. {*t* = 25 °C}.

The *K*_a_ values of the complexes with BP and DCP ligands for the [M­(η^6^-arene/η^5^-arenyl)­(ligand)­(H_2_O)]^2+^ ⇌ [M­(η^6^-arene/η^5^-arenyl)­(ligand)­(OH)]^+^ + H^+^ process also provide valuable insights into their behavior in aqueous solutions. In order to determine the *K*_a_ values, UV–vis spectra were recorded at different pH values under chloride-free conditions, as shown for the complex RuCymDCP in Figure 5, and the determined p*K*_a_ values are presented in Table 1. These studies showed that the coordinated aqua ligand remained protonated in the pH range of 2–6, as no spectral changes were observed (i.e., no ligand release or deprotonation took place). However, at higher pH values, spectral shifts indicate the onset of aqua ligand deprotonation. For RuCym complexes, the calculated p*K*_a_ values (Table 1) indicate moderate acidity and a higher tendency for deprotonation at physiological pH compared with the RhCp* complexes. Notably, the p*K*_a_ value reported for the IrCp* complex of PHEN is 7.74.^17^ This lower value is most likely due to the stronger tendency of IrCp*, as well as RuCym, to undergo hydrolysis compared to RhCp*.^51^ Among these compounds, including the PHEN complexes, the DCP complexes consistently exhibit slightly lower p*K*_a_ values compared to those of their BP and PHEN counterparts, likely due to the stronger electron-withdrawing effect of the DCP ligand, which stabilizes more the mixed hydroxido complexes.

**Figure 5:**
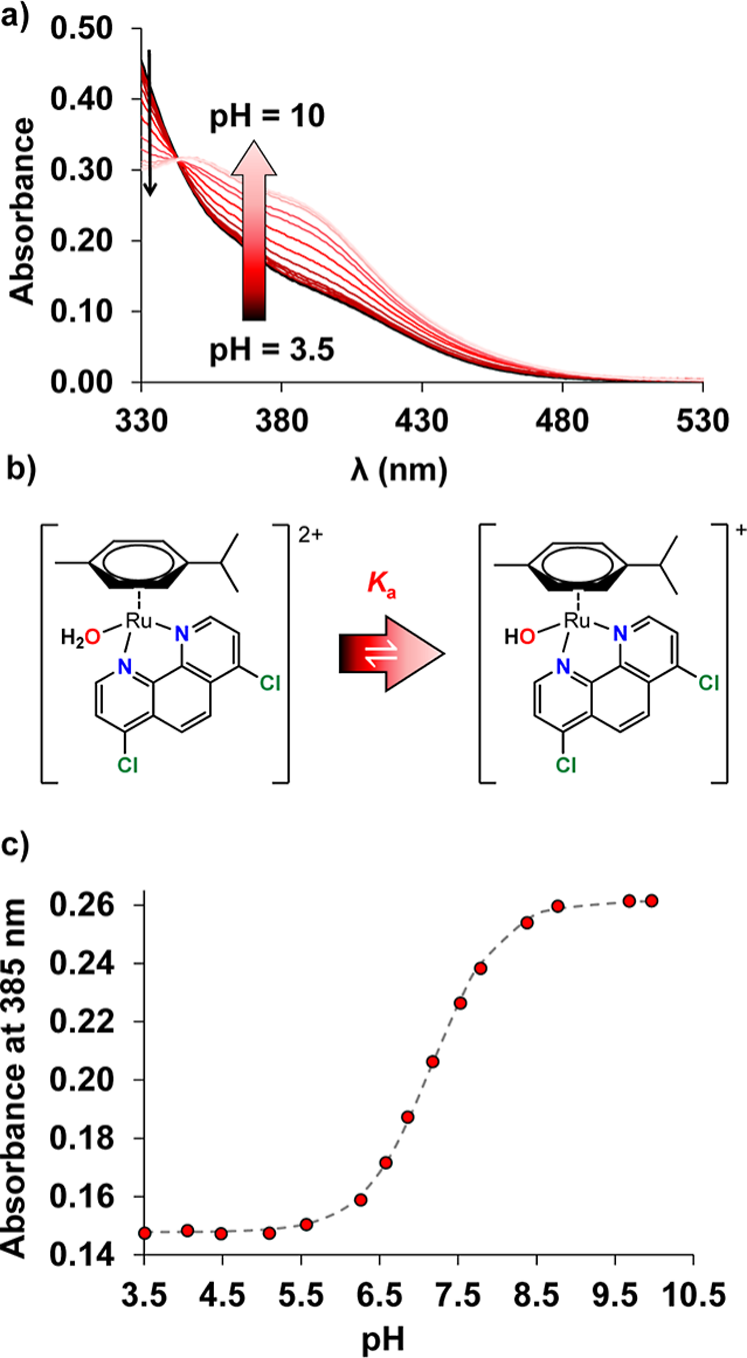
(a) UV–vis spectral changes observed for (b) the deprotonation of complex [RuCym­(DCP)­(H_2_O)]^2+^ and (c) the absorbance variation at 385 nm (•) together the fitted curves (dashed line) as a function of pH illustrating the p*K*_a_ determination. {*c*_complex_ = 71 μM; *I* = 0.2 M KNO_3_; *t* = 25 °C}.

Of note, for all the subsequent studies, the complexes in their [M­(η^6^-arene/η^5^-arenyl)­(ligand)­Cl]­Cl forms were used and mentioned as RuCymBP, RuCymDCP, RhCp*BP, RhCp*DCP for simplicity.

The bioactive compounds must cross the cell membrane, which is influenced by their lipophilicity. Therefore, we characterized the lipophilicity of the complexes using *n*-octanol–water partitioning at three different chloride ion concentrations at pH 7.4. As the H_2_O/Cl^–^ exchange alters the charge of the complexes, it consequently affects their lipophilicity. The ratio of chlorido and aqua complexes depends on the actual Cl^–^ concentration and the chloride affinity of the complex. Chloride ion concentrations were chosen to mimic physiological conditions, specifically those found in blood (100 mM), cytosol (24 mM), and the nucleus (4 mM). The distribution coefficients (*D*), presented in Table 2, show that as the Cl^–^ concentration decreases (leading to the formation of more aqua complexes), the log*D* values and consequently the lipophilicity of the complexes are also decreased. The overall trend indicates that the RuCym complexes exhibit higher lipophilicity compared with the RhCp* counterparts (Table 2), whereas the IrCp*BP complex was reported to be less lipophilic (log*P* = +0.7)^24^ than the RhCp* analogue. Notably, regardless of the chloride ion concentration, the lipophilicity of these complexes is consistently lower than that of the ligand alone (Table 2). Thus, we can conclude that complex formation unequivocally enhanced the hydrophilicity and aqueous solubility, which are important features for in vivo applications.

**Table 2:** Distribution Coefficients (*D*) of the Synthesized Metal Complexes and Their Corresponding Ligands, Determined at Three Different Chloride Concentrations^b^

log*D* values for	*c*(Cl^–^) = 4 mM	*c*(Cl^–^) = 24 mM	*c*(Cl^–^) = 100 mM
RuCymBP	+1.46 ± 0.02	+1.97 ± 0.02	+2.48 ± 0.02
RhCp*BP	+0.82 ± 0.01	+1.35 ± 0.03	+1.74 ± 0.02^a^
RuCymDCP	–0.55 ± 0.04	+0.04 ± 0.12	+0.35 ± 0.12
RhCp*DCP	–1.03 ± 0.03	–0.59 ± 0.02	–0.38 ± 0.02
BP			+3.69 ± 0.11
DCP			+2.40 ± 0.08

a IrCp*BP complex: log*P* = + 0.7 (100 mM NaCl).^24^

b {*n*-Octanol/20 mM phosphate buffer, pH = 7.4; *t* = 25 °C}.

### HSA Binding of the Metal Complexes

Several drugs bind reversibly to blood proteins within the vascular system, which influences their distribution, elimination, and metabolism, as macromolecule-bound compounds are often “invisible” for the metabolic pathways. However, there is an increasing body of evidence suggesting that small molecules bound to HSA are actively transported via gp60-mediated transcytosis in vivo,^52^ and the EPR effect also helps the tissue penetration of HSA-bound drugs.^53^ This latter phenomenon is particularly useful to deliver anticancer agents, as the vascular network of cancerous tissue is often perforated, and the drainage of the interstitial fluid is impaired by the incomplete lymphatic network. Therefore, understanding the drug–HSA interaction is essential. The HSA-binding of the title complexes was followed by the combined use of UV–vis and ^1^H NMR spectroscopy, spectrofluorometry, and capillary zone electrophoresis (CZE).

UV–vis is a simple technique to follow the interaction of half-sandwich metal complexes with HSA in time. Charge transfer bands of the complexes were monitored over a 12 h period (as shown for the RhCp*BP – HSA (1.5:1) system in Figure S16). An initial rapid spectral change can be observed, which is followed by a slower spectral evolution. This phenomenon may indicate (i) a rapid binding interaction followed by a slow rearrangement of the coordination sphere around the Rh center or (ii) that the differing accessibility of binding sites results in biphasic binding kinetics. A similar trend was noted for RhCp*DCP complex in the presence of HSA (Figure S17). In contrast, minimal spectral changes were detected for the RuCym complexes. However, this observation alone does not exclude binding of the latter complexes to HSA under the applied conditions. To further substantiate this observation, CZE was applied. In Figure 6, the peak of the free RhCp*BP complex is clearly separated from the peaks of the comigrating protein and the protein-bound complex. The gradual decrease in the peak area of the free metal complex indicates that binding is not instantaneous. Whereas *ca*. 50% of the complex is protein-bound after the first 5 min, the equilibration takes ca. 3–4 h. This observation confirms that the accessibility of binding sites for the complexes is not equal in HSA. After 4 h, the concentration of free RhCp*BP decreased to 7%, suggesting that a single albumin molecule can accommodate at least two RhCp*BP complexes. In the case of RhCp*DCP, 21% of the metal complex remained unbound after 24 h, indicating a lower binding affinity compared to that of RhCp*BP, although at least two binding sites are available for the DCP complex as well. CZE measurements demonstrated no significant binding of the RuCym complexes to albumin, even after a 24 h incubation period (Figure S18).

**Figure 6:**
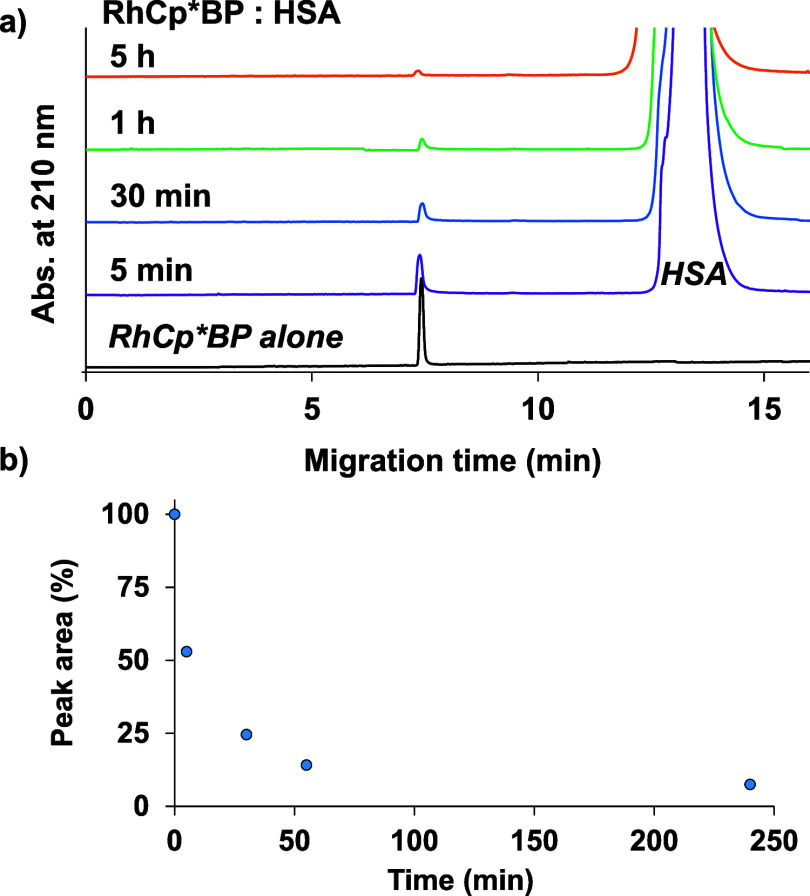
(a) Electropherograms of the RhCp*BP complex in the absence and presence of 0.5 equiv HSA at pH = 7.4 followed in time and (b) the relative peak area of free RhCp*BP at 210 nm (migration time: 7.4 min) as a function of incubation time. {*c*_complex_ = 95 μM, *c*_HSA_ = 48 μM; *U* = 8 kV; current = 105 μA; PBS’; *t* = 25 °C}.

Notably, CZE measurements revealed no release of free BP or DCP ligands in the electropherograms, suggesting indirectly that the protein binding is associative; the bidentate ligands remain coordinated to the metal ion. This observation underscores the stable nature of the metal–ligand coordination bonds in these complexes.

Fluorescence spectroscopy was utilized to elucidate the binding sites on HSA that are targeted by the metal complexes. This investigation concentrated on two key aspects: the quenching of HSA’s intrinsic fluorescence attributed to the sole tryptophan residue (Trp-214) and the displacement of site-specific fluorescent markers warfarin (WF) and dansylglycine (DG). Trp-214 is situated in Sudlow’s site I within the IIA subdomain, rendering it a valuable probe for evaluating interactions at this site. Fluorometric measurements, conducted over a 5 h time frame (see example for RhCp*BP in Figure S19), corroborated the results of UV–vis experiments, the quenching of fluorescence took several hours for the RhCp* complexes, while RuCym compounds had no remarkable effect of Trp-214 fluorescence. Quenching studies were conducted over a 24 h incubation period. This time frame is most probably not enough to reach equilibrium with RuCym complexes; however, this is considered as the upper limit that can be physiologically relevant, as longer incubation times are rendered irrelevant by metabolism and elimination. Notably, for the RuCym complex of PHEN, no measurable quenching was reported within 24 h,^11^ and the complex [Ru­(PHEN)_3_]^2+^ also exhibited negligible interaction with HSA.^54^ Among the four metal complexes investigated, the most pronounced quenching effect was observed for RhCp*BP (Figure 7a), where the initial intensive fluorescence of Trp-214 is almost completely quenched totally upon the addition of 20 equiv of the metal complex. Reduced quenching efficiency was observed for the other compounds in the following order: RhCp*DCP > RuCymBP > RuCymDCP (Figure 7b). Quenching constants (KQ^′^) were computed using HypSpec software.^55^ The quenching constants reflected the formerly observed trend, with RhCp*BP displaying the highest binding affinity (log*K*_Q_^′^ = 5.5), approaching the quenching constant of the precursor RhCp* (log*K*’_Q_ = 5.8^56^). This finding implies that complex RhCp*BP possesses a high affinity for HSA. It should be noted that quenching constants calculated for the RuCym complexes represent only a temporary state at 24 h of incubation time.

**Figure 7:**
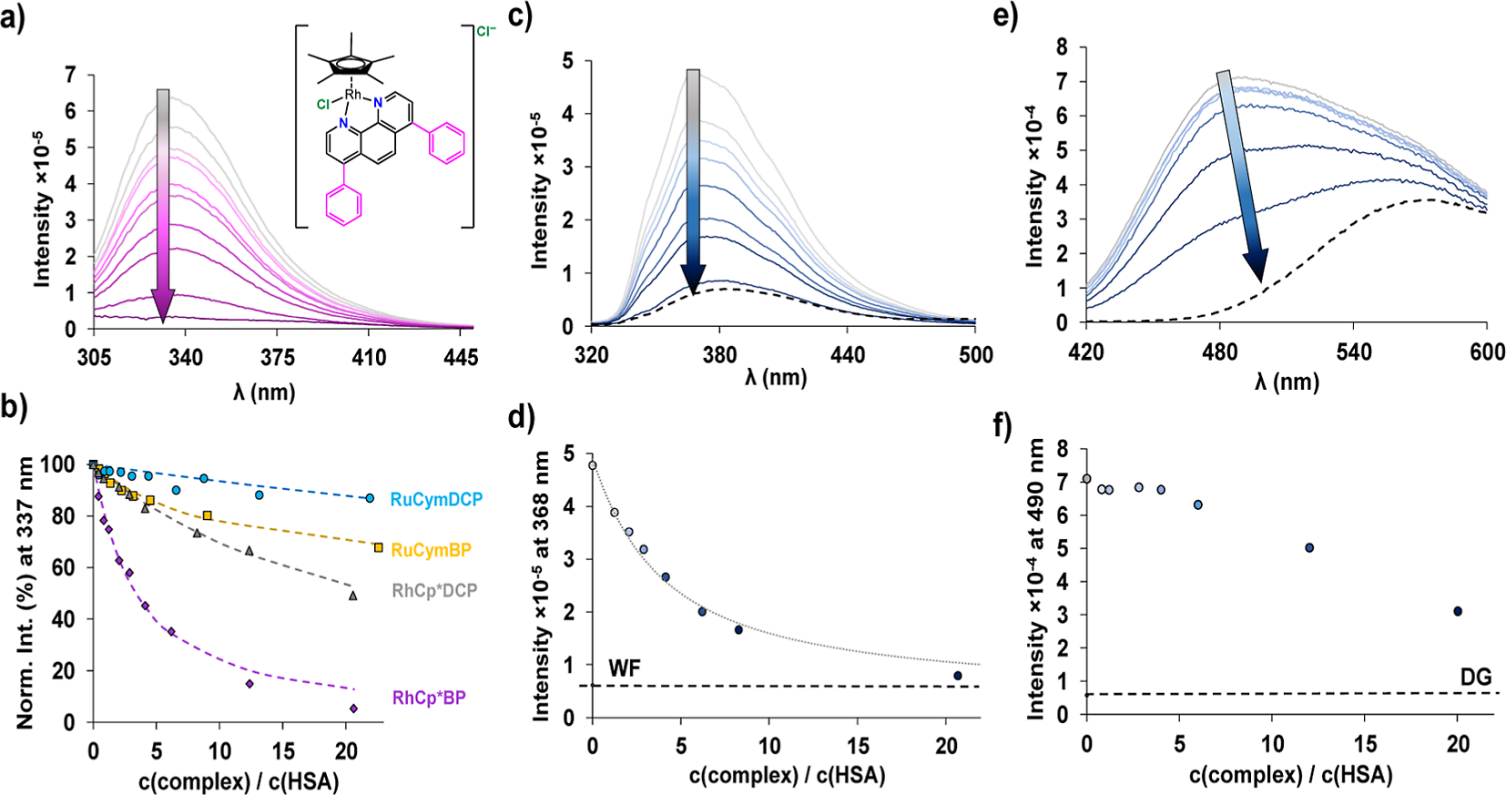
Binding interactions of RhCp*BP with HSA analyzed via fluorescence quenching and marker displacement assays. (a) Trp214 fluorescence quenching by RhCp*BP. (b) Measured (dots) and fitted (dashed lines) quenching curves for RhCp*BP, RuCymBP, RhCp*DCP, and RuCymDCP. Fluorescence spectra of the (c) HSA–WF 1:1 and (e) HSA–DG 1:1 systems by the addition of RhCp*BP (dashed lines indicate the free site marker) and the corresponding fluorescence intensity curves at the indicated wavelengths (d,f). The dotted line in figure (d) denotes the fitted intensities. {*c*_HSA_ = 1.2 μM; *c*_marker_ = 0 or 1.2 μM PBS’; *t* = 25 °C}.

To further explore the binding affinity and site specificity of the metal complexes, site marker displacement experiments were conducted using WF and DG, which bind to Sudlow’s site I (in IIA subdomain) and to Sudlow’s site II (in IIIA subdomain), respectively. The displacement of these fluorescence probes directly confirms the interactions of the metal complexes with the respective binding sites. The displacement constants of WF (KWF′) by the metal complexes (Figure 7c,d, Table 3) were correlated with the Trp-214 quenching data. RhCp*BP demonstrated the highest binding affinity for Sudlow’s site I. RhCp*DCP and RuCymBP exhibited moderate displacement, while RuCymDCP showed the weakest effect. It should be noted that the title RhCp* complexes displayed stronger binding to HSA at site I than the corresponding complexes of PHEN and 2,2′-bipyridine (BPY) (Table 3).

**Table 3:** Quenching Constants (log*K*_Q_^′^) and WF Displacement Constants (log*K*_WF_′) for the Investigated Metal Complexes and the Organometallic Precursors^c^

	log*K*_Q_′	log*K*_WF_′
RuCym precursor	5.7^a^	5.9^a^
RuCymDCP	≤3.8	≤4.3
RuCymBP	4.3 ± 0.1	4.4 ± 0.1
RhCp*precursor	5.8^a^	6.1^a^
RhCp*DCP	4.6 ± 0.1	4.8 ± 0.1
RhCp*BP	5.5 ± 0.1	5.6 ± 0.1
RhCp*BPY	3.5–4.1^b^	3.5–4.1^b^
RhCp*PHEN	3.5–4.1^b^	3.5–4.1^b^

a Data taken from ref 56.

b Data taken from ref 11.

c {PBS’; *t* = 25 °C}.

DG displacement experiments indicated no significant interaction with Sudlow’s site II for three of the complexes, RuCymBP, RuCymDCP, and RhCp*DCP, under the experimental conditions applied. However, RhCp*BP was able to displace the DG (Figure 7e), suggesting some degree of interaction with this site. The binding curve for this interaction exhibited a sigmoidal shape (Figure 7f), which hindered the calculation of the binding constant. The observed trend indicates that Sudlow’s site II is not the primary binding site for RhCp*BP but may function as a secondary binding pocket. These results affirm that Sudlow’s site I serves as the primary binding site for RhCp*BP.

Our previous studies conducted on related RhCp* complexes of BPY and PHEN with peptide models strongly suggest that histidine imidazole nitrogens are likely coordinating partners in HSA.^11,56^ Therefore, the interactions between 1-methylimidazole (MeIm) and the metal complexes RhCp*BP and RuCymBP were examined using ^1^H NMR spectroscopy in PBS’ buffer at pH 7.4. Our other aim was to investigate the binding kinetics of RuCymBP over a longer period of time, since MeIm has no such stability problems compared to HSA in a 2 week follow-up.

Analysis of the ^1^H NMR spectra recorded of the RuCymBP—MeIm system (Figure S20) showed that the dual peak set of free RuCymBP is due to the coexistence of aqua, hydroxido, and chlorido complexes in the sample. Slow exchange of the aqua and chlorido complexes on the NMR time scale is not unique for RuCym complexes; formerly we have observed the same phenomenon with 2-picolinate and cyclometalated 4-phenylthiazole complexes.^56,57^ The ternary complex formation with MeIm is rather slow; at a 1:1 ratio, the emergence of a new peak can be observed after 6 days, and only *ca*. 10% ternary complex forms after 14 days. By increasing the MeIm excess to 10-fold, the ternary complex becomes detectable after 8 h and about 2/3 of the metal complex transforms to the ternary species after 14 days. ^1^H NMR studies on the RhCp*BP – MeIm system indicated a more rapid and almost quantitative interaction (Figure S21). The formation of the ternary complex proceeds within 30 min with 87% conversion. The quantity of the ternary complex formed with MeIm agrees very well with previously reported data on RhCp*BPY—MeIm (67%), although the complex formation was reported to be slower for the latter.^50^ The coordination of the present RhCp* complexes to His imidazole groups in albumin is therefore highly probable; however, the specific binding sites and the possible involvement of other side chains remain to be elucidated in future studies.

As a summary of the results obtained for the albumin binding of the present complexes, we can highlight some key findings. RhCp* complexes bind to HSA more effectively than the RuCym congeners, and the BP complexes seem to bind stronger than the DCP complexes. Binding constants computed for the RuCym complexes are conditional also in terms of incubation time since the samples did not reach thermodynamic equilibrium even after 24 h. The CZE experiments prove the binding of at least 2 equiv of RhCp*BP and RhCp*DCP on HSA. Hydrophobic binding sites I and II were identified as primary and secondary binding sites of RhCp*BP, respectively. ^1^H NMR measurements conducted with the histidine model MeIm confirm that the diminished binding affinity of RuCym complexes arises from kinetic limitations, as suggested in earlier investigations for RuCymBPY and RuCymPHEN complexes.^11^ In contrast, RhCp*BP demonstrated strong binding to HSA (log*K*_Q_^′^ = 5.5), and also RhCp*DCP binds in a remarkable manner (log*K*_Q_^′^ = 4.6). For comparison, much weaker quenching was reported for RhCp*BPY and RhCp*PHEN (log*K*_Q_^′^ values ranging from 3.5 to 4.1).^11^ This disparity seems to be connected to the different lipophilicity of the complexes; RhCp*BP exhibits the strongest binding and the highest lipophilicity (log*D*_7.4_ = +2.48), while the strongly hydrophilic RhCp*BPY (log*D*_7.4_ = –1.8) displayed the weakest interaction.^11^ By taking a closer look, we see that lipophilicity indirectly depends on the H_2_O/Cl^–^ exchange constant of the complexes as well, and these results highlight the pivotal role of lipophilicity and chloride affinity in the albumin-binding ability of these metal complexes.

### Nanoformulation

Our approach to improve the pharmacological properties of the anticancer organic compounds involves two complementary strategies. Metal complexation with RhCp* enhanced the aqueous solubility and binding affinity for HSA. In addition, nanoformulation increases bioavailability by protecting the complex from premature reactions and might facilitate tumor accumulation via the EPR effect. Furthermore, an increased circulation time is expected as well. Options include liposomes, polymeric nanoparticles, and protein-based carriers, all of which enhance drug delivery and therapeutic efficacy by improving the stability, extending the circulation time, and promoting selective accumulation at tumor sites. Protein-based carriers, such as HSA-based nano-, and colloidal particles, are particularly promising due to their biocompatibility, ability to extend drug circulation, and improve tumor-specific delivery.^58–60^ Pt­(IV) complexes have been successfully encapsulated into nanoparticles using HSA. These particles selectively accumulated in tumor tissue and demonstrated significant tumor growth inhibitory effects against osteosarcoma in a mouse model.^61^ Certain half-sandwich RuCym complexes with a (N,N) donor set have emerged as innovative agents for treating hypoxic tumors such as osteosarcoma and have been engineered into polymeric nanoreactors. Reports suggest that these complexes can nearly eradicate tumors in mouse models, representing a significant advancement in targeted cancer therapy.^62^

Given the remarkable binding of RhCp* complexes displayed to HSA, the selected formulation contains glutaraldehyde cross-linked HSA or HSA with the PLGA copolymer as a macromolecular-based colloidal carrier system, which is stabilized by TPGS with its amphipathic character. TPGS was included to enhance cellular uptake and to take advantage of its intrinsic anticancer activity.^29,30,63^ On the other hand, PLGA provides controlled, sustained release while protecting the active complex.

Complex [RhCp*­(BP)­Cl]Cl was selected for nanoformulation with glutaraldehyde cross-linked HSA and HSA–TPGS–PLGA (HTP), due to its high cytotoxicity and MDR-selectivity. To optimize the formulation parameters, different drug-to-carrier ratios were used. The nanoformulated products were characterized by their hydrodynamic diameter (*d*_H_) and ζ-potential using NanoParticle Analyzer equipment (see Figure 8 for the HSA-based nanocarrier system), while transmission electron microscopy (TEM) (see Figure 9 for the HSA-based nanoparticles) enabled direct visualization of the particles and their homogeneity. Additionally, the encapsulation efficiency (EE %) was determined using UV–vis spectroscopy. The optimized conditions and calculation methods are shown in the Experimental part.

**Figure 8:**
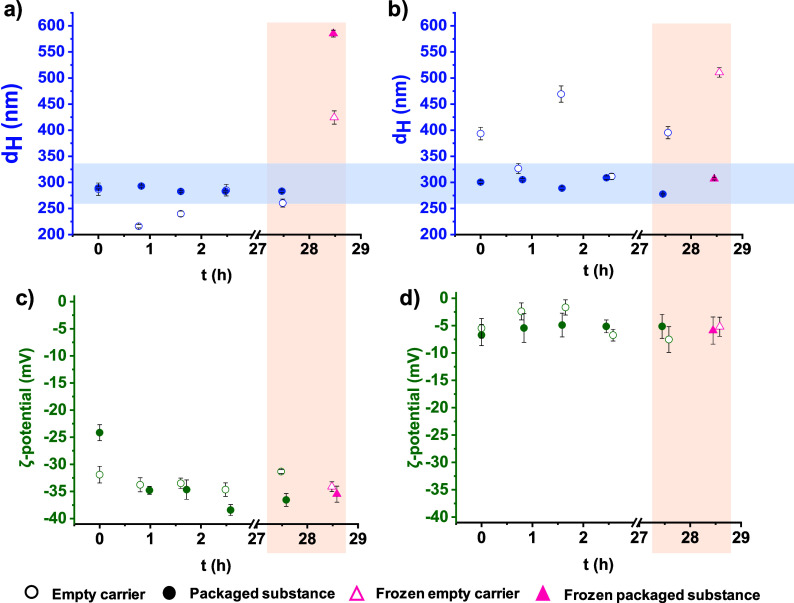
Hydrodynamic diameter (*d*_H_) and ζ-potential of empty (○) and RhCp*BP-loaded (●) HSA-based nanocarrier systems measured (a,c) in pure water and (b,d) in PBS’ buffer (after 10-fold dilution and at 1:1 drug-to-carrier ratio).

**Figure 9:**
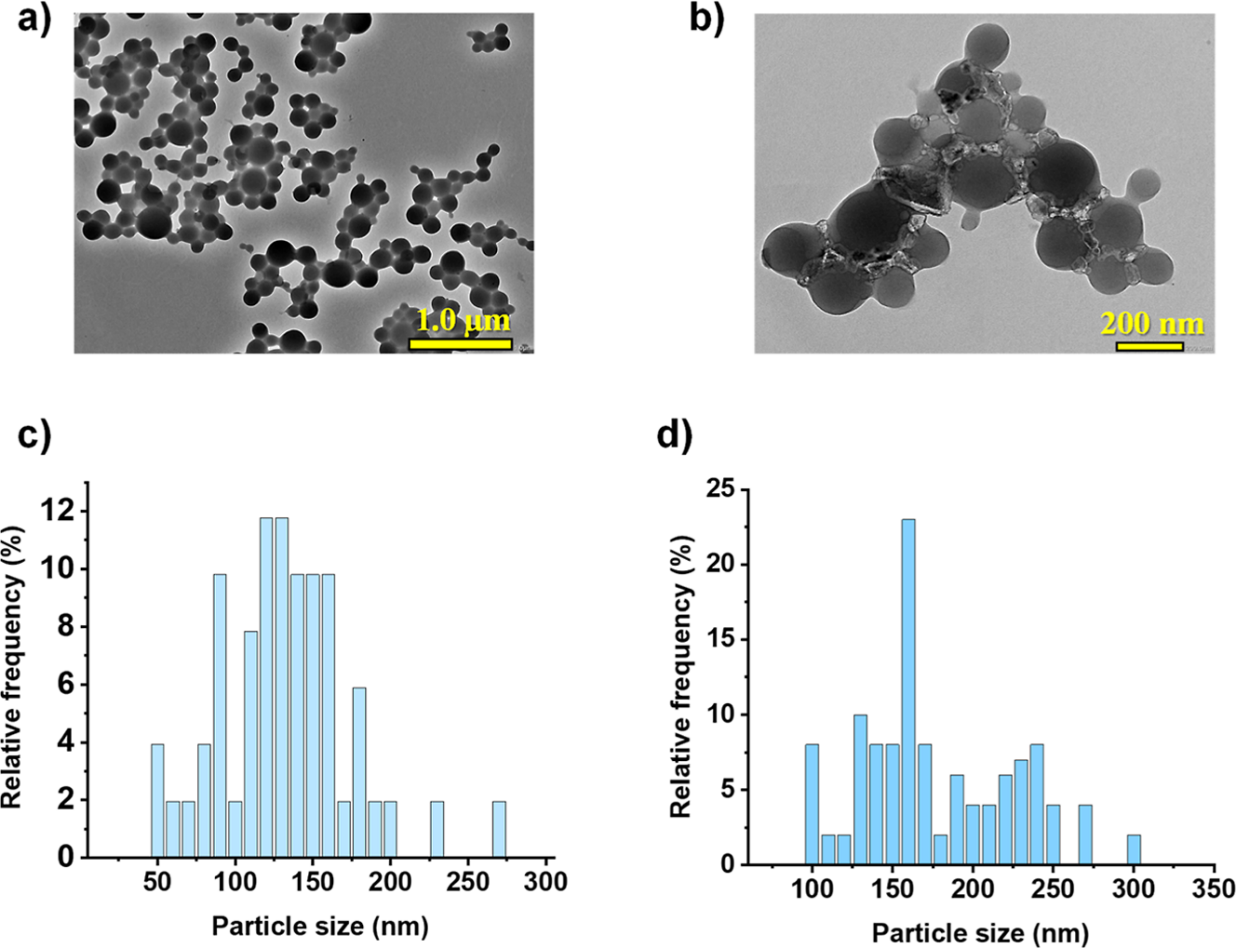
(a,b) Representative TEM images and (c,d) size distribution diagrams of RhCp*BP-loaded HSA carriers (1:1 drug-to-carrier ratio) in (a,c) pure water and (b,d) PBS’ buffer. The scale bars in (a,b) are 1.0 μm and 200 nm, respectively.

HSA-based carriers were prepared by dissolving HSA in a solution of the complex (HSA/RhCp*­(BP) molar ratio of 1:2), followed by ethanol precipitation and glutaraldehyde cross-linking to form the carrier structure. The formulation was purified via centrifugation and redispersed in water by using ultrasonication. Using the nanoprecipitation technique, HTP carriers (HSA/PLGA/TPGS at 1:1:0.5 mass ratio) were prepared by sequentially mixing HSA dissolved in aqueous solution of 2 equiv of RhCp* and TPGS, followed by dropwise addition of PLGA in acetone. The formulation was then centrifuged, purified, and redispersed in water.

The presented hydrodynamic diameter (*d*_H_) and ζ-potential data (Figure 8) illustrate the characterization of HSA-based nanocarrier systems measured in two different aqueous media (water and PBS). Hydrodynamic diameter measurements revealed that the RhCp*BP-loaded carriers remain stable over time in both media with sizes consistently ranging between 250 and 350 nm. This finding was supported by TEM images, which confirm size distributions predominantly between 100 and 200 nm (Figure 9). It should also be noted that the empty carriers exhibited less stability over time. This suggests that the incorporated drug stabilizes the carriers, likely through drug–carrier interactions that reinforce the structural integrity.

Electrokinetic potential studies indicate highly negative ζ-potential values in water, reflecting strong electrostatic stability. In PBS’ buffer, however, ζ-potential values are less negative due to salt-induced charge screening, highlighting that colloidal stability is not solely dependent on ζ-potential. To investigate the effect of freezing-thawing, samples in both media were frozen (−20 °C) and analyzed after 24 h, comparing their hydrodynamic diameter and ζ-potential to nonfrozen samples. In water, freezing led to an increase in size for both drug-loaded and drug-free formulations, indicating aggregation or other structural changes. In PBS’ buffer, however, the drug-loaded frozen formulations preserved their stability, demonstrating resilience against freezing-induced effects.

It was crucial to find the most ideal complex-to-HSA ratio for the formulation, and the 2:1 drug-to-carrier ratio was determined to be optimal based on our binding and formulation data. Namely, the CZE studies indicated that at least 2 equiv of RhCp*BP could bind to HSA, though the upper limit remains undetermined. At this ratio, the dynamic light scattering (DLS) results revealed a hydrodynamic diameter of 532 ± 16 nm, and the zeta potential was found to be −92.2 ± 1.6 mV (Figure 10a), indicating sufficient colloidal stability. The increased particle size at this ratio may confer advantages for cancer therapy by enhancing tumor selectivity via the EPR effect. As shown in Figure 10a, aggregation was not observed up to a 2:1 drug-to-carrier ratio in HSA-based formulations. However, at higher drug-to-carrier ratios (e.g., 3.2:1 and 6.2:1), aggregation was observed, as evidenced by the turbidity changes shown in Figure 10a, rendering these formulations unsuitable. To prevent aggregation and ensure stability, the 2:1 ratio was used for further studies. The encapsulation efficiency (EE %) followed an increased curve, reaching 97% at a 2.6:1 ratio, confirming effective drug incorporation into the glutaraldehyde cross-linked HSA carrier.

**Figure 10:**
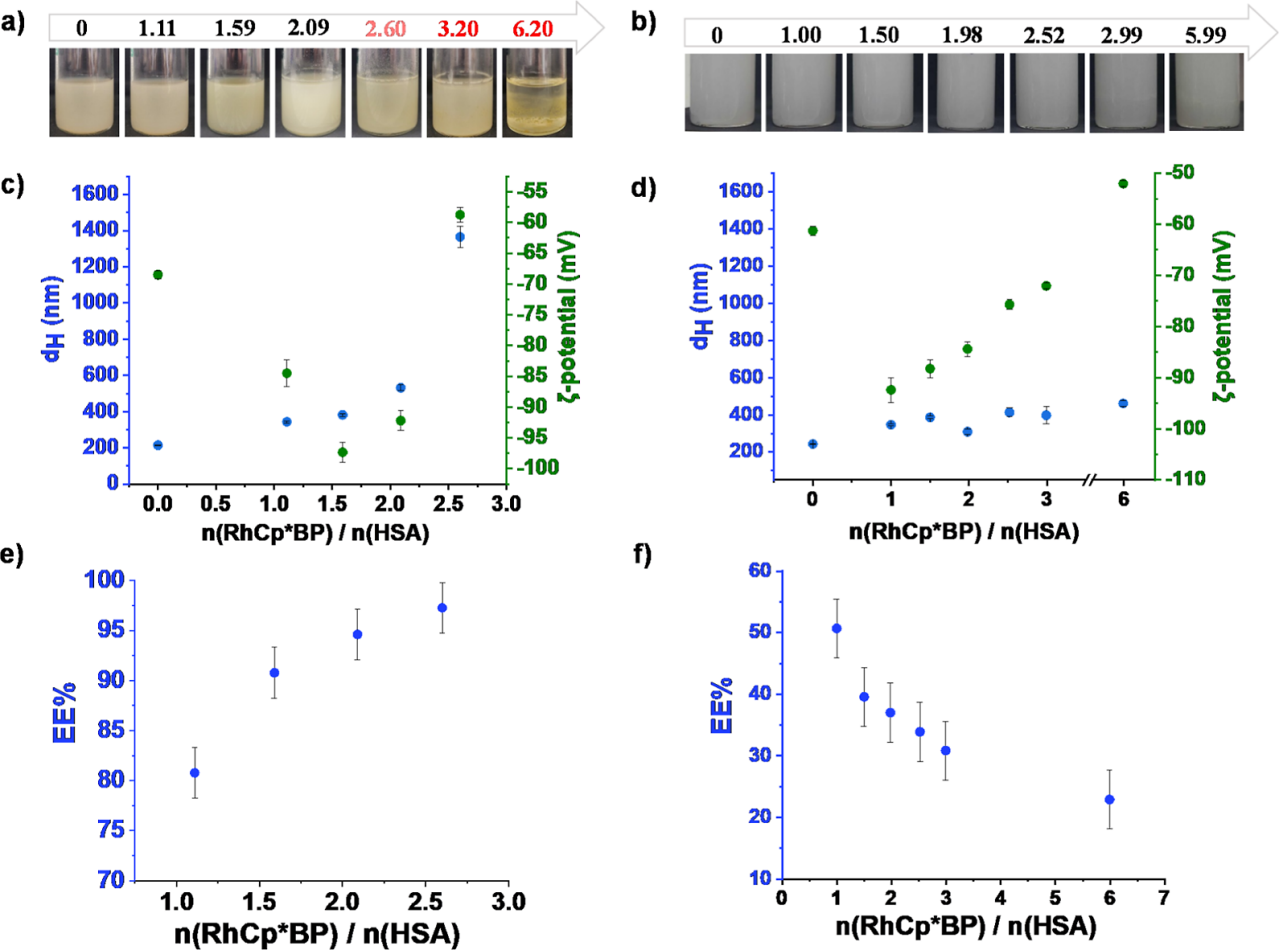
Photographs of RhCp*BP-containing (a) HSA and (b) HTP formulations at varying drug-to-carrier ratios. Hydrodynamic diameter (*d*H) and ζ-potential as a function of drug-to-carrier ratios for (c) HSA- and (d) HTP-based formulations (after 10-fold dilution). Encapsulation efficiency (EE %) of RhCp*BP for (e) HSA and (f) HTP formulations at different drug-to-carrier ratios.

For the HTP-based formulations, the 2:1 drug-to-carrier ratio also emerged as the optimal choice, yielding a hydrodynamic diameter of 309 ± 15 nm and a zeta potential of −84.4 ± 1.4 mV (Figure 10d), indicating a stable colloidal system. However, the encapsulation efficiency was significantly lower compared with HSA, reaching only 37% at a 1.98:1 ratio, which was decreased further at higher ratios. These findings highlight the superior encapsulation capacity of HSA over that of HTP for RhCp*BP delivery.

The release behavior of RhCp*BP in PBS’ buffer (pH = 7.4) was monitored using a modified equilibrium dialysis device (Figures S22 and S23), and the absorbance (proportional to the complex concentration) was measured for the dialysate phase by online UV–vis spectrophotometry over a period of 16 h. The nonformulated RhCp*BP complex was observed to diffuse freely into the PBS’ medium, requiring approximately 9 h for complete diffusion. In contrast, no detectable release of the RhCp*BP complex was observed from either carrier (HSA, HTP) within the 16 h monitoring period. Moreover, extended monitoring over a 3 day period confirmed that no significant release occurred from either the HSA- or HTP-based formulations, demonstrating the exceptional stability and retention of the complex within these carriers.

In summary, cross-linked HSA and HTP carriers both demonstrate potential as drug delivery systems with notable differences. HSA formulations offer a higher encapsulation efficiency (97%) and larger particle sizes at a 2.5:1 drug-to-HSA ratio. In contrast, HTP formulations tolerate higher applied drug-to-carrier ratios without significant aggregation, providing greater flexibility despite a lower encapsulation efficiency (37%) (at the same drug-to-HSA ratio). Both nanocarrier systems exhibited high stability and effectiveness to retain the RhCp* complex, highlighting their suitability for a potential in vivo application.

### Cytotoxicity and MDR Selectivity of the Complexes and Their Ligands

The PHEN-derived ligands (DCP, BP) (together with PHEN for comparison) and the isolated complexes ([RuCym­(DCP)­Cl]­Cl, [RhCp*­(DCP)­Cl]­Cl, [RuCym­(BP)­Cl]­Cl, [RhCp*­(BP)­Cl]­Cl) were tested against the estrogen receptor (ER) negative MDA-MB-231, and the ER positive MCF-7 and T-47D breast cancer cell lines (Table 4, and Figure 11a for easier comparison). PHEN and DCP ligands were moderately cytotoxic (IC_50_ values were between 3.2 – 8.6 and 3.8–5.1 μM, respectively), while BP exhibited submicromolar cytotoxicity (0.59–0.76 μM) against all three breast cancer lines, exceeding the relative toxicity of cisplatin (3.2–7.0 μM). Compared to the free ligands, [RhCp*­(BP)­Cl]­Cl, and to a lesser extent, [RhCp*­(DCP)­Cl]Cl showed decreased cytotoxicity (4.7–14.9 μM and 5.9–7.6 μM, respectively), while complexation with RuCym decreased the cytotoxicity only slightly compared to BP (1.2–1.9 μM) but deteriorated the cytotoxicity compared to DCP (>75 μM).

**Table 4:** Cytotoxicity of the Ligands PHEN, BP, DCP and the Isolated Complexes ([RuCym­(DCP)­Cl]­Cl, [RhCp*­(DCP)­Cl]­Cl, [RuCym­(BP)­Cl]­Cl, [RhCp*­(BP)­Cl]­Cl) against Breast Cancer Cell Lines^a^

	MDA-MB-231		MCF-7		T47D	
	IC_50_/μM	+SD/–SD	IC_50_/μM	+SD/–SD	IC_50_/μM	+SD/–SD
PHEN	3.20	0.38/0.34	8.57	1.05/0.93	5.47	3.44/2.11
BP	0.76	0.20/0.16	0.59	0.004/0.004	0.63	0.05/0.04
[RhCp*(BP)Cl]Cl	4.68	0.08/0.07	14.9	4.25/3.31	5.28	0.63/0.56
[RuCym(BP)Cl]Cl	1.23	0.05/0.04	1.93	0.04/0.04	1.86	0.67/0.49
DCP	5.09	0.14/0.14	3.84	0.40/0.36	4.29	0.63/0.55
[RhCp*(DCP)Cl]Cl	7.56	1.61/1.33	5.92	0.18/0.17	7.36	1.19/1.10
[RuCym(DCP)Cl]Cl	>100		76.4	5.66/5.27	74.99	12.1/10.4
cisplatin	3.16	1.86/1.17	4.78	2.23/1.52	6.98	3.23/2.21

a Cisplatin was used as the positive control. (pIC_50_ values with SD are found in Table S5).

**Figure 11:**
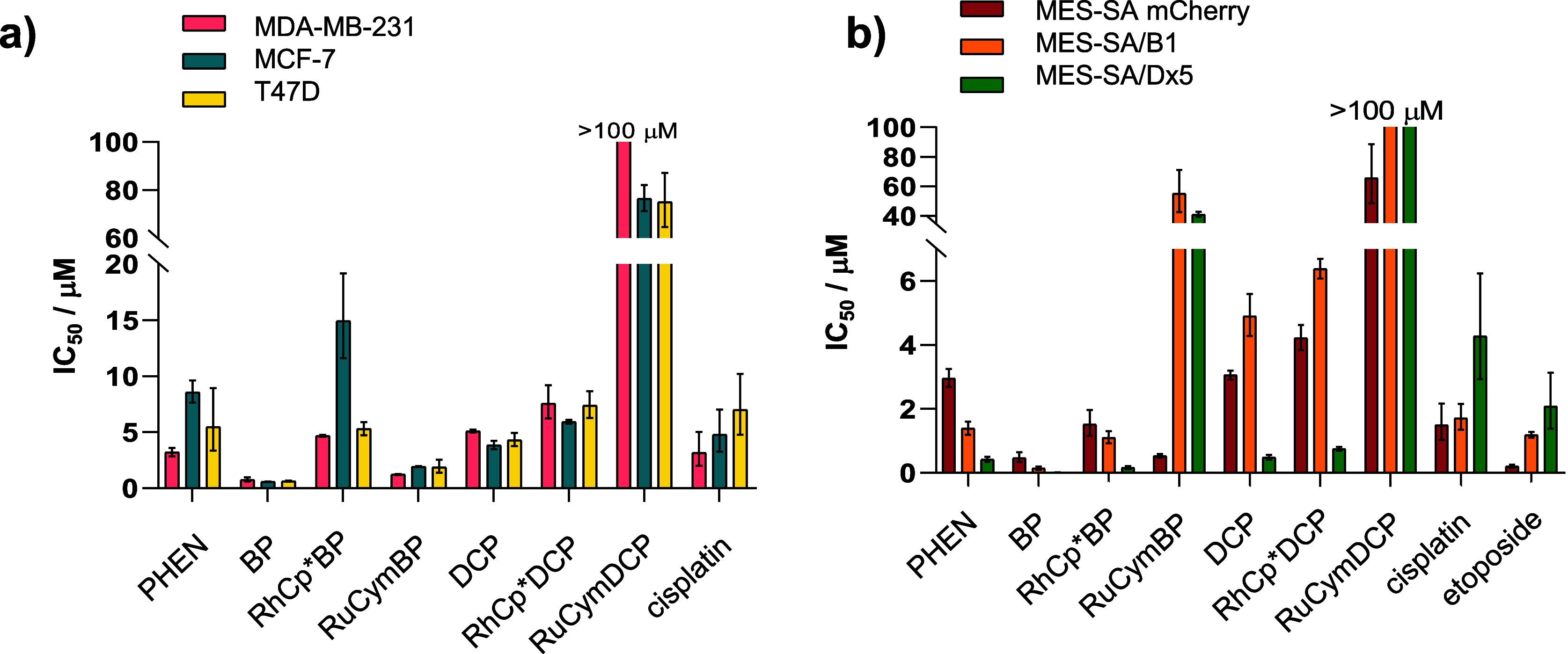
Cytotoxicity of the 1,10-phenanthroline ligands and their RhCp* and RuCym complexes against (a) breast cancer cell lines and (b) the MES-SA mCherry uterine sarcoma cell line and its MDR derivatives (MES-SA/B1-mOrange and MES-SA/Dx5-eGFP). Cisplatin and etoposide were tested as controls.

Cytotoxicity of the compounds were further tested also in an in vitro MDR model system, consisting of an MES-SA uterine sarcoma cell line, and its P-gp (ABCB1) overexpressing variants MES-SA/B1, that was genetically engineered to express P-gp, and MES-SA/Dx5, which acquired a P-gp-mediated MDR phenotype due to selection in doxorubicin.^8,63^ The expression of different fluorescent proteins in each line (mCherry, mOrange, or eGFP) allowed coculturing of these three cell lines, providing more relevant data compared to monocultures, as the interaction between parental and resistant phenotypes is reflected in cytotoxicity. The determined IC_50_ values are shown in Table 5 and Figure 11b.

**Table 5:** Cytotoxicity of the Ligands PHEN, BP, DCP, the Isolated Complexes ([RuCym­(DCP)­Cl]­Cl, [RhCp*­(DCP)­Cl]­Cl, [RuCym­(BP)­Cl]­Cl, [RhCp*­(BP)­Cl]­Cl), and the Cross-Linked HSA and HTP-Based Nanoformulated [RhCp*­(BP)­Cl]Cl against Parental and MDR Uterine Sarcoma Cell Lines^a^

	MES-SA mCherry		MES-SA/B1-mOrange		MES-SA/Dx5-eGFP		SR	SR
384-well plate	IC_50_/μM	+SD/–SD	IC_50_/μM	+SD/–SD	IC_50_/μM	+SD/–SD	(MES-SA/B1)	(MES-SA/Dx5)
PHEN	2.95	0.29/0.27	1.39	0.22/0.19	0.41	0.08/0.07	2.1	7.1
BP	0.47	0.18/0.13	0.14	0.07/0.04	0.010	0.002/0.002	3.4	54.7
[RhCp*(BP)Cl]Cl	1.56	0.31/0.26	1.37	0.33/0.26	0.23	0.09/0.06	1.1	6.7
[RuCym(BP)Cl]Cl	0.53	0.06/0.05	55.0	16.2/12.5	41.0	1.73/1.66	0.01	0.01
DCP	3.05	0.15/0.14	4.90	0.70/0.61	0.48	0.08/0.07	0.6	6.3
[RhCp*(DCP)Cl]Cl	4.22	0.41/0.37	6.38	0.32/0.30	0.75	0.06/0.06	0.7	5.6
[RuCym(DCP)Cl]Cl	65.6	23.1/17.1	>100		>100			
cisplatin	1.49	0.67/0.46	1.71	0.45/0.36	4.28	1.96/1.34	0.9	0.3
etoposide	0.20	0.05/0.04	1.19	0.09/0.09	2.08	1.05/0.70	0.2	0.1
96-well plate								
[RhCp*(BP)Cl]Cl	2.90	0.54/0.46	2.43	0.60/0.48	1.77	0.13/0.12	1.2	1.6
with cross-linked HSA	3.87	0.42/0.38	5.46	1.24/1.01	2.56	0.16/0.15	0.7	1.5
with HTP	1.46	0.35/0.28	3.25	0.86/0.68	1.58	1.16/0.67	0.4	0.9

a Cisplatin and etoposide were used as the positive control. (pIC_50_ values with SD are found in Table S6).

The pattern of cytotoxicity against the parental MES-SA line was consistent with that of the breast cancer lines, showing moderate cytotoxicity for PHEN and DCP (3.0 μM, 3.1 μM, respectively) and submicromolar activity for BP (0.47 μM). Cytotoxicity was reduced to a greater extent upon complexation with RhCp* and with BP (1.51 μM) than with DCP (4.2 μM). In contrast, complexation with RuCym preserved the cytotoxicity of BP (0.53 μM) but significantly reduced the toxicity of DCP (65.6 μM). The resistant lines, however, showed a different cytotoxicity pattern. PHEN, a known MDR-selective agent that targets resistant cells through the efflux function of P-gp,^14,64^ and BP were hypertoxic to both MES-SA/B1 and MES-SA/Dx5, compared to the parental line. DCP, however, was more active only against MES-SA/Dx5, and not against MES-SA/B1. This pattern remained unchanged upon complexation with RhCp*, with [RhCp*­(BP)­Cl]­Cl showing selectivity for both MDR lines and [RhCp*­(DCP)­Cl]Cl being selective only to MES-SA/Dx5 cells. In contrast, significant resistance was observed when RuCym complexes were tested; thus, these compounds are presumably potent P-gp substrates with low or no activity to MDR cells, similar to the P-gp substrate etoposide, to which both MDR cells showed resistance (6-fold resistance of MES-SA/B1 and 10-fold of MES-SA/Dx5). Analogous half-sandwich complexes of PHEN were also tested in MES-SA and MES-SA/Dx5 monocultures, revealing a similar trend: the RuCym complex exhibited lower activity and reduced MDR-selectivity compared to the free ligand, while complexation with RhCp* preserved both activity and selectivity.^14^

Considering that BP elicited the highest cytotoxicity against every cell line and that [RhCp*­(BP)­Cl]Cl retained an MDR-selective toxicity, we chose this complex for the nanoformulation studies.

### Cytotoxicity and MDR Selectivity of the Nanoformulated Complex [RhCp*­(BP)­Cl]­Cl

Cytotoxicity of nanoformulated [RhCp*­(BP)­Cl]­Cl with cross-linked HSA and HTP carriers was tested against the previously introduced triple coculture of fluorescent MES-SA cells with a slight difference. Namely, the media containing the dissolved products were removed from the wells, and fresh media was added prior to the measurement as the presence of cross-linked HSA and HTP formulations interfered with the fluorescence of the cells. The cytotoxicity data determined for the complexes and its nanoformulated forms are shown in Figure 12 (and in Table 5).

**Figure 12:**
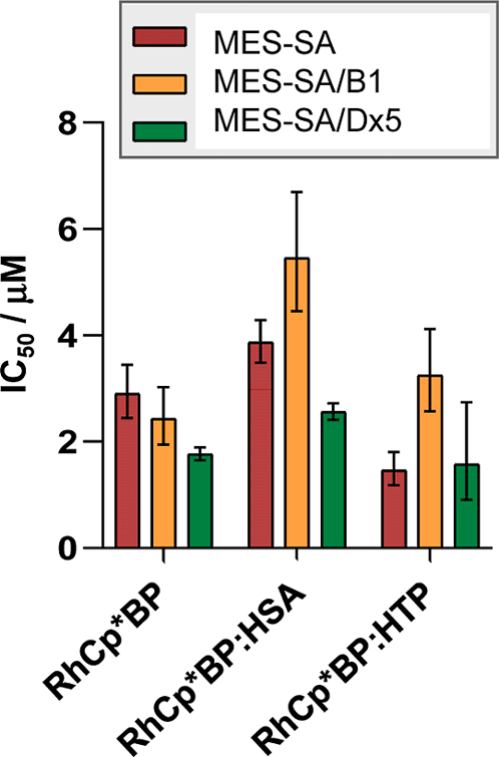
Cytotoxicity of [RhCp*­(BP)­Cl]­Cl and its cross-linked HSA and HTP nanoformulated forms against cocultured MES-SA mCherry, MES-SA/B1-mOrange, and MES-SA/Dx5-eGFP cells.

Cytotoxicity of the free [RhCp*­(BP)­Cl]Cl was slightly higher than its HSA-based formulation against all three cell lines. At the same time, the protein HSA itself was not toxic, as it was expected, while the cross-linked HSA carrier (without the complex) triggered cell death only at a higher concentration than in the HSA-based formulation (Figure S24).

The complex with the HTP formulation was 2-fold more cytotoxic against the parental MES–SA cell line but exhibited similar cytotoxicity against the resistant cells as the complex itself. It should be noted that the empty HTP alone was also similarly cytotoxic as the complex with the HTP-based formulation.

## Conclusions

In conclusion, this comprehensive study provides a detailed characterization of the RhCp* and RuCym complexes of two PHEN derivatives (BP and DCP) in both the solid and solution phases. It highlights their potential as effective agents against MDR cancer cells while enhancing their physicochemical and binding properties, which are highly relevant in early stage drug development.

The composition and purity of the complexes with the general formula [RuCym/RhCp*­(DCP/BP)­Cl]Cl were confirmed by NMR and ESI-MS methods. The IR spectra confirmed the coordination of the ligands and offered valuable insights into the distribution of the rings π-electron densities within the PHEN scaffold, indicating a more quinoid-like state. Crystal structures of complexes [RuCym­(BP)­Cl]­PF_6_ and [RhCp*­(DCP)­Cl)]­PF_6_ were determined using SC-XRD. The analysis revealed the typical piano-stool geometry, where the coordination sphere is completed by the bidentate ligand with an (N,N) chelating donor atom set and a chlorido coligand. The complexes exhibit high stability in the aqueous phase across a wide pH range. The p*K*_a_ and log*K*′ (H_2_O/Cl^–^) constants, determined by UV–vis spectrophotometry, were found to be higher for the RhCp* complexes, indicating their lower propensity for deprotonation at pH 7.4, and a stronger retention of the chlorido coligand within the coordination sphere. The distribution coefficients confirmed that the complexes show significantly enhanced hydrophilicity and aqueous solubility compared with their corresponding ligands.

The RhCp* complexes exhibit stronger binding to HSA than their RuCym analogues, with BP complexes showing affinity higher than that of DCP complexes. This enhanced interaction is beneficial, as binding to HSA can improve the pharmacokinetic properties of the complexes by prolonging circulation time and potentially facilitating passive tumor targeting via the EPR effect. The reduced level of binding of RuCym complexes is attributed to kinetic limitations. The CZE results suggested the binding of at least 2 equiv of the RhCp* complexes on HSA.

The complexes (and BP, DCP alone, and PHEN for comparison) were tested on ER-negative MDA-MB-231 and ER-positive MCF-7 and T-47D breast cancer cell lines. The ligands and their complexes displayed significant cytotoxicity (IC_50_ = 0.6–8.6 μM), except for RuCymDCP. Their activity was tested in vitro using a MDR model system consisting of a cocultured MES–SA uterine sarcoma cell line and its P-gp (ABCB1) overexpressing variants. BP was hypertoxic to both the MDR MES–SA/B1 and MES–SA/Dx5 cells, compared with the parental line, while DCP was more active only against MES–SA/Dx5. The RuCym complexes showed lower activity compared to their corresponding ligands, with low or no activity to MDR cells as found as potent P-gp substrates. Meanwhile, the RhCp* complexes show similar cytotoxic activity and MDR-selectivity to the ligands. The lead complex [RhCp*­(BP)­Cl]­Cl exhibited high cytotoxicity against every cell line tested and high MDR selective toxicity (SR = 1.1 and 6.7). Its cytotoxicity was comparable to that of cisplatin on the breast cancer cell lines, and it was more active against the MDR MES–SA/DX5 cells compared to cisplatin and etoposide, which do not display MDR-selectivity. Moreover, this complex showed good aqueous solubility and significant HSA binding affinity; thus, it was selected for nanoformulation studies. HSA, as a biocompatible protein-based carrier, was used alone and in combination with d-α-tocopheryl polyethylene glycol 1000 succinate to enhance stability of the complex and support targeted drug delivery. Both the glutaraldehyde cross-linked HSA and HTP carriers demonstrated potential as drug delivery systems for this complex with excellent encapsulation efficiency, high colloidal stability, and slow drug release. The nanoformulation with cross-linked HSA, however, slightly decreased the cytotoxicity against the tested uterine sarcoma cell line and retained the hypertoxicity to the MDR MES–SA/Dx5 cells, and this carrier did not induce intrinsic cytotoxicity at the applied concentrations, unlike the HTP carrier. Our results identify the complex [RhCp*­(BP)­Cl]Cl and its nanoformulated variant encapsulated in cross-linked HSA as promising candidates for further in vivo evaluation.

## Experimental Section

### Chemicals

All solvents were of analytical grade and used without further purification. [Rh­(η^6^-C_5_Me_5_)­(μ-Cl)­Cl]_2_, [Ru­(η^6^-*p*-cymene)­(μ-Cl)­Cl]_2_, BP, DCP, *n*-octanol, DMSO-*d*_6_, D_2_O, HSA (A8763, essentially globulin free), DG, 4,4-dimethyl-4-silapentane-1-sulfonic acid (DSS), MeIm, d-α-tocopherol succinate (T3126), poly­(d,l-lactide-*co*-glycolide)­(50:50)-*block*-poly­(ethylene glycol) (900664, PLGA–PEG), glutaraldehyde were Sigma-Aldrich products and were used without further purification. NaH_2_PO_4_, Na_2_HPO_4_, KH_2_PO_4_, KCl, KNO_3_, NaCl, HNO_3_, KOH, HCl, toluene, ethanol, acetone, diethyl-ether, CH_2_Cl_2_ were Molar Chemicals or VWR products.

### Stock Solutions and Sample Preparation

For the preparation of stock and sample solutions, Milli-Q water was used. Stock solutions of HSA were prepared in PBS’ containing 12 mM Na_2_HPO_4_, 3 mM KH_2_PO_4_, 1.5 mM KCl, and 100.5 mM NaCl, in which the concentration of the components (K^+^, Na^+^, and Cl^–^ ions) corresponds to the human blood serum. Stock solutions of metal complexes were prepared in PBS’ medium at pH 7.4 to ensure physiological relevance and maintain stability during investigations. For H_2_O/Cl^–^ exchange studies, a 20 mM phosphate buffer was employed, with the pH adjusted to 6.0 using potassium acid (HNO_3_). For formulation purposes, a stock solution of the [RhCp*­(BP)­Cl]­Cl complex was prepared in Milli-Q (MQ) water. Residual citric acid content of HSA was removed by repeated ultrafiltration of the protein stock solution, and its concentration was calculated from its UV absorption: λ_280 nm_ (HSA) = 36,850 M^–1^ cm^–1^.^65^

### Synthesis and Characterization of the Complexes

The synthesis of the complexes was carried out using a method similar to those previously reported.^14^ In this procedure, the precursor metal dimer [Ru­(η^6^-*p*-cymene)­Cl_2_]_2_ or [Rh­(η^5^-C_5_Me_5_)­Cl_2_]_2_ (1 equiv) was dissolved in CH_2_Cl_2_, followed by the addition of the ligand (2 equiv). The reaction mixture was stirred for 24 h. After the solvent was evaporated, the residue was treated with a small amount of diethyl ether to facilitate precipitation. The resulting solid complexes were then isolated by vacuum filtration, washed with diethyl ether, and dried in an oven at 45 °C for 4 h. This procedure was consistently applied to synthesize each complex. The reported yields are based on single synthetic preparations.

#### Synthesis of Ru­(II)­(η^6^-*p*-cymene)­(DCP)­Cl]­Cl [RuCym­(DCP)­Cl]­Cl

[Ru­(η^6^-*p*-cymene)­Cl_2_]_2_ (19.9 mg, 32.5 μmol) and DCP (16.2 mg, 65.0 μmol), Yield: 27.4 mg (76%). ESI-MS [RuCym­(DCP)­Cl]^+^ calc. *m*/*z*: 518.9736; found *m*/*z*: 518.9741. ^1^H NMR (CD_3_OD, δ/ppm, 500 MHz, Figure S25): 9.79 (d, *J* = 5.8 Hz, 2H, 2 and 9), 8.58 (s, 2H, 5 and 6), 8.31 (d, *J* = 5.8 Hz, 2H, 3 and 8), 6.25, 6.02 (d, d, *J* = 6.4 Hz, *J* = 6.4 Hz, 4H, C2 C6 & C3 C5), 2.71 (hept, J = 6.9, 1H, C7), 2.25 (s, 3H, C10), 1.05 (d, *J* = 6.9 Hz, 6H, C8 & C9). ^13^C NMR (CD_3_OD, δ/ppm, 125 MHz, Figure S26): 157.28 (2 and 9), 147.70 (10a and 10b), 147.24 (4 and 7), 130.58 (4a and 6a), 128.32 (5 and 6), 126.02 (3 and 8), 107.26 (C1), 104.95 (C4), 87.43 (C3 & C5), 85.70 (C2 & C6), 32.38 (C7), 22.30 (C8 & C9), 18.82 (C10).

#### Synthesis of [Rh­(III)­(η^5^-C_5_Me_5_)­(DCP)­Cl]Cl [RhCp*­(DCP)­Cl]­Cl

[Rh­(η^5^-C_5_Me_5_)­Cl_2_]_2_ (19.9 mg, 32.2 μmol) and DCP (16.1 mg, 64.4 μmol), yield: 30.0 mg (83%). ESI-MS [RhCp*­(DCP)­Cl]^+^ calc. *m*/*z*: 520.9825; found *m*/*z*: 520.9840. ^1^H NMR (CD_3_OD, δ/ppm, 500 MHz, Figure S27): 9.33 (d, *J* = 5.7 Hz, 2H, 2 and 9), 8.62 (s, 2H, 5 and 6), 8.37 (d, *J* = 5.7 Hz, 2H, 3 and 8), 1.81 (s, 15H, C5Me5). ^13^C NMR (CD_3_OD, δ/ppm, 125 MHz, Figure S28): 153.86 (2 and 9), 147.57 (10a and 10b), 147.29 (4 and 7), 130.57 (4a and 6a), 128.82 (5 and 6), 126.11 (3 and 8), 99.32, 99.26 (C5), 8.97 (Me5).

#### Synthesis of [Ru­(II)­(η^6^-*p*-cymene)­(BP)­Cl]­Cl [RuCym­(BP)­Cl]­Cl

[Ru­(η^6^-*p*-cymene)­Cl_2_]_2_ (20.6 mg, 33.6 μmol) and BP (22.4 mg, 67.2 μmol), Yield: 36.7 mg (85%). ESI-MS [RuCym­(BP)­Cl]^+^ calc. *m*/*z*: 603.1141; found *m*/*z*: 603.1134. ^1^H NMR (CD_3_OD, δ/ppm, 500 MHz, Figure S29): 9.88 (d, *J* = 5.5 Hz, 2H, 2 and 9), 8.15 (s, 2H, 5 and 6), 8.06 (d, *J* = 5.5 Hz, 2H, 3 and 8), 7.70–7.62 (m, 10H, Ph2 – Ph6 & Ph′2 – Ph′6), 6.28, 6.05 (d, d, *J* = 6.4, 6.3 Hz, 4H, C2 C6 & C3 C5), 2.76 (hept, *J* = 6.9 Hz, 1H, C7), 2.31 (s, 3H, C10), 1.08 (d, J = 6.9, 6H, C8 & C9). ^13^C NMR (CD_3_OD, δ/ppm, 125 MHz, Figure S30): 156.52 (2 and 9), 152.87 (4 and 7), 147.85 (10a and 10b), 136.67 (Ph1 & Ph′1), 131.07, 130.99, 130.38 (Ph2 – Ph6 & Ph′2–Ph′6), 129.97 (4a and 6a), 127.72 (5 and 6), 126.83 (3 and 8), 106.66 (C1), 104.80 (C4), 87.62 (C3 & C5), 85.82 (C2 & C6), 32.45 (C7), 22.33 (C8 & C9); 18.91 (C10).

#### Synthesis of [Rh­(III)­(η^5^-C_5_Me_5_)­(bathophenanthroline)­Cl]Cl [RhCp*­(BP)­Cl]­Cl

[Rh­(η^5^-C_5_Me_5_)­Cl_2_]_2_ (20.2 mg, 32.7 μmol) and BP (21.8 mg, 65.4 μmol), yield: 31.5 mg (75%). ESI-MS [RhCp*­(BP)­Cl]^+^ calc. *m*/*z*: 605.1231; found *m*/*z*: 605.1223. ^1^H NMR (CD_3_OD, δ/ppm, 500 MHz, Figure S31): 9.43 (d, *J* = 5.4 Hz, 2H, 2 and 9), 8.18 (s, 2H, 5 and 6), 8.15 (d, *J* = 5.4 Hz, 2H, 3 and 8), 7.71–7.63 (m, 10H, Ph2–Ph6 & Ph′2–Ph′6), 1.86 (s, 15H, C5Me5). ^13^C NMR (CD_3_OD, δ/ppm, 125 MHz, Figure S32): 153.23 (4 and 7), 153.07 (2 and 9), 147.43 (10a and 10b), 136.87 (Ph1 & Ph′1), 131.12, 130.92, 130.37 (Ph2–Ph6 & Ph′2–Ph′6), 130.02 (4a and 6a), 128.30 (5 and 6), 126.96 (3 and 8), 99.08, 99.02 (C5), 9.04 (Me5).

The ^1^H NMR spectra of the ligands (BP and DCP) are shown in Figures S33 and S34 for comparison.

### NMR Spectroscopy

A Bruker AVANCE III HD Ascend 500 Plus instrument was used for NMR studies. For the characterization of the complexes both the ^1^H and ^13^C NMR spectra were recorded in CD_3_OD (10 mM, *t* = 25 °C). The ^13^C NMR spectra were obtained with the attached proton test distinguishing CH and CH_3_ as positive peaks and quaternary carbons and CH_2_ as negative peaks. ^1^H NMR spectroscopic measurements of the complexes with MeIm were carried out employing a WATERGATE water suppression pulse scheme in the presence of 10% (*v*/*v*) D_2_O/90% (*v*/*v*) H_2_O. DSS internal standard was added to the samples to obtain reference peaks.

### IR Spectroscopy

The mid-IR spectra were recorded for the complexes between 4000 and 400 cm^–1^, at a 2 cm^–1^ resolution, on a Varian Scimitar 2000 FT-IR spectrometer equipped with a “Golden Gate” single reflection ATR unit (Specac Ltd.) with a diamond internal reflection element (IRE). The detector was a broadband mercury cadmium telluride liquid N_2_-cooled unit. The far-IR spectra were recorded between 700 and 50 cm^–1^, at 4 cm^–1^ resolution by a Bio-Rad Digilab FTS-60A spectrometer equipped with a “GladiATR” single reflection ATR unit (Pike Technologies) with IRE. The detector was a deuterated triglycine sulfate unit, equipped with a high-density polyethylene (HDPE) window.

Samples were placed and pressed onto IRE materials without any treatment. All spectra were corrected by the corresponding water vapor spectrum, and baseline and offset corrections took place where it was necessary. All far-IR spectra were zapped between 575 and 500 cm^–1^ since they did not contain valuable spectroscopic information due to the strong absorption of the HDPE-window and the Mylar beamsplitter. All spectral manipulations were performed by GRAMS/AI version 8.0 (Thermogalactic) spectroscopic software. For the interpretation of the IR spectra analyses reported for elated complexes were also used.^31–33,35^

### Electrospray Mass Spectrometry

A Waters Q-TOF Premier (Micromass MS Technologies) mass spectrometer with an electrospray ion source was used to perform high-resolution (HR) ESI–MS experiments. Samples contained 100 μM compounds in methanol (LC–MS grade).

### Crystallization, X-Ray Data Collection, Structure Solution, and Data Refinement

For crystallization, the complexes [RuCym­(BP)­Cl]­Cl and [RhCp*­(DCP)­Cl)]Cl were dissolved in CH_2_Cl_2_ followed by the addition of an equimolar amount of NH_4_PF_6_. After 48 h of stirring, the resulting NH_4_Cl was filtered off. The solution was partly evaporated and precipitation was obtained by the addition of diethyl ether, followed by filtration of the solid product. From acetone solution of [RuCym­(BP)­Cl]­PF_6_ and CH_2_Cl_2_ solution of [RhCp*­(DCP)­Cl)]­PF_6_, single crystals of [RuCym­(BP)­Cl]­PF_6_ × Et_2_O (**I**) and [RhCp*­(DCP)­Cl)]­PF_6_ (**II**) were obtained, respectively, with diethyl ether by the vapor diffusion method.

The yellow crystals (**I** as a chunk, **II** as a platelet) were mounted on a loop and transferred to the goniometer. X-ray diffraction data were collected at low temperature (153(2) K) and 113(2) K, respectively, on a Rigaku RAXIS–RAPID II diffractometer using Cu Kα radiation. Numerical absorption correction^66^ was carried out using the program RAPID–AUTO (Version 3.1.1.).^67^ Using Olex2,^68^ the structure was solved with the SHELXT^69^ structure solution program using Intrinsic Phasing and refined with the olex2.refine^70^ refinement package using Gauss–Newton minimization. Refinement of non-hydrogen atoms was carried out with anisotropic temperature factors. Hydrogen atoms were placed in geometric positions. They were included in structure factor calculations, but they were not refined. The isotropic displacement parameters of the hydrogen atoms were approximated from the *U*(equiv) value of the atom to which they were bonded. Constraints were used to calculate the thermal ellipsoids of *p*-cymene molecules due to greater displacement. The summary of data collection and refinement parameters is collected in Table S1. Graphical representation and the edition of CIF files were done by Mercury^40^ and enCIFer^71^ software. The crystallographic data files have been deposited with the Cambridge Crystallographic Database as CCDC 2422759–2422760.

### UV–Visible Spectrophotometry and Spectrofluorometry

An Agilent Cary 8454 diode array spectrophotometer was utilized to obtain UV–vis spectra in the wavelength range 190–1100 nm; the path length was 1 cm. The concentrations of the complexes were between 20 and 100 μM. The actual pH of the samples were measured with an Orion 710A pH-meter equipped with a Metrohm combined electrode (type 6.0234.100) and the pH titrations were performed by a KOH solution using a Metrohm 665 Dosimat buret at 25.0 °C. The electrode system was calibrated according to the method suggested by Irving et al.^72^ The average water ionization constant, p*K*_w_, was determined as 13.76 ± 0.01. The computer program HypSpec^55^ was used to calculate the equilibrium constants.

Fluorescence measurements were carried out on a Fluoromax (Horiba Jobin Yvon) spectrofluorometer using a 1 cm × 1 cm quartz cuvette in order to assess the interactions of the complexes with HSA in PBS’ medium at 25 °C. The conditions applied for the steady-state measurements are shown in Table S7. The calculated quenching or displacement conditional constants for the HSA-complex species were obtained using the computer program HypSpec.^55^ Calculations always were based on data obtained from at least two independent measurements. Self-absorbance and inner filter effect had to be taken into account,^73^ and corrections were made as it was described in our former works.^11,56^

### Determination of Distribution Coefficients

The traditional shake-flask method was used to obtain distribution coefficients of the ligands as well as of the complexes in *n*-octanol/buffered aqueous solution (20 mM phosphate, pH = 7.4) at different chloride ion concentrations (4, 24, and 100 mM) using UV–vis spectrophotometry (Agilent Cary 8454 diode array spectrophotometer) for the analysis. The compounds were dissolved in buffered aqueous solutions previously saturated with *n*-octanol. Then the aqueous and *n*-octanol phases were gently mixed in different volume ratios (using 1:60 for the ligands, 1:10 for the RhCp* and the RuCym complexes ratios of *n*-octanol to buffered aqueous solution volume) for 4 h, followed by phase separation. Then the UV–vis spectrum of the original aqueous sample and the aqueous and *n*-octanol phases was recorded and distribution coefficients (*D*) were calculated according to the formula described in our former work in detail.^74^

### Capillary Zone Electrophoresis

The interaction of complex [RhCp*­(BP)­Cl]Cl (ca. 100 μM) with HSA (ca. 50 μM) in PBS’ medium was studied by an Agilent 7100 capillary electrophoresis system equipped with a diode-array UV–vis detector (200–600 nm). Fused silica capillaries of 48 cm total length with a 75 μm inner diameter were used (Agilent Technologies). The background electrolyte (BGE) was PBS’ buffer (pH = 7.4), in which the samples were also made. The conditioning process of new capillaries and daily preparation were performed as described formerly.^55^ In order to ensure a steady baseline, the capillary was flushed with BGE (2 min) before each run and was rinsed with NaOH (0.1 M; 1.5 min), H_2_O (1.5 min), and then with BGE (2 min) after each run. The capillary cassette was kept at 25 °C. The hydrodynamic injection was used at 50 mbar for 5 s injection time. For separation, an 8 kV voltage (with *ca*. 100 μA current) was applied. The sample run time was set to 18 min. The computer program ChemStation (Agilent) was used to record electropherograms.

### HSA-Based Formulations

HSA-based formulations were prepared by dissolving 20 mg of HSA in 1 mL of the desired concentration of the complex compound. The mixture was stirred at 150 rpm for 1 h. Subsequently, in order to precipitate HSA, 8 mL of absolute ethanol was added dropwise to the system at a flow rate of 0.8 mL/min under gentle stirring. The mixture was then stirred at 250 rpm for 30 min. To cross-link the formulation, 18 μL of 8% (m/m) glutaraldehyde aqueous solution was added under stirring, and the reaction mixture was stirred at 250 rpm for 5 h. After the reaction, the formulation was centrifuged at 10,000 rpm for 30 min at 25 °C. The supernatant was removed and stored for further analysis, while the pellet was redispersed in 10 mL of MQ water. Redispersion was aided by using an ultrasonic bath operating at 27 kHz for 5 min. Control formulations (empty carriers) were prepared following the same procedure without the addition of the metal complex.

### HTP-Based Formulations

HSA–TPGS–PLGA formulations were prepared by using stock solutions of 20 mg/mL HSA in MQ water, 20 mg/mL PLGA in acetone, and 10 mg/mL TPGS in MQ water. The formulation was prepared with a mass ratio of HSA:PLGA:TPGS as 1:1:0.5. Initially, 1 mL of HSA stock solution was mixed with 1 mL of the desired concentration of the complex compound and stirred at 150 rpm for 1 h. Following this, 13 mL of MQ water was added to the system and 1 mL of TPGS stock solution was introduced at 320 rpm. The mixture was stirred at 120 rpm for 1 h. Subsequently, 1 mL of PLGA stock solution was added dropwise to the system at 720 rpm, followed by stirring at 320 rpm for another hour. The resulting formulation was centrifuged at 10,000 rpm for 30 min at 25 °C. The supernatant was collected for further analysis, and the pellet was redispersed in 10 mL of MQ water. Redispersion was aided using an ultrasonic bath operating at 27 kHz for 5 min. Control formulations (empty carriers) were also prepared. In the development of our colloidal drug carrier systems, we can confidently assert that no free components are present alongside the formulated carriers. This assurance is based on the rigorous purification process, particularly centrifugation, which effectively removes free active substances from the final formulation. The reliability of this approach has been validated by our research group, with supporting evidence from IR and differential scanning calorimetry measurements.^75^

### Characterization of the HSA-Based and HTP Formulations

For the characterization of the cross-linked HSA and HTP particles, the average size, size distribution, and zeta potential (ζ-potential) values were measured by a HORIBA SZ-100 NanoParticle Analyzer equipment. For each sample (after 10-fold dilution), measurements were performed in triplicate, and for each sample, 10 parallel data points were recorded. The light source was a semiconductor laser (λ = 532 nm, 10 mW), and photomultiplier tubes were used as a detector at a 90° scattering angle. For calculation of the zeta potential values, the Smoluchowski equation was used by converting the measured electrophoretic mobility data. For the TEM studies, a Jeol JEM-1400plus equipment at a 120 keV accelerating voltage was applied.

### Encapsulation Efficiency Determination

The encapsulation efficiency of the nanoformulation was determined by analyzing the supernatant, which contains the residual nonencapsulated complex, using UV–vis spectroscopy. The concentration of the complex in the supernatant was calculated using an external calibration curve prepared in a medium of 8:1 absolute ethanol/MQ water (HSA-based formulation) or 1:16 acetone/MQ water (HTP-formulation). The encapsulated amount of the complex was calculated as the difference between the total amount initially introduced into the formulation and the amount remaining in the supernatant. The EE % was then calculated using the formula

$$EE\;\%=\left(\frac{n_{encapsulated}}{n_{total}}\right)\times 100=\left(\frac{n_{total}-n_{supernatant}}{n_{total}}\right)\times 100$$

where *n*_encapsulated_ is the amount of complex encapsulated in the formulation, *n*_total_ is the total amount of complex initially added, and *n*_supernatant_ is the amount of complex remaining in the supernatant. This methodology ensures an accurate determination of encapsulation efficiency for both types of formulations.

### Release Studies

The release experiments were conducted in PBS’ medium using modified rapid equilibrium dialysis (RED) inserts of Thermo Scientific. Briefly, the receiver compartment of the RED insert was removed in order to fit the dialysis bag into a regular 1 cm quartz cuvette (see Figure S19 for the layout). The dialysis bag (donor compartment) contained 300 μL of aqueous solution of the formulation or the metal complex alone (*c* = 60 μM) or the HSA or HTP-based formulations. The cuvette was the acceptor/receiver compartment containing PBS’ buffer (2.0 mL). The whole device was capped with the parafilm and the buffer in the cuvette was continuously stirred; no stirring was applied in the RED insert. The release of the complex into the buffer was monitored online using an Agilent Cary 3500 eight-channel photometer. All samples were done in duplicate.

### In Vitro Cell Studies: Cell Lines, Culture Conditions, and Cytotoxicity Assay

MDA-MB-231 breast adenocarcinoma and MCF-7 and T-47D invasive breast carcinomas were purchased from the Developmental Therapeutic Program of the National Cancer Institute (NIH, USA), and they were cultured in RPMI medium (Thermo Fisher). MES–SA and MES–SA/Dx5 uterine sarcoma cell lines were purchased from American Type Culture Collection and were kept in DMEM (Thermo Fisher). MES–SA/B1 was created by the transfection of the human MDR1 gene in MES–SA, and MES–SA mCherry, MES–SA/B1 mOrange, and MES–SA/Dx5 eGFP were engineered to stably express the respective fluorescent proteins, as described previously.^75^ Culture media were supplemented with 10% fetal bovine serum, 2 mM l-glutamine, 100 units/mL penicillin, and 100 μg/mL streptomycin (Thermo Fisher).^76^ Cells were kept at 37 °C under 5% CO_2_ and were negative for mycoplasma infection.

Cytotoxicity of the nonfluorescent cancer cells (MDA-MB-231, MCF-7, T-47D) were assessed with PrestoBlue cell viability reagent (Thermo Fisher). Briefly, 2500 cells/well were seeded on 384-well plates, and then the next day drugs were added. After 72 h incubation, PrestoBlue was added in a 10% final concentration, and after an hour of additional incubation, plates were measured with a PerkinElmer EnSpire plate reader at 555 nm/585 nm excitation/emission wavelengths. Fluorescent cells (MES-SA mCherry, MES-SA/B1 mOrange, MES-SA/Dx5 eGFP) were admixed before seeding on 384-well plates at 800 cells/well density each, thus altogether 2400 cells/well. Drugs were added the following day, and the intensity of the fluorescent proteins were measured after 144 h incubation at the respective fluorescent channels (excitation/emission: eGFP: 485 nm/510 nm; mCherry: 585 nm/610 nm; mOrange: 545 nm/567 nm). pIC_50_ values were calculated by a custom program written by Judit Sessler in C#. IC_50_ values and standard deviation were calculated from average pIC_50_ values from at least 3 independent measurements. Since the IC_50_ values were obtained through nonlinear regression fitting of the viability vs. log­(concentration) data, the resulting confidence intervals are inherently asymmetric (see pIC_50_ values and their corresponding SD in Tables S5 and S6).

## Supplementary Material



## Notes

Interpretation of the IR spectra; SC-XRD data; time-dependent UV–vis spectra of the complexes in PBS’ buffer; interaction with HSA: UV–vis spectra, electropherograms; interaction with HSA: fluorescence and 1H NMR spectra; modified RED setup; cytotoxicity data; NMR spectra for characterization; and conditions applied for steady-state spectrofluorometric measurements (PDF)

## Abbreviations

APT: attached proton test
BGE: background electrolyte
BP: 4,7-diphenyl-1,10-phenanthroline, bathophenanthroline
BPY: 2,2′-bipyridine
CSD: Cambridge structural database
CZE: capillary zone electrophoresis
DG: dansylglycine
DCP: 4,7-dichloro-1,10-phenanthroline
DLS: dynamic light scattering
DSS: sodium trimethylsilylpropanesulfonate
EE %: encapsulation efficiency
EMEM: Eagle’s minimum essential medium
EPR: enhanced permeability and retention
ESI–MS: electrospray ionization mass spectrometry
HDPE: high density polyethylene
HSA: human serum albumin
HTP: HSA–TPGS–PLGA
*K*_a_: proton dissociation constant
*K*_w_: water ionization constant
MeIm: 1-methylimidazole
MDR: multidrug resistance
PBS’: phosphate-buffered saline
PHEN: 1,10-phenanthroline
P-gp: P-glycoprotein or ABCB1
PLGA: poly­(lactic-*co*-glycolic acid)
RhCp*: Rh­(η^5^-C_5_Me_5_)
RhCp*BP: [Rh­(III)­(η^5^-C_5_Me_5_)­(bathophenanthroline)­Cl]­Cl
RhCp*DCP: [Rh­(III)­(η^5^-C_5_Me_5_)­(4,7-dichloro-1,10-phenanthroline)­Cl]­Cl
RuCym: Ru­(η^6^-*p*-cymene)
RuCymBP: [Ru­(II)­(η^6^-*p*-cymene)­(bathophenanthroline)­Cl]­Cl
RuCymDCP: [Ru­(II)­(η^6^-*p*-cymene)­(4,7-dichloro-1,10-phenanthroline)­Cl]­Cl
SC-XRD: single crystal X-ray diffraction
SR: selectivity ratio
TEM: transmission electron microscopy
TPGS: d-α-tocopheryl polyethylene glycol 1000 succinate
UV–vis: UV–visible
ζ-potential: electrokinetic potential

## References

[1] Dilruba S., Kalayda G. V. (2016). Platinum-Based Drugs: Past, Present and Future. Cancer Chemother. Pharmacol..

[2] Szakács G., Paterson J. K., Ludwig J. A., Booth-Genthe C., Gottesman M. M. (2006). Targeting Multidrug Resistance in Cancer. Nat. Rev. Drug Discovery.

[3] Valente A., Podolski-Renić A., Poetsch I., Filipović N., López Ó., Turel I., Heffeter P. (2021). Metal- and Metalloid-Based
Compounds to Target and Reverse Cancer Multidrug Resistance. Drug Resistance Updates.

[4] Türk D., Hall M. D., Chu B. F., Ludwig J. A., Fales H. M., Gottesman M. M., Szakács G. (2009). Identification of Compounds Selectively
Killing Multidrug-Resistant Cancer Cells. Cancer
Res..

[5] Pape V. F. S., Gaál A., Szatmári I., Kucsma N., Szoboszlai N., Streli C., Fülöp F., Enyedy É. A., Szakács G. (2021). Relation of Metal-Binding Property and Selective Toxicity
of 8-Hydroxyquinoline Derived Mannich Bases Targeting Multidrug Resistant
Cancer Cells. Cancers.

[6] Cserepes M., Türk D., Tóth S., Pape V. F. S., Gaál A., Gera M., Szabó J. E., Kucsma N., Várady G., Vértessy B. G., Streli C., Szabó P. T., Tovari J., Szoboszlai N., Szakács G. (2020). Unshielding
Multidrug Resistant Cancer through Selective Iron Depletion of P-Glycoprotein-Expressing
Cells. Cancer Res..

[7] Dömötör O., Pape V. F. S., May N. V., Szakács G., Enyedy É. A. (2017). Comparative Solution Equilibrium Studies of Antitumor
Ruthenium­(Η6-p-Cymene) and Rhodium­(Η5-C5Me5) Complexes
of 8-Hydroxyquinolines. Dalton Trans..

[8] Pivarcsik T., Tóth S., Pósa S. P., May N. V., Kováts É., Spengler G., Kántor I., Rolya A., Feczkó T., Szatmári I., Szakács G., Enyedy É. A. (2024). Organometallic
Half-Sandwich Complexes of 8-Hydroxyquinoline-Derived Mannich Bases
with Enhanced Solubility: Targeting Multidrug Resistant Cancer. Inorg. Chem..

[9] Pivarcsik T., Dömötör O., Mészáros J. P., May N. V., Spengler G., Csuvik O., Szatmári I., Enyedy É. A. (2021). 8-Hydroxyquinoline-Amino
Acid Hybrids and Their Half-Sandwich
Rh and Ru Complexes: Synthesis, Anticancer Activities, Solution Chemistry
and Interaction with Biomolecules. Int. J. Mol.
Sci..

[10] Pivarcsik T., Pósa V., Kovács H., May N. V., Spengler G., Pósa S. P., Tóth S., Nezafat Yazdi Z., Özvegy-Laczka C., Ugrai I., Szatmári I., Szakács G., Enyedy É. A. (2022). Metal Complexes of a 5-Nitro-8-Hydroxyquinoline-Proline
Hybrid with Enhanced Water Solubility Targeting Multidrug Resistant
Cancer Cells. Int. J. Mol. Sci..

[11] Dömötör O., Pivarcsik T., Mészáros J. P., Szatmári I., Fülöp F., Enyedy É. A. (2021). Critical Factors Affecting the Albumin
Binding of Half-Sandwich Ru­(II) and Rh­(III) Complexes of 8-Hydroxyquinolines
and Oligopyridines. Dalton Trans..

[12] Tao H., Wang R., Sheng W., Zhen Y. (2021). The Development of
Human Serum Albumin-Based Drugs and Relevant Fusion Proteins for Cancer
Therapy. Int. J. Biol. Macromol..

[13] Rahimizadeh P., Yang S., Lim S. I. (2020). Albumin:
An Emerging Opportunity
in Drug Delivery. Biotechnol. Bioprocess Eng..

[14] Mészáros J. P., Pape V. F. S., Szakács G., Németi G., Dénes M., Holczbauer T., May N. V., Enyedy É. A. (2021). Half-Sandwich Organometallic Ru and Rh Complexes of (N,N) Donor Compounds:
Effect of Ligand Methylation on Solution Speciation and Anticancer
Activity. Dalton Trans..

[15] Štarha P. (2025). Anticancer
Iridium­(III) Cyclopentadienyl Complexes. Inorg.
Chem. Front..

[16] Kostova I. (2025). Cytotoxic
Organometallic Iridium­(III) Complexes. Molecules.

[17] Liu Z., Sadler P. J. (2014). Organoiridium Complexes:
Anticancer Agents and Catalysts. Acc. Chem.
Res..

[18] Hearn J. M., Romero-Canelón I., Qamar B., Liu Z., Hands-Portman I., Sadler P. J. (2013). Organometallic Iridium­(III) Anticancer
Complexes with New Mechanisms of Action: NCI-60 Screening, Mitochondrial
Targeting, and Apoptosis. ACS Chem. Biol..

[19] Pracharova J., Novohradsky V., Kostrhunova H., Štarha P., Trávníček Z., Kasparkova J., Brabec V. (2018). Half-Sandwich Os­(Ii) and Ru­(Ii) Bathophenanthroline
Complexes: Anticancer Drug Candidates with Unusual Potency and a Cellular
Activity Profile in Highly Invasive Triple-Negative Breast Cancer
Cells. Dalton Trans..

[20] Fernández C. Y., Alvarez N., Rocha A., Ellena J., Costa-Filho A. J., Batista A. A., Facchin G. (2023). New Copper­(II)-L-Dipeptide-Bathophenanthroline
Complexes as Potential Anticancer Agents—Synthesis, Characterization
and Cytotoxicity Studies—And Comparative DNA-Binding Study
of Related Phen Complexes. Molecules.

[21] Golubeva Y. A., Lider E. V. (2024). Copper­(Ii) Complexes
Based on 2,2’-Bipyridine
and 1,10-Phenanthroline as Potential Objects for Developing Antitumor
Drugs. J. Struct. Chem..

[22] Figueroa-Depaz Y., Pérez-Villanueva J., Soria-Arteche O., Martínez-Otero D., Gómez-Vidales V., Ortiz-Frade L., Ruiz-Azuara L. (2022). Casiopeinas of Third Generations:
Synthesis, Characterization, Cytotoxic Activity and Structure–Activity
Relationships of Mixed Chelate Compounds with Bioactive Secondary
Ligands. Molecules.

[23] Masuri S., Vaňhara P., Cabiddu M. G., Moráň L., Havel J., Cadoni E., Pivetta T. (2022). Copper­(Ii) Phenanthroline-Based
Complexes as Potential Anticancer Drugs: A Walkthrough on the Mechanisms
of Action. Molecules.

[24] Gadre S., Manikandan M., Chakraborty G., Rayrikar A., Paul S., Patra C., Patra M. (2023). Development
of a Highly In Vivo Efficacious
Dual Antitumor and Antiangiogenic Organoiridium Complex as a Potential
Anti-Lung Cancer Agent. J. Med. Chem..

[25] Fernández C. Y., Alvarez N., Rocha A., Ellena J., Costa-Filho A. J., Batista A. A., Facchin G. (2023). New Copper­(II)-L-Dipeptide-Bathophenanthroline
Complexes as Potential Anticancer Agents—Synthesis, Characterization
and Cytotoxicity Studies—And Comparative DNA-Binding Study
of Related Phen Complexes. Molecules.

[26] Golubeva Y. A., Lider E. V. (2024). Copper­(Ii) Complexes
Based on 2,2’-Bipyridine
and 1,10-Phenanthroline as Potential Objects for Developing Antitumor
Drugs. J. Struct. Chem..

[27] Figueroa-Depaz Y., Pérez-Villanueva J., Soria-Arteche O., Martínez-Otero D., Gómez-Vidales V., Ortiz-Frade L., Ruiz-Azuara L. (2022). Casiopeinas of Third Generations:
Synthesis, Characterization, Cytotoxic Activity and Structure–Activity
Relationships of Mixed Chelate Compounds with Bioactive Secondary
Ligands. Molecules.

[28] Masuri S., Vaňhara P., Cabiddu M. G., Moráň L., Havel J., Cadoni E., Pivetta T. (2022). Copper­(Ii) Phenanthroline-Based
Complexes as Potential Anticancer Drugs: A Walkthrough on the Mechanisms
of Action. Molecules.

[29] Youk H. J., Lee E., Choi M. K., Lee Y. J., Jun H. C., Kim S. H., Lee C. H., Lim S. J. (2005). Enhanced
Anticancer Efficacy of α-Tocopheryl
Succinate by Conjugation with Polyethylene Glycol. J. Controlled Release.

[30] Muddineti O. S., Omri A. (2022). Current Trends in PLGA Based Long-Acting
Injectable Products: The
Industry Perspective. Expert Opin. Drug. Delivery.

[31] Colthup, N. Introduction to Infrared and Raman Spectroscopy; Elsevier, 2012.

[32] Bencze É., Lokshin B. V., Mink J., Herrmann W. A., Kühn F. E. (2001). Vibrational
Spectra and Structure of the Cyclopentadienyl-Anion (Cp−),
the Pentamethylcyclopentadienyl-Anion (Cp*−) and of Alkali
Metal Cyclopentadienyls CpM and Cp*M (M = Li, Na, K). J. Organomet. Chem..

[33] Larkin, P. J. IR and Raman Spectroscopy, Principles and Spectral Interpretation; Elsevier: Oxford, 2011; pp 92–93.

[34] Nakamoto, K. Complexes of Alcohols, Ethers, Ketones, Aldehydes, Esters and Carboxylic Acids. Complexes of Amino Acids. In Infrared and Raman Spectra of Inorganic and Coordination Compounds, Applications in Coordination, Organometallic, and Bioinorganic Chemistry; John Wiley & Sons, 2009; pp 231–233.

[35] Amberger H. D., Reddmann H. (2010). Electronic Structures of Organometallic Complexes of
f Elements LXXIV: First Raman Spectroscopic Polarization Measurements
on Uniformly Oriented Sandwich Complex Molecules: Bis­(Η5-Pentamethylcyclopentadienyl)
Ruthenium. J. Organomet. Chem..

[36] Hagebaum-Reignier D., Girardi R., Carissan Y., Humbel S. (2007). Hückel Theory
for Lewis Structures: Hückel-Lewis Configuration Interaction
(HL-CI). J. Mol. Struct.: THEOCHEM.

[37] Carissan Y., Hagebaum-Reignier D., Goudard N., Humbel S. (2008). Hückel-Lewis
Projection Method: A “Weights Watcher” for Mesomeric
Structures. J. Phys. Chem. A.

[38] Colina-Vegas L., Villarreal W., Navarro M., De Oliveira C. R., Graminha A. E., Maia P. I. D. S., Deflon V. M., Ferreira A. G., Cominetti M. R., Batista A. A. (2015). Cytotoxicity of Ru­(II) Piano-Stool
Complexes with Chloroquine and Chelating Ligands against Breast and
Lung Tumor Cells: Interactions with DNA and BSA. J. Inorg. Biochem..

[39] Graf M., Ochs J., Metzler-Nolte N., Böttcher H. C., Mayer P. (2023). Cytotoxic Activities of Half-Sandwich
M­(III) Complexes (M = Rh, Ir)
Bearing Chloro-Substituted Bidentate-Coordinated Phenanthroline or
Terpyridine Ligands. Z. Anorg. Allg. Chem..

[40] Macrae C. F., Edgington P. R., McCabe P., Pidcock E., Shields G. P., Taylor R., Towler M., Van De Streek J. (2006). Mercury: Visualization
and Analysis of Crystal Structures. J. Appl.
Crystallogr..

[41] Mészáros J. P., Dömötör O., Hackl C. M., Roller A., Keppler B. K., Kandioller W., Enyedy É. A. (2018). Structural
and Solution Equilibrium Studies on Half-Sandwich Organorhodium Complexes
of (N,N) Donor Bidentate Ligands. New J. Chem..

[42] Savić A., Gligorijević N., Arand̵elović S., Dojčinović B., Kaczmarek A. M., Radulović S., Van Deun R., Van Hecke K. (2020). Antitumor
Activity of Organoruthenium Complexes with Chelate Aromatic Ligands,
Derived from 1,10-Phenantroline: Synthesis and Biological Activity. J. Inorg. Biochem..

[43] Anuja P. K., Paira P. (2020). Luminescent Anticancer
Ru­(II)-Arenebipyridine and Phenanthroline
Complexes: Synthesis, Characterization, DFT Studies, Biological Interactions
and Cellular Imaging Application. J. Inorg.
Biochem..

[44] Youinou M. T., Ziessel R. (1989). Synthesis and Molecular
Structure of a New Family of
Iridium-(III) and Rhodium­(III) Complexes. J.
Organomet. Chem..

[45] Scharwitz M. A., Ott I., Geldmacher Y., Gust R., Sheldrick W. S. (2008). Cytotoxic
Half-Sandwich Rhodium­(III) Complexes: Polypyridyl Ligand Influence
on Their DNA Binding Properties and Cellular Uptake. J. Organomet. Chem..

[46] Blakemore J. D., Hernandez E. S., Sattler W., Hunter B. M., Henling L. M., Brunschwig B. S., Gray H. B. (2014). Pentamethylcyclopentadienyl Rhodium
Complexes. Polyhedron.

[47] Peacock A. F. A., Habtemariam A., Moggach S. A., Prescimone A., Parsons S., Sadler P. J. (2007). Chloro Half-Sandwich Osmium­(II) Complexes:
Influence of Chelated N,N-Ligands on Hydrolysis, Guanine Binding,
and Cytotoxicity. Inorg. Chem..

[48] Ariyoshi K., Kotera M., Namioka A., Suzuki T. (2020). A Specific Formation
of an Iridium­(III) Hydrido Complex Bearing 8-(Diphenylphosphino)­Quinoline. Polyhedron.

[49] Scharwitz M., Schäfer S., Van Almsick T., Sheldrick W. S. (2007). Chlorido­(5-Penta-Methyl-Cyclo-Penta-Dien-Yl)­(1,10-
Phenanthroline-2 N,N′)­Iridium­(III) Trifluoro-Methane-Sulfonate. Acta Crystallogr., Sect. E: Struct. Rep. Online.

[50] Enyedy É. A., Mészáros J. P., Dömötör O., Hackl C. M., Roller A., Keppler B. K., Kandioller W. (2015). Comparative
Solution Equilibrium Studies on Pentamethylcyclopentadienyl Rhodium
Complexes of 2,2’-Bipyridine and Ethylenediamine and Their
Interaction with Human Serum Albumin. J. Inorg.
Biochem..

[51] Kiss T., Enyedy É. A., Jakusch T., Dömötör O. (2019). Speciation
of Metal Complexes of Medicinal Interest: Relationship between Solution
Equilibria and Pharmaceutical Properties. Curr.
Med. Chem..

[52] Tiruppathi C., Song W., Bergenfeldt M., Sass P., Malik A. B. (1997). Gp60 Activation
Mediates Albumin Transcytosis in Endothelial Cells by Tyrosine Kinase-Dependent
Pathway. J. Biol. Chem..

[53] Elsadek B., Kratz F. (2012). Impact of Albumin on Drug Delivery
- New Applications on the Horizon. J. Controlled
Release.

[54] Belej D., Jurasekova Z., Nemergut M., Wagnieres G., Jancura D., Huntosova V. (2017). Negligible
Interaction of [Ru­(Phen)­3]­2+
with Human Serum Albumin Makes It Promising for a Reliable in Vivo
Assessment of the Tissue Oxygenation. J. Inorg.
Biochem..

[55] Gans P., Sabatini A., Vacca A. (1996). Investigation
of Equilibria in Solution.
Determination of Equilibrium Constants with the HYPERQUAD Suite of
Programs. Talanta.

[56] Dömötör O., Enyedy É. A. (2019). Binding Mechanisms of Half-Sandwich Rh­(III) and Ru­(II)
Arene Complexes on Human Serum Albumin: A Comparative Study. J. Biol. Inorg. Chem..

[57] Getreuer P., Marretta L., Toyoglu E., Dömötör O., Hejl M., Prado-Roller A., Cseh K., Legin A. A., Jakupec M. A., Barone G., Terenzi A., Keppler B. K., Kandioller W. (2024). Investigating the Anticancer Potential of 4-Phenylthiazole
Derived Ru­(Ii) and Os­(Ii) Metalacycles. Dalton
Trans..

[58] Patra J. K., Das G., Fraceto L. F., Campos E. V. R., Rodriguez-Torres M. D.
P., Acosta-Torres L. S., Diaz-Torres L. A., Grillo R., Swamy M. K., Sharma S., Habtemariam S., Shin H. S. (2018). Nano Based Drug Delivery Systems:
Recent Developments
and Future Prospects. J. Nanobiotechnol..

[59] Li C., Zhang D., Pan Y., Chen B. (2023). Human Serum Albumin
Based Nanodrug Delivery Systems: Recent Advances and Future Perspective. Polymers.

[60] Sabbagh F., Kim B. S. (2022). Recent Advances
in Polymeric Transdermal Drug Delivery
Systems. J. Controlled Release.

[61] Xie P., Jin Q., Zhang L., Zhang H., Montesdeoca N., Karges J., Xiao H., Mao X., Song H., Shang K. (2024). Endowing Pt­(IV) with Perfluorocarbon
Chains and Human Serum Albumin
Encapsulation for Highly Effective Antitumor Chemoimmunotherapy. ACS Nano.

[62] Zhang H., Montesdeoca N., Tang D., Liang G., Cui M., Xu C., Servos L. M., Bing T., Papadopoulos Z., Shen M., Xiao H., Yu Y., Karges J. (2024). Tumor-Targeted
Glutathione Oxidation Catalysis with Ruthenium Nanoreactors against
Hypoxic Osteosarcoma. Nat. Commun..

[63] Neophytou C. M., Constantinou C., Papageorgis P., Constantinou A. I. (2014). D-Alpha-Tocopheryl
Polyethylene Glycol Succinate (TPGS) Induces Cell Cycle Arrest and
Apoptosis Selectively in Survivin-Overexpressing Breast Cancer Cells. Biochem. Pharmacol..

[64] Atta-ur-Rahman; Choudhary, I. M. Frontiers in Drug Design & Discovery; Google Books, 2018; Vol. 9.

[65] Beaven G.
H., Chen S.-H., D’albis A., Gratzer W. B. (1974). A Spectroscopic
Study of the Haemin–Human-Serum-Albumin System. Eur. J. Biochem..

[66] Higashi, T. Numerical Absorption Correction; NUMABS, 2002.

[67] CrystalClear SM 1.4.0; Rigaku/MSC Inc., 2008.

[68] Dolomanov O. V., Bourhis L. J., Gildea R. J., Howard J. A. K., Puschmann H. (2009). OLEX2: A Complete
Structure Solution, Refinement and Analysis Program. J. Appl. Crystallogr..

[69] Sheldrick G. M. (2015). SHELXT—Integrated
Space-Group and Crystal-Structure Determination. Acta Crystallogr. A.

[70] Bourhis L. J., Dolomanov O. V., Gildea R. J., Howard J. A. K., Puschmann H. (2015). The Anatomy
of a Comprehensive Constrained, Restrained Refinement Program for
the Modern Computing Environment - Olex2 Dissected. Acta Crystallogr. A.

[71] Westrip S. P. (2010). PublCIF
Software for Editing, Validating and Formatting Crystallographic Information
Files. J. Appl. Crystallogr..

[72] Irving H. M., Miles M. G., Pettit L. D. (1967). A Study
of Some Problems in Determining
the Stoicheiometric Proton Dissociation Constants of Complexes by
Potentiometric Titrations Using a Glass Electrode. Anal. Chim. Acta.

[73] Lakowicz J. R. (2006). Instrumentation
for Fluorescence Spectroscopy. Princ. Fluoresc.
Spectrosc..

[74] Żołek T., Dömötör O., Ostrowska K., Enyedy É. A., Maciejewska D. (2019). Evaluation
of Blood-Brain Barrier
Penetration and Examination of Binding to Human Serum Albumin of 7-O-Arylpiperazinylcoumarins
as Potential Antipsychotic Agents. Bioorg. Chem..

[75] Kovács A. N., Varga N., Bogner J., Juhász Á., Csapó E. (2024). Enhancing the Hydrophilicity of Poly­(Lactic-Co-Glycolic
Acid) with Serum Albumin by Creating Colloidal Drug Carriers. J. Mol. Liq..

[76] Windt T., Tóth S., Patik I., Sessler J., Kucsma N., Szepesi Á., Zdrazil B., Özvegy-Laczka C., Szakács G. (2019). Identification
of Anticancer OATP2B1 Substrates by
an in Vitro Triplefluorescence-Based Cytotoxicity Screen. Arch. Toxicol..

