# Supplementary material for: Organometallic Half-Sandwich Complexes of 1,10-Phenanthroline Derivatives with Improved Solubility, Albumin-Binding, and Nanoformulation Potential Targeting Drug Resistance in Cancer
Source: Inorg Chem. 2025 Jul 11;64(29):14914–32. doi: 10.1021/acs.inorgchem.5c01556 (PMC12308784; doi:10.1021/acs.inorgchem.5c01556)
Supplement: Supplementary file 1 [file ic5c01556_si_001.pdf]

Supplementary Information

Organometallic half-sandwich complexes of 1,10-phenanthroline derivatives with improved solubility, albumin-binding, and nanoformulation potential targeting drug resistance in cancer

Egon F. Várkonyi,[a,b,c] *Szilárd Tóth,*[d,e,*] *Tamás Pivarcsik,*[a] *Orsolya Dömötör,*[a] *Ottó Berkesi,*[c] *Nóra V. May,*[f] *Gergely Szakács,*[d,g] *Edit Csapó,*[b,c,*] *Éva A. Enyedy*[a*]

[a] Department of Molecular and Analytical Chemistry, Interdisciplinary Excellence Centre, University of Szeged, Dóm tér 7-8, H-6720 Szeged, Hungary

[b] MTA-SZTE Lendület "Momentum" Noble Metal Nanostructures Research Group, University of Szeged, Rerrich B. sqr. 1, H-6720 Szeged, Hungary

[c] Department of Physical Chemistry and Materials Science, Interdisciplinary Excellence Centre, University of Szeged, Rerrich B. sqr. 1, H-6720 Szeged, Hungary

[d] Drug Resistance Research Group, Institute of Molecular Life Sciences, HUN-REN Research Centre for Natural Sciences, Magyar Tudósok krt. 2, H-1117 Budapest, Hungary

[e] National Laboratory for Drug Research and Development, Magyar Tudósok krt. 2, H-1117 Budapest, Hungary

[f] Centre for Structural Science, HUN-REN Research Centre for Natural Sciences, Magyar Tudósok krt. 2, H-1117 Budapest, Hungary

[g] Center for Cancer Research, Medical University of Vienna, Borschkegasse 8a, A-1090 Vienna, Austria

---

Table of contents

Interpretation of the IR spectra SI-2

SC-XRD data SI-11

Time-dependent UV-Vis and ^{1}H NMR spectra of the complexes in PBS' buffer SI-21

Interaction with HSA: UV-Vis spectra, electropherograms SI-22

Interaction with HSA: fluorescence and ^{1}H NMR spectra SI-23

Modified Rapid Equilibrium Dialysis setup SI-25

Cytotoxicity data SI-26

NMR spectra for characterization SI-28

Conditions applied for steady-state spectrofluorometric measurements SI-33

References SI-33

Interpretation of the IR spectra

Interpretation of the mid-IR spectra

Functional group region (characteristic stretching vibrations): C-H stretching modes

The most important regions of the mid-IR spectra are shown in Figure S1. The region between 3100 and 2800 cm^{-1} shows the C-H stretching modes of the ligands (both the arene/arenyl and the bidentate ligands).

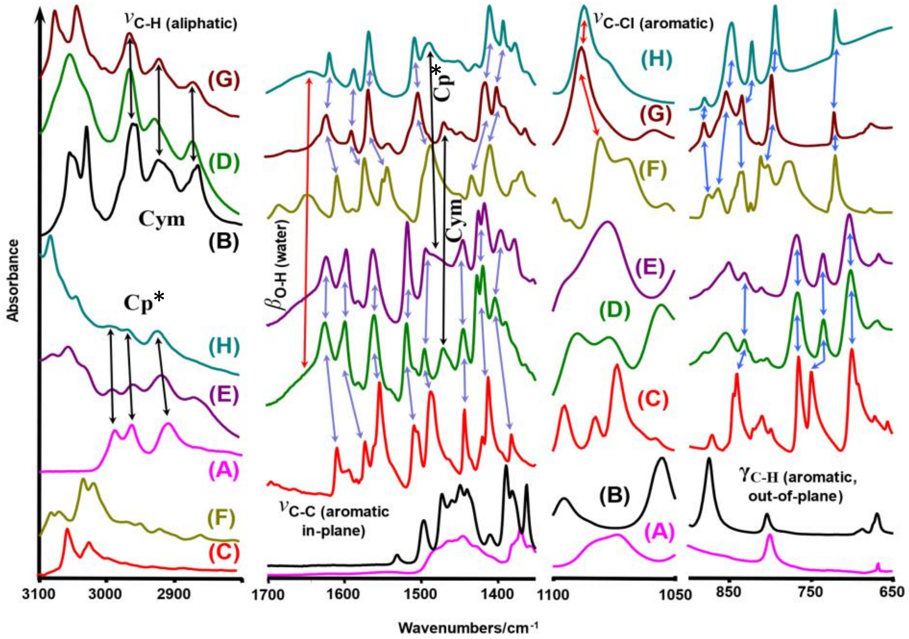

Figure S1. Selected regions of the mid-IR ATR spectra of the prepared complexes and their starting materials: (A) [RhCp*(μ-Cl)Cl]$_2$, (B) [RuCym(μ-Cl)Cl]$_2$, (C) BP, (D) [RuCym(BP)Cl]Cl, (E) [RhCp*(BP)Cl]Cl, (F) DCP, (G) [RuCym(DCP)Cl]Cl, and (H) [RhCp*(DCP)Cl]Cl. Black double arrows in the $\nu_{\text{C-H (aliphatic)}}$ region: stretching modes of the aliphatic C-H bonds of Cp* and Cym. Blue double arrows in the $\nu_{\text{C-C (aromatic, in-plane)}}$ region: in-plane stretching modes of the aromatic rings of the BP and DCP ligands. Red double arrows: in-plane scissoring mode ($\beta_{\text{O-H (water)}}$) of the incorporated water. Black double arrows: a weak group of bands identified as the deformation modes the aliphatic parts of Cym; $\nu_{\text{C-Cl (aromatic)}}$ region, stretching mode of the C–Cl bonds in the DCP ligand, strongly coupled with the in-plane stretching modes of the aromatic rings, shifted to higher wavenumbers due to complexation. Black double arrows: a characteristic band of Cym. Blue double arrows: $\gamma_{\text{C-H (aromatic, out-of-plane)}}$ region dominated by the out of plane deformation modes of the C-H groups of the aromatic rings.

The bands above 3000 cm^{-1} are usually assigned to the aromatic C-H groups and they are difficult to interpret. On the other hand, the bands bellow 3000 cm^{-1}, originating the aliphatic parts of the ligands, are easy to assign. They originate from the methyl and *iso*-propyl groups of the π-bonded arene/arenyl ligands either in their dimeric precursor complexes or in the title complexes. The stretching modes of the methyl groups can be easily identified in the spectra of *p*-cymene containing complexes. The asymmetric stretching modes ($\nu_{as}CH_3$-) of the *iso*-propyl group at around 2960 cm^{-1} can be distinguished from the similar mode of the methyl group, at around 2925 cm^{-1}, bonded directly to the aromatic ring. The symmetric stretching modes of these methyl groups (ν_sCH_3-) overlap each other at around 2970 cm^{-1} in the aliphatic νC-H region of the spectra B, D and G in Figure S1).[1] The aliphatic C-H stretching region of the complexes containing Cp* ligand, is more complicated to interpret. The interpretation of the vibrational spectra of the alkaline complexes of cyclopentadienyl-anion and its pentamethyl derivative were performed by using D_{5h} local symmetry by Mink *et al.*[2] The C-H stretching region was omitted from the study, while Amberger *et al.*[3] used C_{5v} local symmetry for the rings and D_{5h} as overall symmetry of their complexes. They measured four IR-active bands between 3100 and 2700 cm^{-1} region, although local symmetry predicts six IR- and Raman-active vibrations ($3A_1$+$3E_1$), with transitional dipole moments oriented along the z-axis and x,y axes, respectively. Our spectra of the Cp*-containing complexes (Figure S1, A, E, H) exhibited three well-defined bands in the $\nu_{as}CH_3$ region (2989, 2963 and 2909 cm^{-1}), while multiple overlapping bands appeared in the ν_sCH_3 region at lower wavenumbers. The direct assignment of the symmetric C-H stretching bands was complicated due to the strong overlap of the bands and the presence of the overtones of the deformation modes of the methyl-group appearing at around 1460 cm^{-1}.

Fourier deconvolution and peak fitting was applied in the 3075 – 2575 cm^{-1} range, to identify the three strongest bands in the ν_sCH_3 region (Figure S2). Three of the fitted bands have proper intensities to identify as symmetric C-H stretching modes at 2864, 2825 and 2764 cm^{-1}, probably due to the overtones mentioned earlier. Further two peaks, one with negative intensity were fitted to the range bellow 2700 cm^{-1} in order to describe that part of the spectrum and to reduce the absolute value of the RMS-noise.

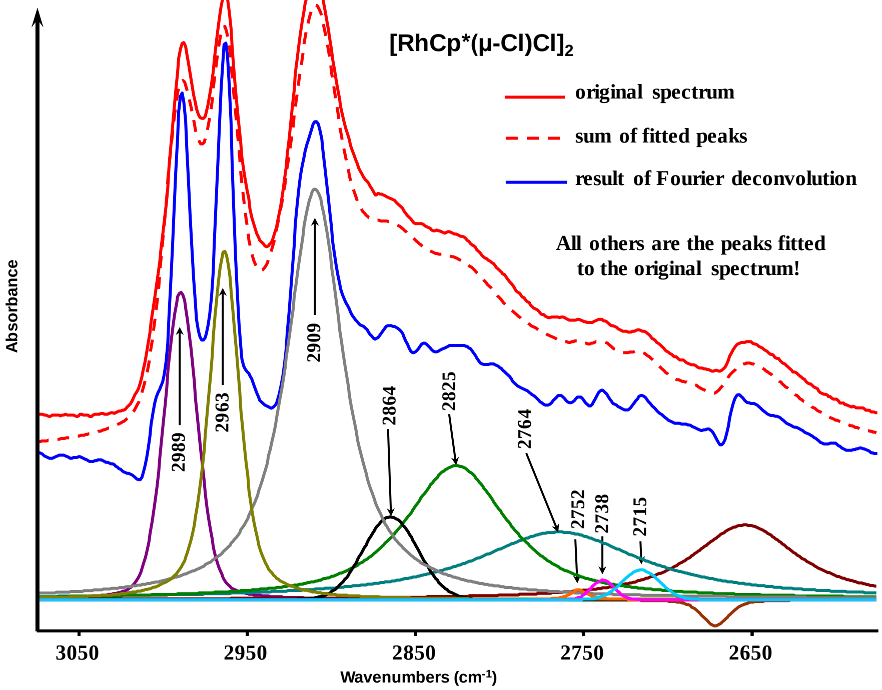

Figure S2. Fourier deconvolution of ATR-IR spectra of the metal precursor [Rh(η^5-C_5Me_5)Cl_2]$_2$ with peak fitting in the range of the aliphatic C-H stretching modes, to identify the ν_sCH_3 modes of the Cp*-ligand. The bands at 2864, 2825 and 2764 cm^{-1} can be assigned as the three A_1 modes deduced from the C_{5v} local symmetry.

C-C stretching modes

The region between 1700 and 1350 cm^{-1} shows strong bands originated from the in-plane C-C stretching modes of the aromatic rings, and the in-plane deformation mode (β_{O-H}) of the water molecule at around 1640 cm^{-1} (Figure S1). This peak is well separated from the ring modes in the spectra of complexes with DCP and overlaps with them in the spectra of the BP-containing complexes, but easily distinguishable from them since it is much broader than the ring modes. The alteration of the most intense peaks of the in-plane C-C stretching modes are the best indicators of the changes in the electronic structure of the substituted PHEN ligands due to complex formation, but they are not too sensitive on the nature of the coordinated metal ion.[3] Our spectra confirm both parts of this statement, since the bands of the free ligands are long way away from the bands of the complexed ligands, but there are only slight differences between the spectra of the same ligand bonded to different metal ions (Figure S1, range $\nu_{C-C\ (aromatic,\ in\text{-}plane)}$, C,D and E or F,G and H spectra).

Broader bands of the deformation modes of the methyl groups of the π-bonded ligands Cp* and *p*-cymene can also be identified in the ranges of 1475-1490 cm^{-1} and 1450-1470 cm^{-1}, respectively (Figure S1, range $\nu_{C\text{-}C\ (aromatic,\ in\text{-}plane)}$, D-H spectra).

It should be noted that certain in-plane aromatic C-C stretching modes of the coordinated ligands are observed at higher wavenumbers compared to those of the free ligands. This cannot simply be explained by the formation of σ-bonds between the lone pairs of the nitrogen donor atoms of BP and DCP and the metal ions, since only the weakening of the ring C-C σ-bonds would be expected to cause a shift to lower wavenumbers. Since the local symmetry (C_{2v}) remains unchanged, and this point group does not contain any degenerated species, splitting of the vibrations is not expected to occur. Notably, PHEN-type ligands are known not only as σ-donors but also as π-acceptors. The higher wavenumbers can be explained by considering the contribution of the ring π-electrons to the metal–ligand bonding. To verify this supposition, simple Hückel-type calculations were performed using the program HuLis (version 3.3.7),[4,5] which can handle heteronuclear atoms. The π-electron densities were calculated for both studied ligands (DCP, BP) and PHEN for comparison, while their doubly positively charged versions were the models of the complexed ligands (Figures S3-S5). Comparison of the corresponding results shows that the π-electron densities of the rings shifted from the more even distribution – from the benzoid-like state – to the less evenly distributed – quinoid-like state – , causing alternating increase and decrease of bond order in every second π-bond within the rings. This effect shifts bands to higher wavenumbers that include the internal coordinates with increased force constants, and bands at lower wavenumbers that include those internal coordinates with decreased force constants, instead of bands that incorporated all of those internal coordinates.

All three examples support that the bonding structure of the π-system shifts from the benzenoid-like structure to a more quinoid-like structure. The C-C bond orders are less even in a quinoid-like structure, so the contribution of the adjacent C-C bonds differs more and more to the same normal mode. Higher contribution from the bonds with higher bond order causes an increase in the energy of the normal modes in question, while a higher contribution from the bonds with lower bond order shifts these modes to lower wavenumbers.

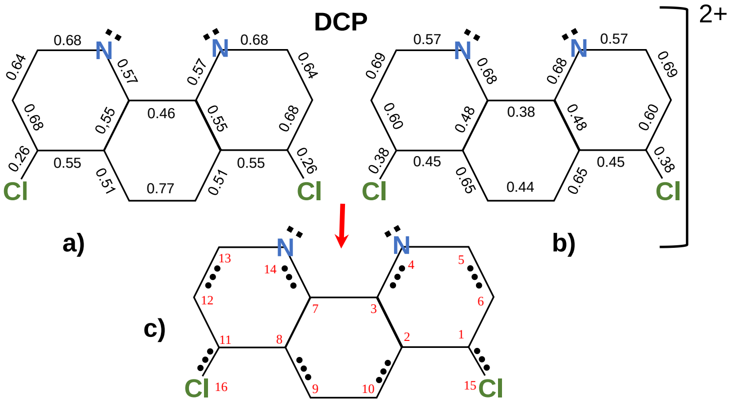

Figure S3. The results of the Hückel calculations on DCP and DCP^{2+} ligands simulating the effects of complexation on the π-electron system. The order of the π-bonds between the given C-C bonds is given in structure (A) and (B) respectively. Structure (C) shows the qualitative changes in the π-electron system and the atom-numbering employed for the calculations. The π-bond order increases along the dotted bonds and decreases along the non-marked bonds showing the less even distribution of the π-electrons in the aromatic rings.

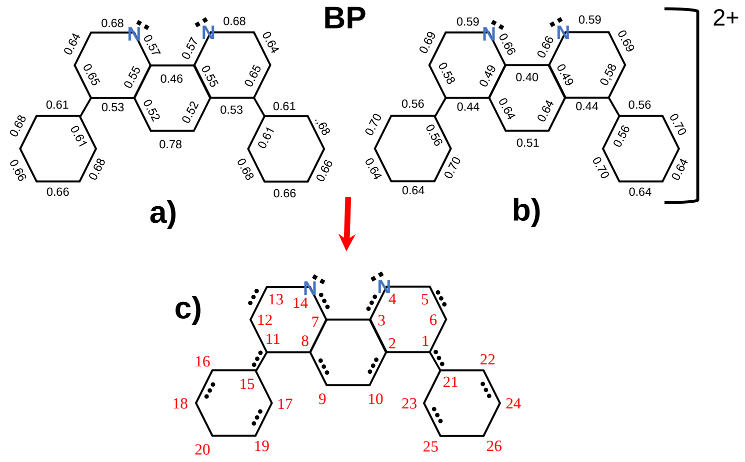

Figure S4. The results of the Hückel calculations on BP and BP^{2+} ligands simulating the effects of complexation on the π-electron system. The order of the π-bonds between the given C-C bonds is given in structure (A) and (B) respectively. Structure (C) shows the qualitative changes in the π-electron system and the atom-numbering employed for the calculations. The π-bond order increases along the dotted bonds and decreases along the non-marked bonds showing the less even distribution of the π-electrons in the aromatic rings.

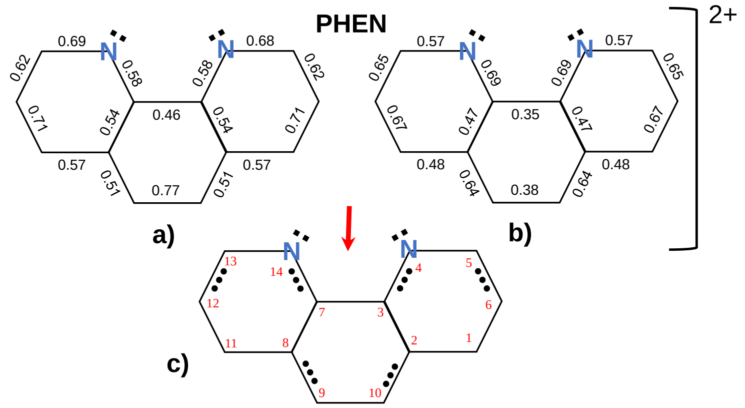

Figure S5. The results of the Hückel calculations on PHEN and $PHEN^{2+}$ ligands simulating the effects of complexation on the π-electron system. The order of the π-bonds between the given C-C bonds is given in structure (A) and (B) respectively. Structure (C) shows the qualitative changes in the π-electron system and the atom-numbering employed for the calculations. The π-bond order increases along the dotted bonds and decreases along the non-marked bonds showing the less even distribution of the π-electrons in the aromatic rings.

Fingerprint region (bending and skeletal vibrations):

Comparison of the ranges between 1100 and 1050 cm^{-1} of the DCP containing complexes and the free ligand verified the results of the Hückel-calculations. The band assigned to the C-Cl stretching mode is strongly coupled with the ring modes, since the chlorine atom is in conjugation with the ring.[1] The corresponding band shifted from 1080 cm^{-1} to 1087 cm^{-1} and to 1088 cm^{-1} in the spectra of the free DCP ligand, RhCp*DCP and to the RuCymDCP complexes, respectively, indicating the predicted increase in the bond order along the C-Cl bond (Figure S1, range $\nu_{\text{C-Cl (aromatic)}}$, F-H spectra).

The range between 875 and 650 cm^{-1} displays the out-of-plane deformation modes of the aromatic rings (Figure S1, range $\nu_{\text{C-H (aromatic, out-of-plane)}}$, A-H spectra). Since the spectra of fused ring aromatics have characteristic bands in the same regions as benzene derivatives, the out-of-plane region also shows strong characteristic bands in the range between 875 and 650 cm^{-1}.[6] The interpretation of this part of the spectra is usually based on the number of adjacent hydrogen atoms.[1] Most of these bands shift to lower wavenumbers on complex formation since they are associated with the σ-bonds of the rings.

Interpretation of the far-IR spectra recorded for the complexes, the metal precursors and the ligands

The far-IR spectra of the complexes and ligands are shown in Figures S6, S7 between 500 and 100 cm^{-1}. The bands can be classified into four categories. The most important groups are the stretching modes of metal – π bonded ligands, metal – chlorido ligand stretching modes and those of the metal – N stretching modes of DCP and BP. Their alterations indicate the changes in the metal-ligand bonds. Last but not least there could be bands characterized by the internal vibrations of ligands DCP and BP in this region. Great difference is expected between the intensities of these bands, since the polarization of the above bonds are very different. The stretching modes of the metal – chlorido bonds must be the most intense peaks. Similar intensity may be characteristic for the stretching modes of the Rh–Cp* bond, since this ligand is also negatively charged, while with the neutral arene ligand, the Ru-Cym bond is much less polarized, so its stretching mode gives a band with low intensity. The internal vibrations of DCP and BP are expected to be the weakest.

The precursors $[Rh(\eta^5\text{-}C_5Me_5)Cl_2]_2$ and $[Ru(\eta^6\text{-}p\text{-cymene})Cl_2]_2$ are held together by two bridging μ_2-chlorido ligands and both contain two additional terminally coordinated chlorido ligand. The local symmetry of these parts of the complexes is C_{2h}. It predicts $3A_g$ + $3B_u$ modes for the stretching modes of the six metal-chlorido bonds. The $3B_u$ modes are infrared active and two of them belong to the bridging and one to the terminal chlorido ligand. The bond order for a M-Cl stretching mode of a terminally bonded one is always higher than that for the bridging one, since its force constant is highest. So, the highest wavenumber band around 275 cm^{-1} should be assigned to the terminal Rh-Cl bonds and two of the strongest bands bellow it to the bridging Rh-Cl-Rh bonds (Figure S5). Since that part of the spectrum seemingly contains number of overlapping bands, Fourier deconvolution and peak-fitting were performed to identify the components. Similar treatment was performed on the far-IR spectrum of the Ru(II)-dimer (Figure S7).

The presence of four strong band in this range of the $[RuCym(\mu\text{-}Cl)Cl]_2$ could cause some uncertainty in the assignment of the stretching modes of the Ru-Cl-Ru moiety, but the clear assignment of the similar bands of the $[RhCp^*(\mu\text{-}Cl)Cl]_2$ dimer gave us a clear guide, since the force constants of similar moieties must be in the same magnitude, since that is the base of the ‘group frequency method’ employed in the practical interpretation of vibrational

spectra, so the difference in wavenumbers, depending on the so called ‘interaction force constant’, must be similar of the given bands in both spectra. That difference in the spectrum of the rhodium dimer was 47 cm^{-1}, so only the difference between the bands at 260 and 209 cm^{-1}, 51 cm^{-1} satisfied this condition in the spectrum of the ruthenium dimer.

Unfortunately, the stretching bands between the metal and the π-bonded ligands cannot be identified, nor can the stretching bands of the metal-nitrogen bonds. However, some of the vibrations characteristic of the ligands appear in the spectra of the complexes prepared.

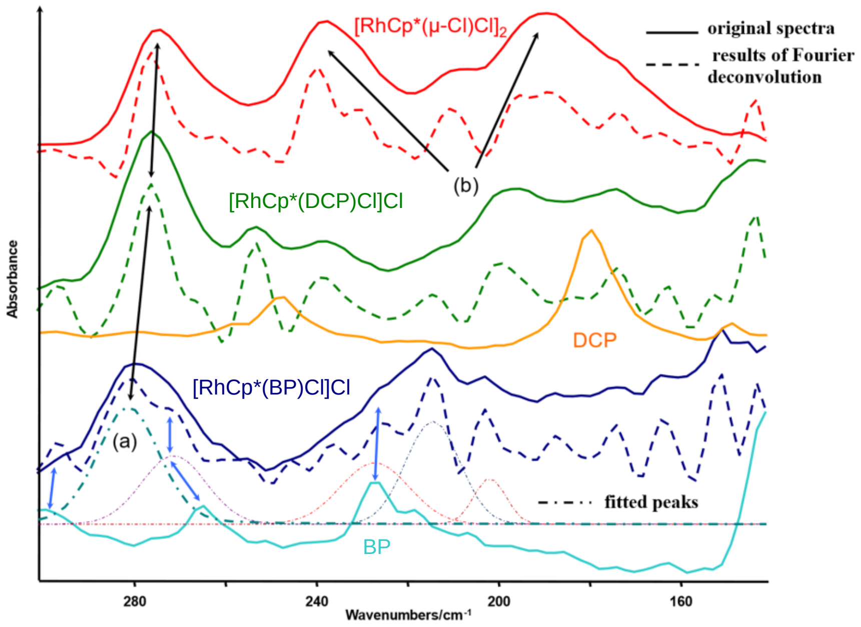

Figure S6. ATR-IR spectra of the RhCp* complexes and the corresponding ligands in the far-IR region. Bands marked (a) are assigned to the stretching modes of the Rh(III) – chlorido bond at 274, 276 and 280 cm^{-1} for the complexes [RhCp*(μ-Cl)Cl]$_2$, [RhCp*(DCP)Cl]Cl and [RhCp*BP(Cl)]Cl, respectively. The bands marked (b) were assigned to the stretching modes of the bonds between the metal and the bridging chlorido ligand of the dimeric precursor.

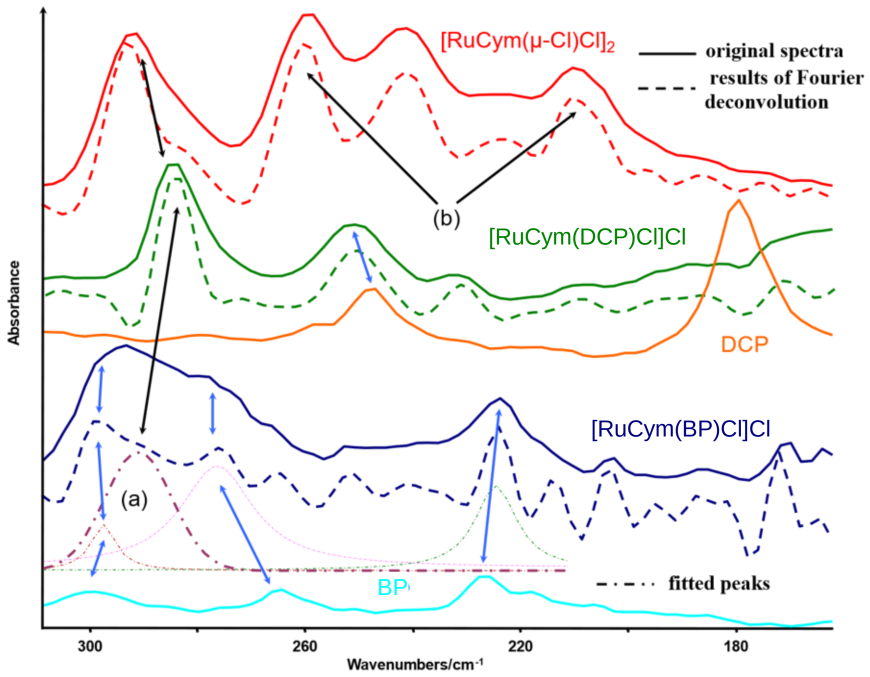

Figure S7. ATR-IR spectra of the RuCym complexes and the corresponding ligands in the far-IR region. Bands marked (a) are assigned to the stretching modes of the Ru(II) – chlorido bond at 293, 284 and 298 cm^{-1} for the complexes [RuCym(μ-Cl)Cl]$_2$, [RuCym(DCP)]Cl and [RuCym(BP)]Cl, respectively. The bands marked (b) were assigned to the stretching modes of the bonds between the metal and the bridging chloride ligand of the dimer.

Table S1. Crystal data and structure refinement for $[RuCym(BP)Cl]PF_6 \times Et_2O$ and $[RhCp^*(DCP)Cl)]PF_6$.

Identification code	$[RuCym(BP)Cl]PF_6 \times Et_2O$ (**I**)	$[RhCp^*(DCP)Cl)]PF_6$ (**II**)
CCDC number	2422759	2422760
Empirical formula	$C_{38}H_{40}ClF_6N_2OPRu$	$C_{22}RhCl_4N_2PF_6H$
Formula weight	822.236	682.946
Temperature/ K	153.15	113 K
Crystal system	triclinic	orthorhombic
Space group	*P*-1	*Pbca*
a / Å	15.0576(8)	13.3924(3)
b / Å	17.2246(12)	12.3719(2)
c / Å	17.6351(9)	29.1094(5)
α / °	81.889(7)	90
β / °	65.118(5)	90
γ / °	64.323(4)	90
Volume / $Å^3$	3734.8(4)	4823.12(16)
Z/Z'	4/2	8/1
ρ_{calc} / g/cm^3	1.462	1.839
μ/mm^{-1}	5.002	9.989
F(000)	1688.8	2677.3
Crystal size / mm^3	0.2 × 0.2 × 0.1	0.3 × 0.2 × 0.1
Radiation (λ)	Cu Kα (1.54187)	Cu Kα (1.54187)
2Θ range for data collection / °	7.02 to 144.16	6.08 to 136.5
Index ranges	$-18 \le h \le 17$, $-21 \le k \le 21$, $-21 \le l \le 21$	$-16 \le h \le 15$, $-13 \le k \le 13$, $-34 \le l \le 35$
Reflections collected	43460	39156
Independent reflections (R_{int}, R_{sigma})	13813 (0.1028, 0.1318)	4266 (0.1400, 0.1113)
Data/restraints/parameters	13813/112/909	4266/0/321
Goodness-of-fit on F^2	1.025	1.068
Final R indexes [I>=2σ (I)] (R_1,wR_2)	0.1069, 0.2885	0.0549, 0.1172
Final R indexes [all data] (R_1,wR_2)	0.1702, 0.3376	0.0740, 0.1266
Largest diff. peak/hole / e $Å^{-3}$	1.92/-2.59	1.99/-2.02

Table S2. Bond Lengths for [RuCym(BP)Cl]PF_6×Et_2O and [RhCp*(DCP)Cl)]PF_6.

[RuCym(BP)Cl]PF_6×Et_2O

**Atom**	**Atom**	**Length / Å**	**Atom**	**Atom**	**Length / Å**
Ru1	Cl1	2.389(3)	Ru2	Cl2	2.386(2)
Ru1	N1	2.079(8)	Ru2	N3	2.055(9)
Ru1	N2	2.074(9)	Ru2	N4	2.097(9)
Ru1	C1	2.198(10)	Ru2	C41	2.235(9)
Ru1	C2	2.174(10)	Ru2	C42	2.160(13)
Ru1	C3	2.167(10)	Ru2	C43	2.174(15)
Ru1	C4	2.187(12)	Ru2	C44	2.178(14)
Ru1	C5	2.149(12)	Ru2	C45	2.189(15)
Ru1	C6	2.210(9)	Ru2	C46	2.129(12)

[RhCp*(DCP)Cl)]PF_6

**Atom**	**Atom**	**Length / Å**
Rh1	Cl3	2.3849(14)
Rh1	N2	2.123(4)
Rh1	N1	2.123(5)
Rh1	C4	2.153(5)
Rh1	C5	2.157(6)
Rh1	C2	2.156(5)
Rh1	C1	2.195(6)
Rh1	C3	2.177(5)

Table S3. Bond Angles for [RuCym(BP)Cl]PF_6×Et_2O and [RhCp*(DCP)Cl)]PF_6.

[RuCym(BP)Cl]PF_6×Et_2O

Atom	Atom	Atom	Angle / °	Atom	Atom	Atom	Angle / °
N1	Ru1	Cl1	86.1(2)	C44	Ru2	C41	77.1(5)
N2	Ru1	Cl1	85.2(2)	C44	Ru2	C42	66.9(8)
N2	Ru1	N1	76.4(3)	C44	Ru2	C43	38.0(8)
C1	Ru1	Cl1	157.2(4)	C45	Ru2	Cl2	101.8(5)
C1	Ru1	N1	95.6(4)	C45	Ru2	N3	172.2(5)
C1	Ru1	N2	117.4(4)	C45	Ru2	N4	106.4(7)
C2	Ru1	Cl1	152.4(3)	C45	Ru2	C41	64.0(6)
C2	Ru1	N1	120.9(4)	C45	Ru2	C42	78.0(6)
C2	Ru1	N2	95.2(4)	C45	Ru2	C43	68.1(7)
C2	Ru1	C1	37.1(4)	C45	Ru2	C44	36.8(8)
C3	Ru1	Cl1	116.7(3)	C46	Ru2	Cl2	133.1(5)
C3	Ru1	N1	156.4(4)	C46	Ru2	N3	139.9(5)
C3	Ru1	N2	98.1(4)	C46	Ru2	N4	91.2(5)
C3	Ru1	C1	66.1(4)	C46	Ru2	C41	35.3(5)
C3	Ru1	C2	35.8(4)	C46	Ru2	C42	65.2(5)
C4	Ru1	Cl1	91.0(3)	C46	Ru2	C43	79.1(5)
C4	Ru1	N1	160.5(4)	C46	Ru2	C44	64.6(7)
C4	Ru1	N2	122.5(4)	C46	Ru2	C45	35.0(6)

[RhCp*(DCP)Cl)]PF_6

Atom	Atom	Atom	Angle / °	Atom	Atom	Atom	Angle / °
N2	Rh1	Cl3	90.46(13)	C2	Rh1	C5	64.8(2)
N1	Rh1	Cl3	86.74(12)	C1	Rh1	Cl3	93.20(17)
N1	Rh1	N2	77.34(17)	C1	Rh1	N2	135.6(2)
C4	Rh1	Cl3	154.43(18)	C1	Rh1	N1	147.0(2)
C4	Rh1	N2	96.61(19)	C1	Rh1	C4	64.8(2)
C4	Rh1	N1	118.8(2)	C1	Rh1	C5	38.1(2)
C5	Rh1	Cl3	115.62(19)	C1	Rh1	C2	38.8(2)
C5	Rh1	N2	102.03(19)	C3	Rh1	Cl3	143.76(17)
C5	Rh1	N1	157.6(2)	C3	Rh1	N2	125.6(2)
C5	Rh1	C4	38.8(2)	C3	Rh1	N1	97.2(2)
C2	Rh1	Cl3	106.22(16)	C3	Rh1	C4	38.9(2)
C2	Rh1	N2	161.92(19)	C3	Rh1	C5	64.6(2)
C2	Rh1	N1	109.9(2)	C3	Rh1	C2	38.7(2)
C2	Rh1	C4	65.3(2)	C3	Rh1	C1	64.5(2)

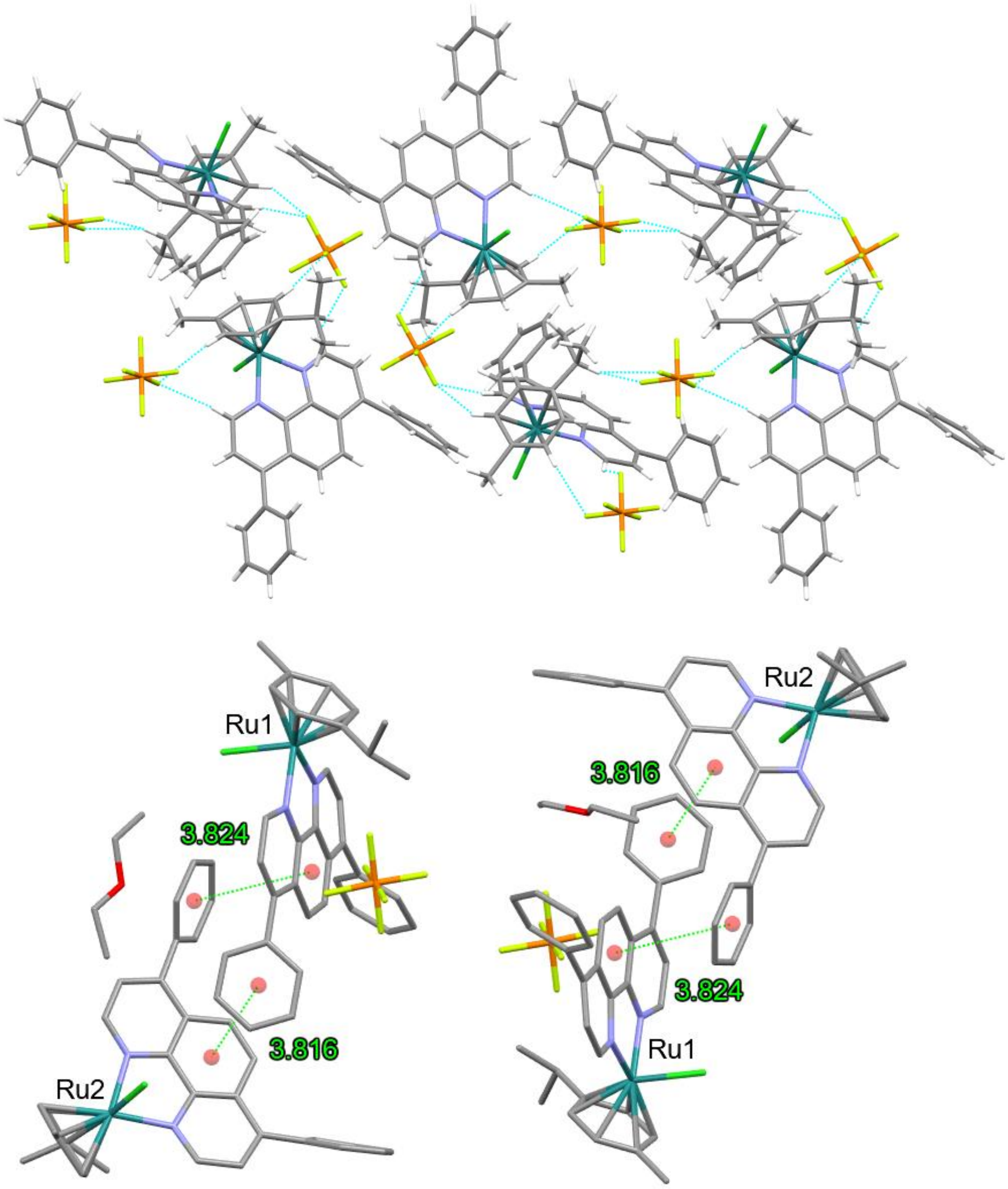

Figure S8. Packing arrangements of the [RuCym(BP)Cl]$^+$ complexes in crystal **I** showing two different views. The upper figure shows the P-F…H interactions and the bottom figure shows the π…π intermolecular interactions between pairs of complexes.

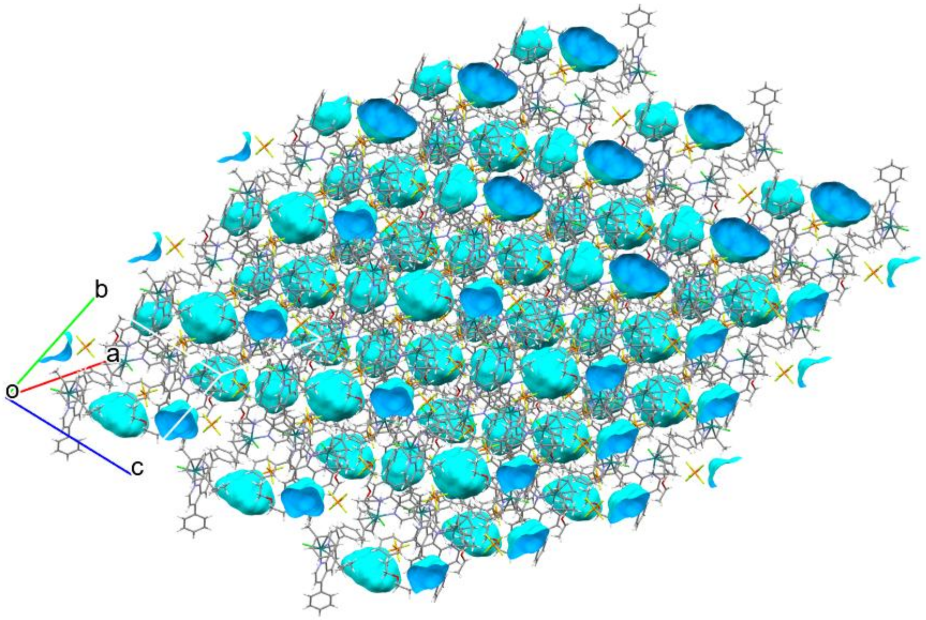

Figure S9. The [RuCym(BP)Cl]PF_6×Et_2O crystal contains voids of 207.4 Å^3 which is 5.6% of the unit cell volume (calculated by Mercury software).

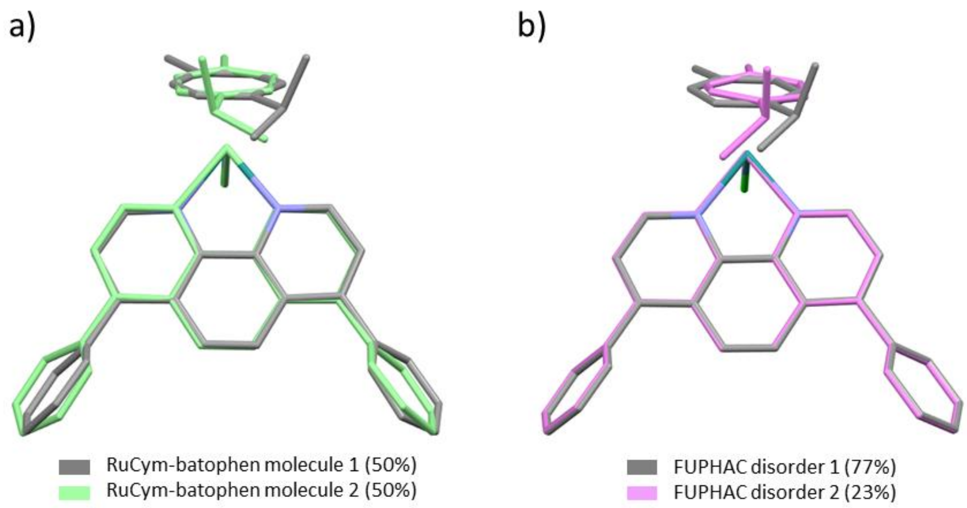

Figure S10. Conformational comparison of the two $[RuCym(BP)Cl]^+$ complexes obtained (a) in crystal **I** of $[RuCym(BP)Cl]PF_6{\times}Et_2O$ measured at 153 K and (b) in crystal FUPHAC[7] measured at room temperature. The FUPHAC crystal was measured at room temperature, where the rotational energy of the molecules is high, so that the *p*-cymene ring in the molecule can easily rotate. This resulted in a major and a minor conformation in 77/23%. This structure was refined in the *C*2/*c* space group, which contains an inversion center, a two-fold rotation axis, a two-fold screw axis along the *b* axis, and a glide plane perpendicular to these, in the *ac* plane. In contrast, for crystal **I,** measured at 153 K, a more ordered structure was obtained, with the two conformations present in a 50-50% alternation, resulting in two crystallographically independent complexes (molecule 1 and molecule 2) within the asymmetrical unit (Figure S10a). In this case, only the inversion center remains as a symmetry element and the space group is *P*-1. The conformation of molecule 1 in crystal **I** is very similar to that of the major disorder in FUPHAC, while the conformation of molecule 2 differs significantly from the minor disorder (compare Figure S10a and b). The largest conformational differences are in the positions of the *iso*-propyl group of the *p*-cymene ring and the two phenyl substituents of the PHEN ring (Figure S10). Despite these conformational differences, the packing is very similar in the two crystals (see Figure S11).

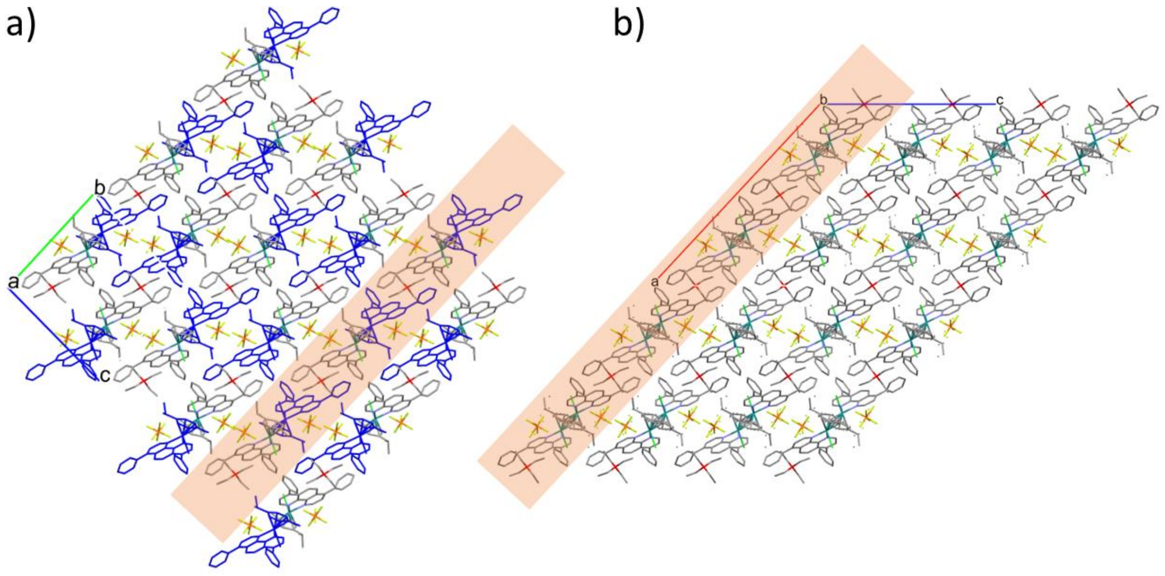

Figure S11. Comparison of the unit cell and the packing of molecules in (a) crystal [RuCym(BP)Cl]PF_6×Et_2O (**I**) (molecule 2 is colored in blue) and (b) in crystal Ref. code FUPHAC.[30] Similar columns are highlighted in orange.

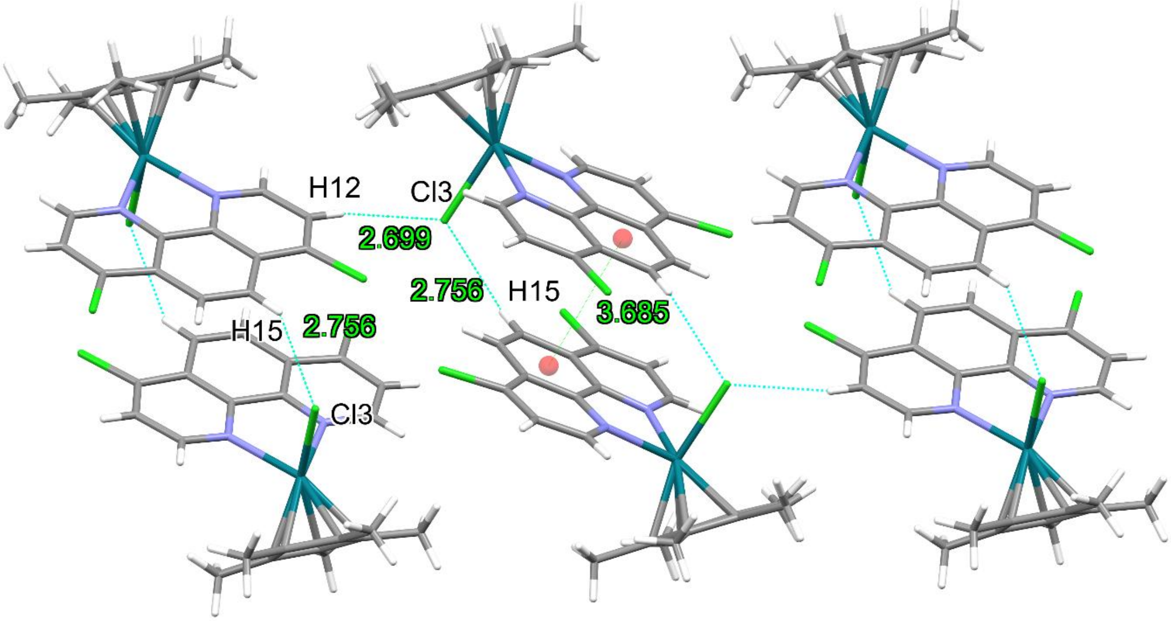

Figure S12. Packing arrangements of the complexes in crystal [RhCp*(DCP)Cl]PF_6 showing the H-bond and $\pi\ldots\pi$ interactions between neighboring complexes.

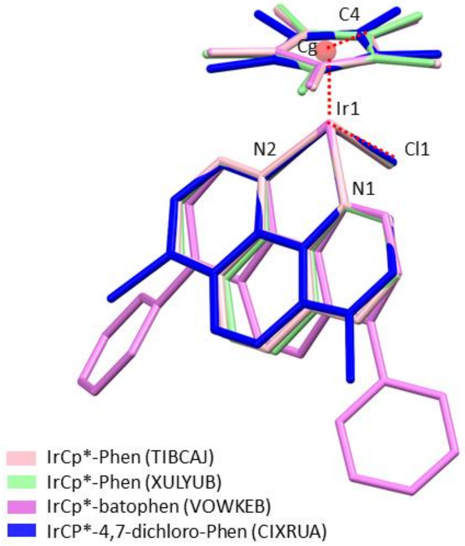

Figure S13. Overlaid structures of $[IrCp^*(PHEN)Cl]^+$ complexes (TIBCAJ and XULYUB) with related BP (VOWKEB) and DCP (CIXRUA) derivatives found in the CSD. Atom numbering used in Table S4 and the torsion angle of C4-Cg-Ru1-Cl1 with red scattered lines are indicated.

Table S4. Comparison of selected bond distances and angles for [RuCym(L)(Cl)] and [RhCp*(L)(Cl)] complexes with relevant 4,7-disubstituted-phenantroline (PHEN) derivatives.

**Ligand (L)**[a]	**Distances(Å)**				**Angles (°)**				
	**M-Cg**	**M-Cl1**	**M-N1**	**M-N2**	**Cg-M-Cl1**	**N1-M-N2**	**Cl1-M-N1**	**C4-Cg-M-Cl**	**Ring-Ring**[b]
RuCym-(L)Cl									
PHEN	1.686	2.3993(9)	2.095(3)	2.096(3)	128.7	77.8(1)	84.20(8)	137.6	58.4
4-methyl-PHEN	1.684	2.4049(8)	2.090(2)	2.090(2)	129.0	77.69(9)	86.28(7)	16.5	47.6
4,7-dimethyl-PHEN	1.683	2.407(1)	2.105(2)	2.096(3)	127.8	77.61(9)	87.14(7)	144.7	57.2
BP (disorder)	1.677	2.380(2)	2.085(8)	2.088(4)	126.9	77.2(2)	85.9(2)	24.4	56.1
BP, molecule 1	1.670	2.388(2)	2.073(8)	2.070(8)	126.4	75.9(3)	86.1(2)	29.3	56.8
BP, molecule 2	1.682	2.382(2)	2.067(9)	2.111(8)	127.3	77.7(3)	84.7(2)	0.4	57.2
Average	1.680(6)	2.39(1)	2.09(1)	2.09(1)	128(1)	77.3(7)	86(1)		55(4)
RhCp*-(L)Cl									
PHEN	1.781	2.386(2)	2.109(4)	2.129(4)	126.1	77.5(2)	83.6(1)	-1.8	62.4
PHEN	1.780	2.406(1)	2.121(2)	2.100(2)	127.6	77.68(8)	86.46(6)	-19.4	53.7
3,4,7,8-tetramethyl-PHEN	1.778	2.392(2)	2.102(6)	2.112(5)	126.9	77.3(2)	87.0(2)	6.6	51.1
4,7-dichloro-PHEN	1.785	2.3845(8)	2.117(2)	2.124(2)	126.7	77.16(7)	86.55(5)	10.0	48.2
4,7-dichloro-PHEN	1.791	2.3849(14)	2.123(5)	2.123(4)	126.7	77.34(17)	86.74(12)	10.6	48.1
Average	1.783(6)	2.39(1)	2.11(1)	2.12(1)	127(1)	77.5(2)	86(2)		54(6)
IrCp*-(L)Cl									
PHEN	1.780	2.395(4)	2.12(1)	2.111(7)	127.0	76.3(4)	86.0(3)	-2.09	62.1
PHEN	1.782	2.3719(7)	2.112(2)	2.098(2)	126.5	77.32(7)	86.02(5)	0.75	59.9
4,7-dichloro-PHEN	1.791	2.3892(8)	2.108(2)	2.108(2)	127.5	76.94(8)	88.21(6)	8.92	49.81
BP	1.783	2.385	2.105	2.105	126.2	76.5	82.6	0.00	76.4
Average	1.784(5)	2.39(1)	2.111(6)	2.106(6)	126.8(6)	76.7(5)	86(2)		62(11)
OsCym-(L)Cl									
PHEN	1.678	2.4063(8)	2.092(3)	2.092(3)	129.0	77.2(1)	83.06(8)	137.7	59.7

**Ligand (L)**[a]	**Crystal data**			
	**Space group**	**R-factor (%)**	**T (K)**	**Ref. Code**
RuCym-(L)Cl				

PHEN	*Pbca*	3.70	RT	FUPGUV[7]
4-methyl-PHEN	*P*2$_1$/*c*	4.33	100K	DUCZOT[8]
4,7-dimethyl-PHEN	*P*2$_1$/*n*	4.80	RT	DUQHOP[8]
BP (disorder)	*C*2/*c*	6.94	RT	FUPHAC[7]
BP, molecule 1	*P*-1	10.5	153	crystal **1**
BP, molecule 2	*P*-1	10.5	153	crystal **1**
Average				
RhCp*-(L)Cl				
PHEN	*P*2$_1$/*n*	3.90	173	SANGAP[9]
PHEN	*P*-1	3.23	RT	XOFHAC[10]
3,4,7,8-tetramethyl-PHEN	*P*2$_1$/*n*	12.9	100	GOTTIU[11]
4,7-dichloro-PHEN	*Pbca*	2.76	173	CIXROU[12]
4,7-dichloro-PHEN	*Pbca*	5.5	113	crystal **2**
Average				
IrCp*-(L)Cl				
PHEN	*P* 2$_1$2$_1$2$_1$	4.35	RT	TIBCAJ[13]
PHEN	*P* 2$_1$/*c*	1.74	193	XULYUB[14]
4,7-dichloro-PHEN	*Pbca*	2.11	173	CIXRUA[12]
BP	*Ibam*	4.37	RT	VOWKEB[15]
Average				
OsCym-(L)Cl				
PHEN	*Pbca*	2.55	150	EDODEH [16]

[a] M = Ru1 or Rh1; Cg is the centre of gravity of Cym / Cp* ring, [b] Angle between the Cym / Cp* ring plane versus the PHEN ring plane in the complexes.

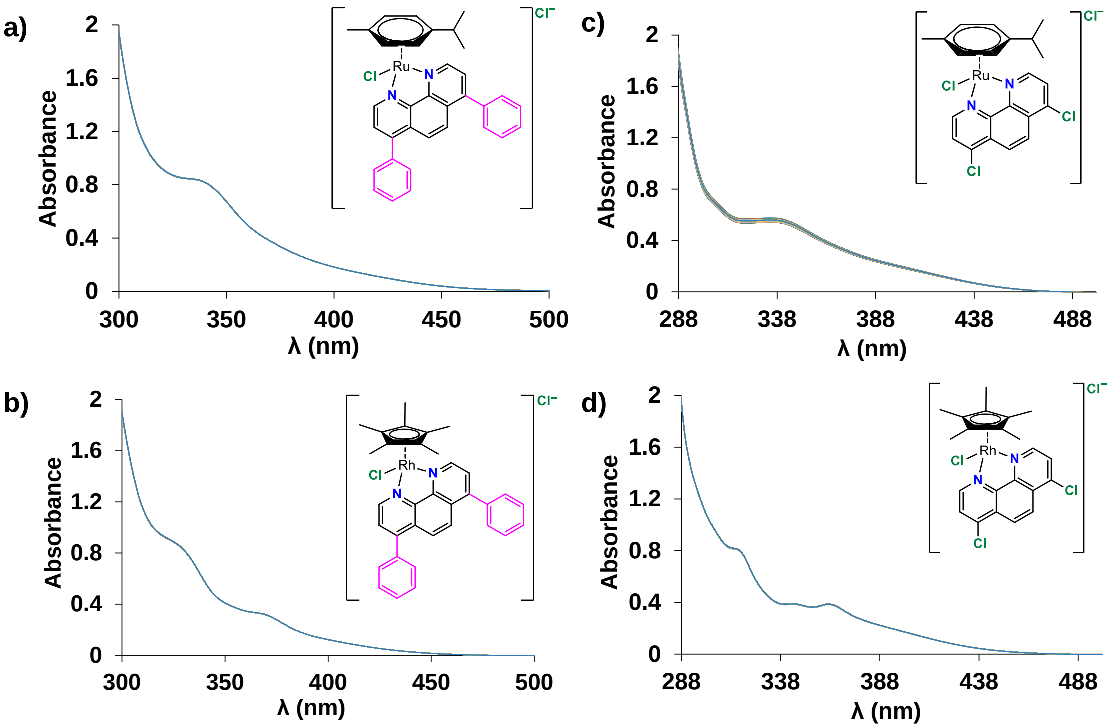

Figure S14. Time-dependent UV-Vis spectra of metal complexes (a) $c_{RuCymBP}$ = 69 µM; (b) $c_{RhCp*BP}$ = 66 µM; (c) $c_{RuCymDCP}$ = 99 µM; (d) $c_{RhCp*DCP}$ = 85 µM) in PBS' buffer over 24 h (a-d). Spectra were recorded in every 20 min.

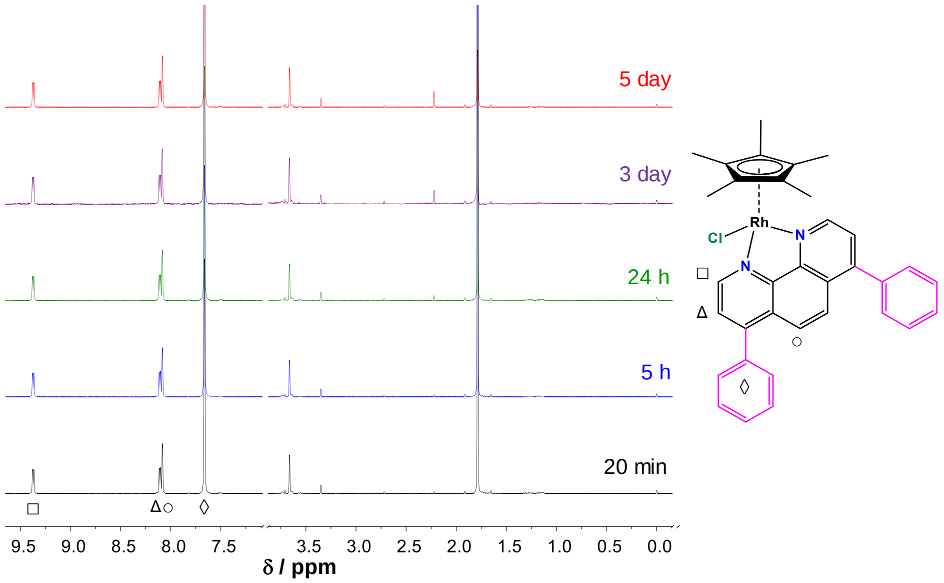

Figure S15. Time-dependent ^{1}H NMR spectra of metal complex RhCp*BP (66 µM) at pH 7.40 over 5 days. {10% (*v*/*v*) D_2O / 90% (*v*/*v*) H_2O; PBS'; *t* = 25 °C}

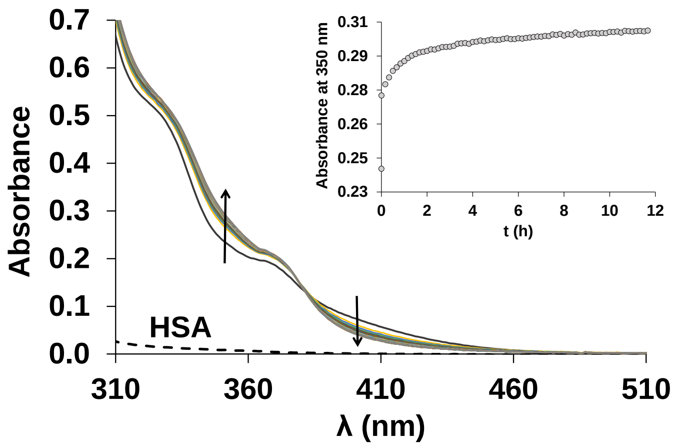

Figure S16. UV-Vis absorption spectra of the RhCp*BP complex – HSA system followed over 12 h, inserted figure shows the absorbance changes at 350 nm. {$c_{RhCp*BP}$ = 39 µM, c_{HSA} = 26 µM, PBS' buffer, t = 25.0 °C}

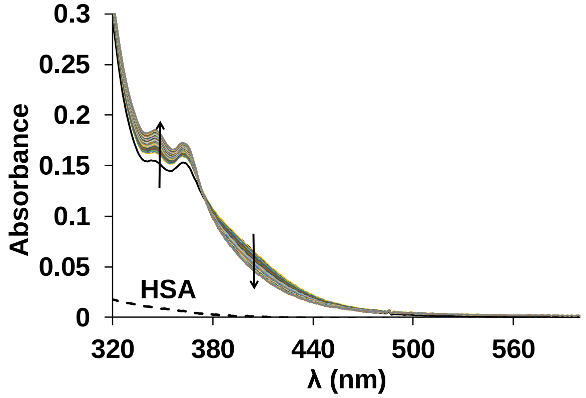

Figure S17. UV-Vis absorption spectra of the RhCp*DCP – HSA system followed over 12 h {$c_{RhCp*DCP}$ = 40 µM, c_{HSA}= 25 µM, PBS' buffer, t = 25.0 °C}.

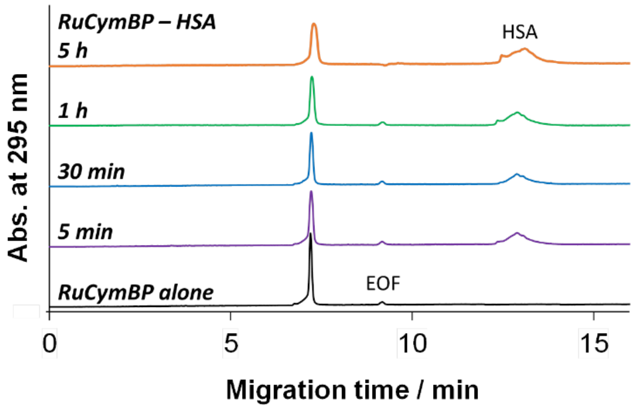

Figure S18. Electropherograms of RuCymBP in the absence and presence of 0.5 equivalent HSA followed in time; EOF: electroosmotic flow. {$c_{complex}$ = 95 µM, c_{HSA} = 48 µM; U = 8 kV; current = 105 µA; PBS'; t = 25 °C}.

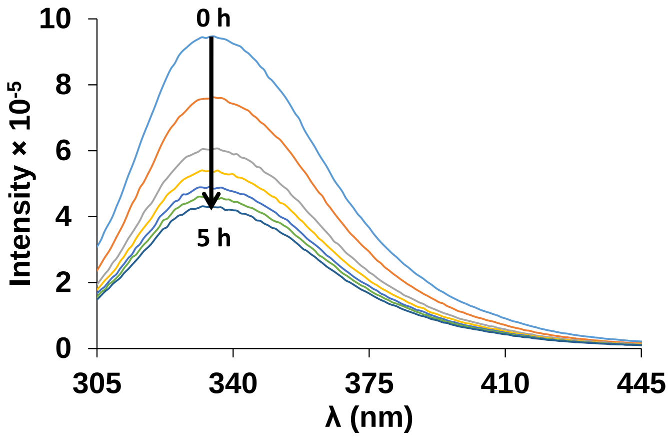

Figure S19. Fluorescence emission spectra of HSA in the presence of RhCp*BP over 5 h {$c_{\mathrm{RhCp*BP}}$ = 12.0 µM, c_{HSA} = 1.0 µM; PBS'; t = 25°C}.

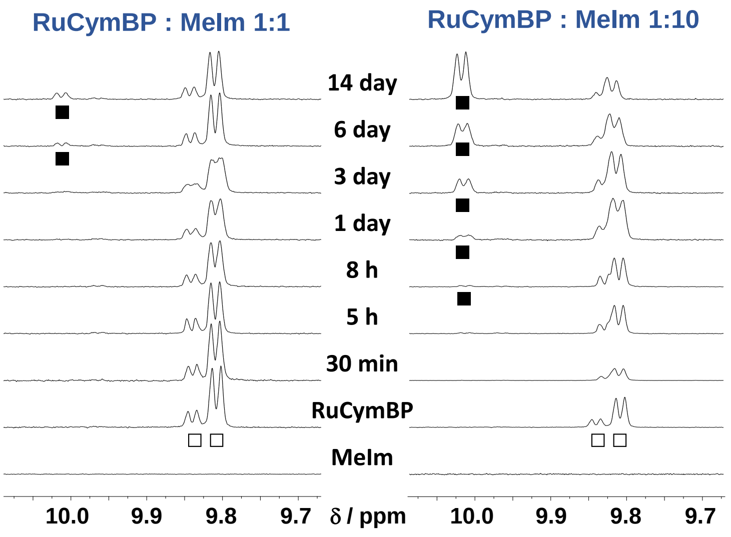

Figure S20. ^{1}H NMR spectra of RuCymBP – MeIm (1:1) and (1:10) systems in the region of the CH2 and CH9 protons followed over time, symbols denote: free metal complex forms (□) and the forming RuCymBP-MeIm ternary complex (■) {1:1 c_{RuCymBP} = 0.51 mM, c_{MeIm} = 0.53 mM; 1:10 c_{RuCymBP} = 0.50 mM, c_{MeIm} = 5.21 mM; 10% (v/v) D_2O / 90% (v/v) H_2O; PBS'; t = 25 °C }

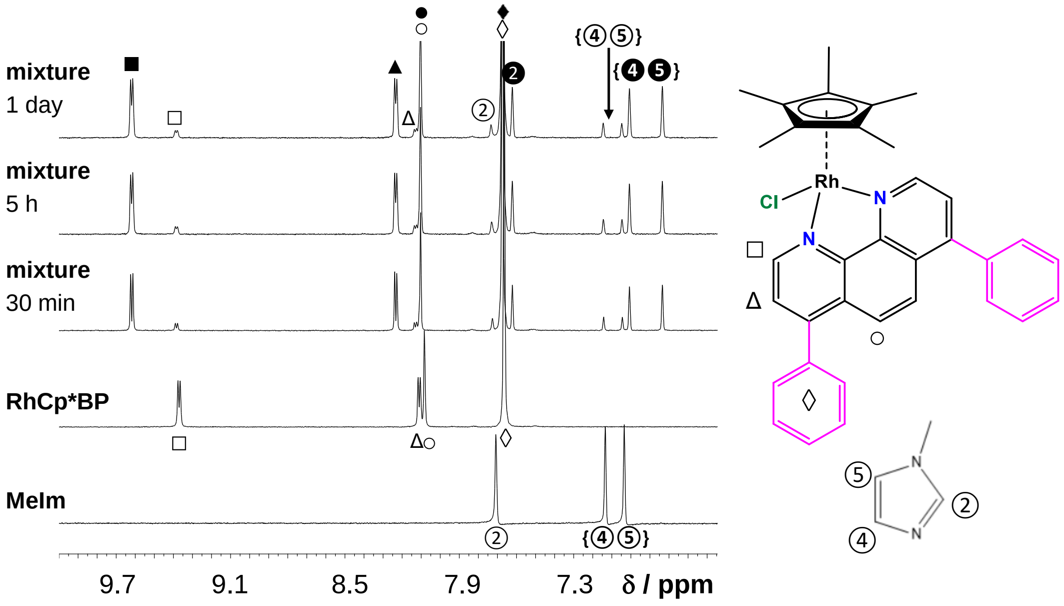

Figure S21. ^{1}H NMR spectra illustrating the interaction between RhCp*BP and MeIm at a 1:1 molar ratio, empty symbols indicate the metal complex and MeIm, filled symbols denote the RhCp*BP-MeIm complex. {$c_{complex/MeIm}$ = 0.5 mM; PBS'; t = 25 °C; 10% (v/v) D_2O / 90% (v/v) H_2O}.

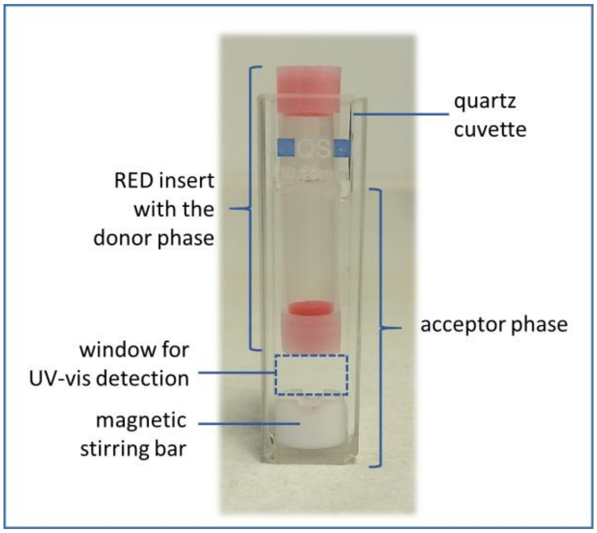

Figure S22. Modified Rapid Equilibrium Dialysis (RED) setup for release experiments in PBS' medium, RED insert is fitted into a 1 cm quartz cuvette for UV-Vis detection.

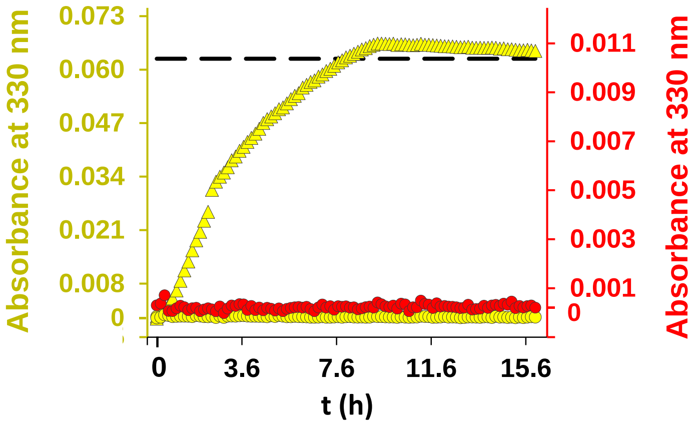

Figure S23. Absorbance at 330 nm over time for free RhCp*BP (▲), RhCp*BP time-depend release profile from HSA (●), and HTP formulation (●) in PBS' buffer (pH=7.4) medium over 16 h. The dashed line (- - -) represents the maximum achievable RhCp*BP release for the HSA and HTP formulations. The release was followed using a modified Rapid equilibrium dialysis setup (Thermo Scientific, Figure S19) adapted to on-line UV-Vis detection (see details in the Experimental section).

Table S5. Mean pIC_{50} values and standard deviation of the tested compounds against the breast cancer cell lines.

pIC_{50} (mean±SD)	MDA-MB-231			MCF-7			T47D		
PHEN	5.50	±	0.05	5.07	±	0.05	5.26	±	0.21
BP	6.12	±	0.10	6.23	±	0.00	6.20	±	0.03
[RhCp*(BP)Cl]Cl	5.33	±	0.01	4.83	±	0.11	5.28	±	0.05
[RuCym(BP)Cl]Cl	5.91	±	0.02	5.71	±	0.01	5.73	±	0.13
DCP	5.29	±	0.01	5.42	±	0.04	5.37	±	0.06
[RhCp*(DCP)Cl]Cl	5.12	±	0.08	5.23	±	0.01	5.13	±	0.07
[RuCym(DCP)Cl]Cl	<4.00			4.12	±	0.03	4.13	±	0.07
cisplatin	5.50	±	0.20	5.32	±	0.17	5.16	±	0.17

Table S6. Mean pIC_{50} values and standard deviation of the tested compounds against the uterine sarcoma cell lines.

pIC_{50} (mean±SD)	Mes-Sa mCherry			Mes-Sa/B1 mOrange			Mes-Sa/Dx5 eGFP		
**384-well plate**									
PHEN	5.53	±	0.04	5.86	±	0.07	6.38	±	0.08
BP	6.33	±	0.14	6.86	±	0.17	8.07	±	0.10
[RhCp*(BP)Cl]Cl	5.81	±	0.08	5.86	±	0.09	6.63	±	0.14
[RuCym(BP)Cl]Cl	6.28	±	0.05	4.26	±	0.11	4.39	±	0.02
DCP	5.52	±	0.02	5.31	±	0.06	6.31	±	0.07
[RhCp*(DCP)Cl]Cl	5.38	±	0.04	5.20	±	0.02	6.12	±	0.03
[RuCym(DCP)Cl]Cl	4.18	±	0.13	<4.00			<4.00		
cisplatin	5.83	±	0.16	5.77	±	0.10	5.37	±	0.16
etoposide	6.70	±	0.10	5.93	±	0.03	5.68	±	0.18
**96-well plate**									
[RhCp*(BP)Cl]Cl	5.54	±	0.07	5.61	±	0.10	5.75	±	0.03
with cross-linked HSA	5.41	±	0.04	5.26	±	0.09	5.59	±	0.03
with HTP	5.84	±	0.09	5.49	±	0.10	5.80	±	0.24

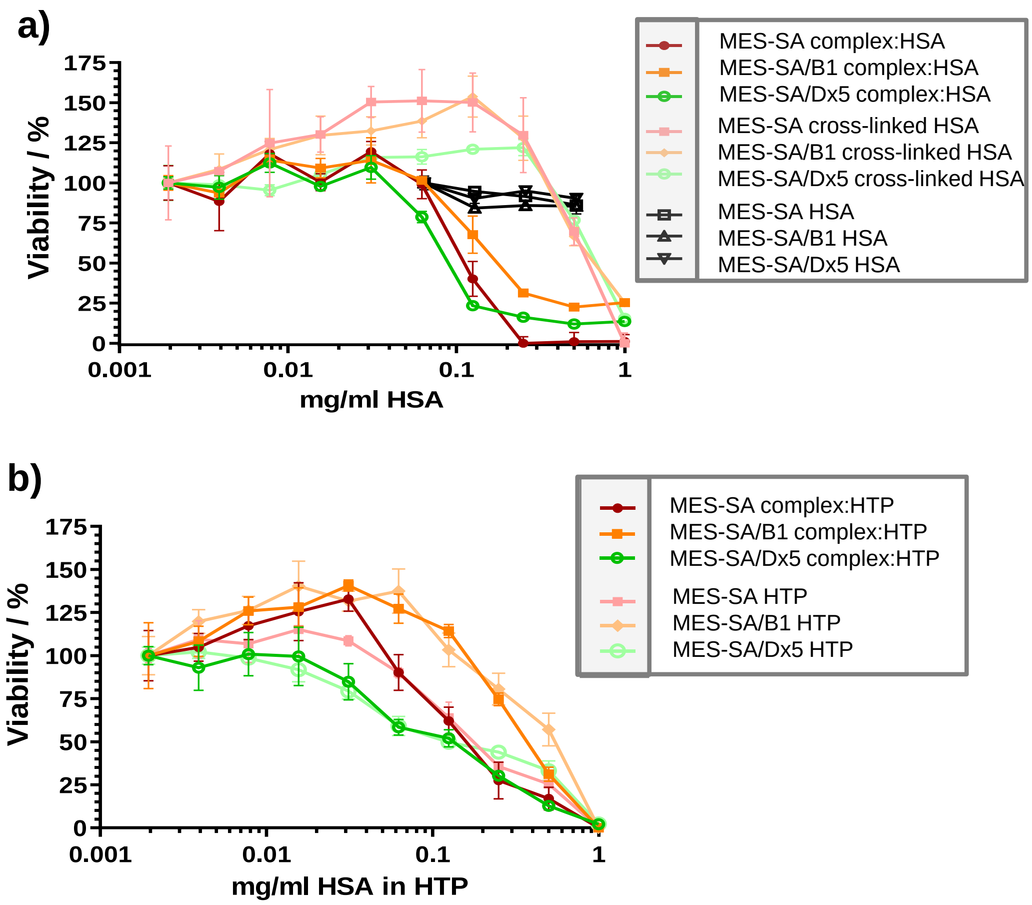

Figure S24. Representative results of dose-response relationship of a) HSA (alone, cross-linked HSA with and without complex [RhCp*(BP)Cl]Cl), and b) HTP (with or without the complex [RhCp*(BP)Cl]Cl) cytotoxicity against cocultured MES-SA mCh, MES-SA/B1 mOr and MES-SA/Dx5 eGFP cell lines. The cell viability above 100% occurred only in the HSA-based formulated samples. This effect is most likely attributed to the nutritional and growth-promoting properties of HSA itself.

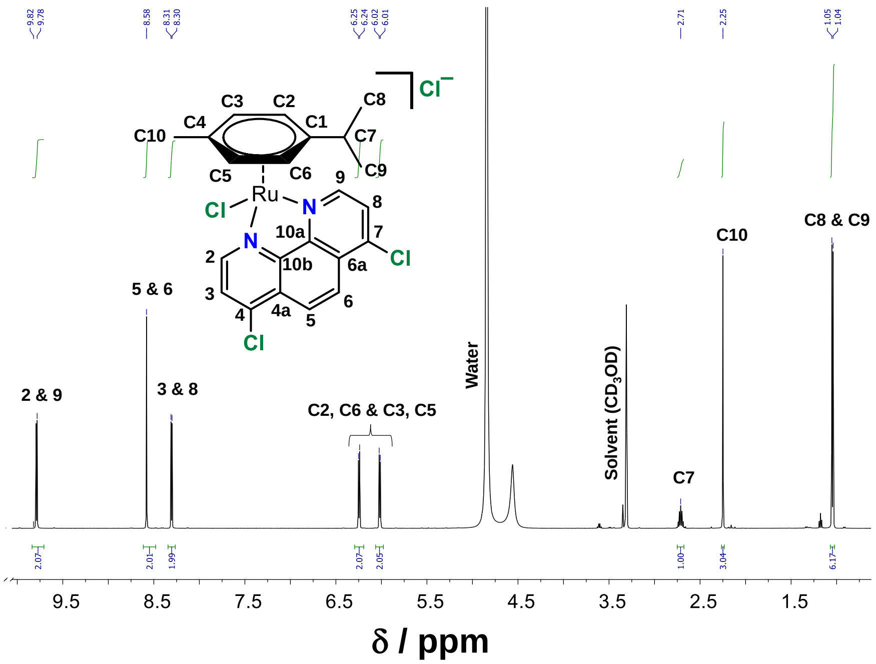

Figure S25. ^{1}H NMR spectrum of [RuCym(DCP)Cl]Cl in CD_3OD. Inserted structure shows numbering of peaks. {$c_{complex}$ = 10 mM, t = 25.0°C}

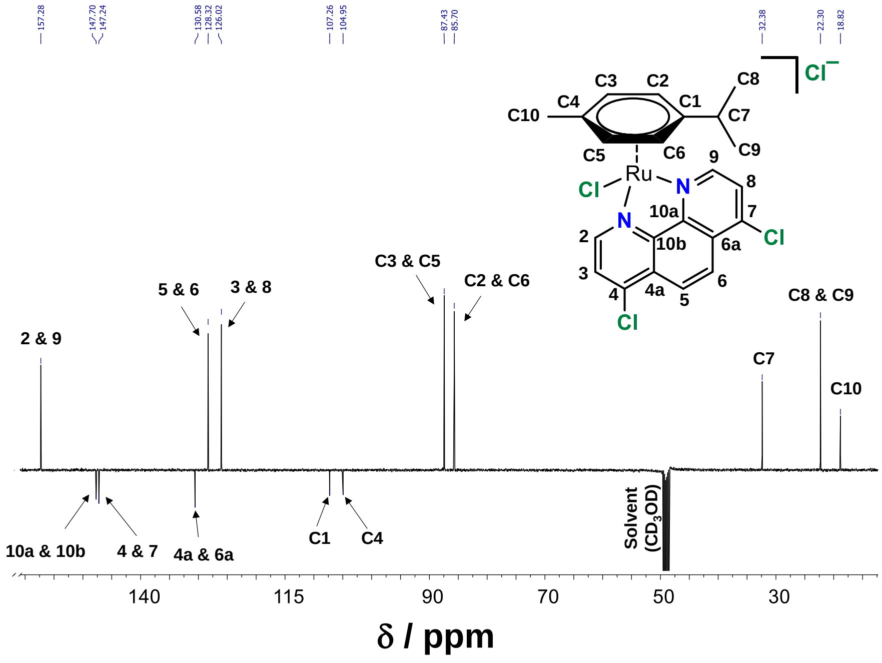

Figure S26. ^{13}C APT NMR spectrum of [RuCym(DCP)Cl]Cl in CD_3OD. Attached proton test method: CH and CH_3 peaks are positive, C and CH_2 peaks are negative. Inserted structure shows numbering of peaks. {$c_{complex}$ = 10 mM, t = 25.0°C}

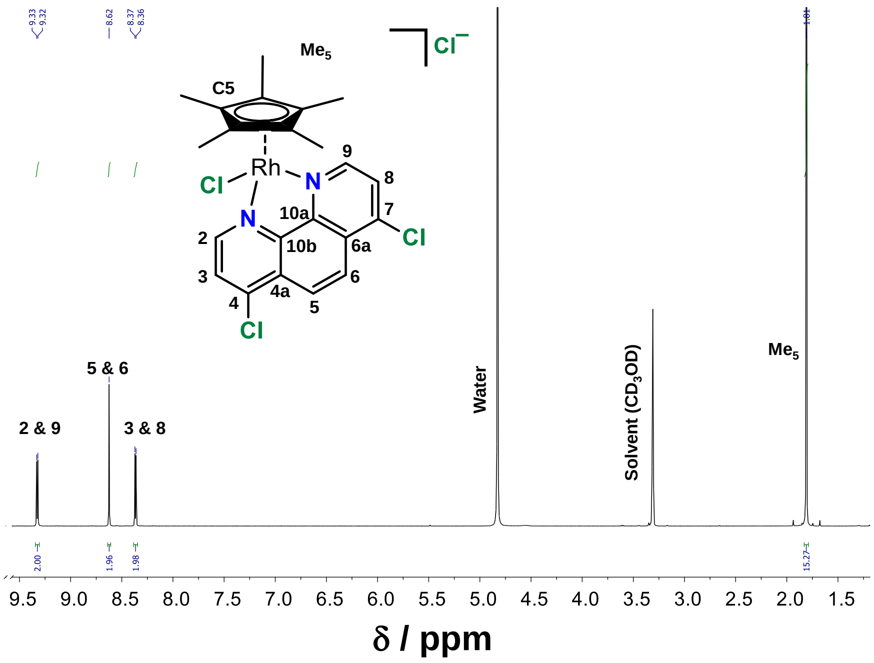

Figure S27. ^{1}H NMR spectrum of [RhCp*(DCP)Cl]Cl in CD_3OD. Inserted structure shows numbering of peaks. {$c_{complex}$ = 10 mM, t = 25.0°C}

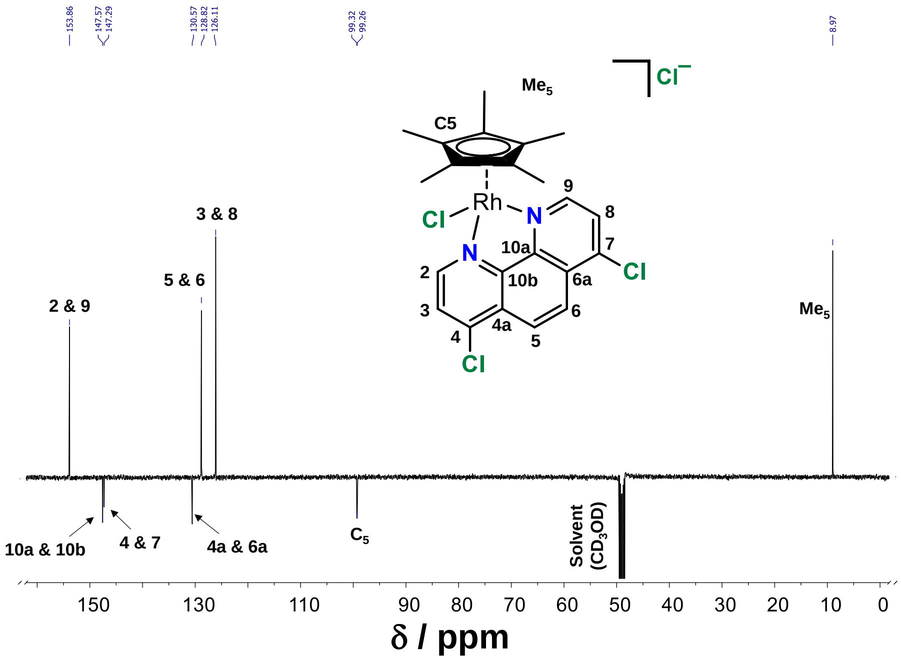

Figure S28. ^{13}C APT NMR spectrum of [RhCp*(DCP)Cl]Cl in CD_3OD. Attached proton test method: CH and CH_3 peaks are positive, C and CH_2 peaks are negative. Inserted structure shows numbering of peaks. {$c_{complex}$ = 10 mM, t = 25.0°C}

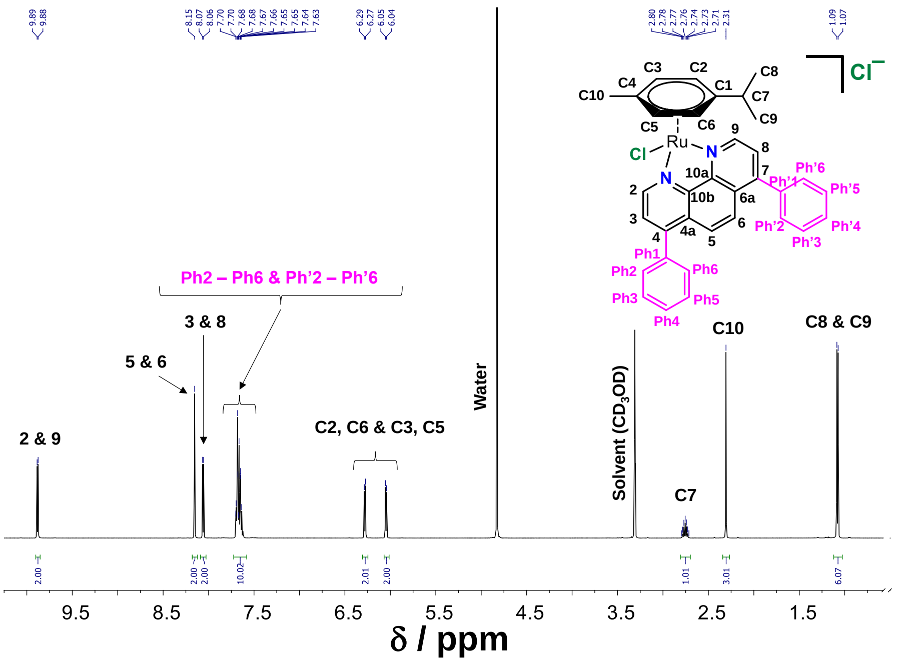

Figure S29. ^{1}H NMR spectrum of [RuCym(BP)Cl]Cl in CD_3OD. Inserted structure shows numbering of peaks. {$c_{complex}$ = 10 mM, t = 25.0°C}

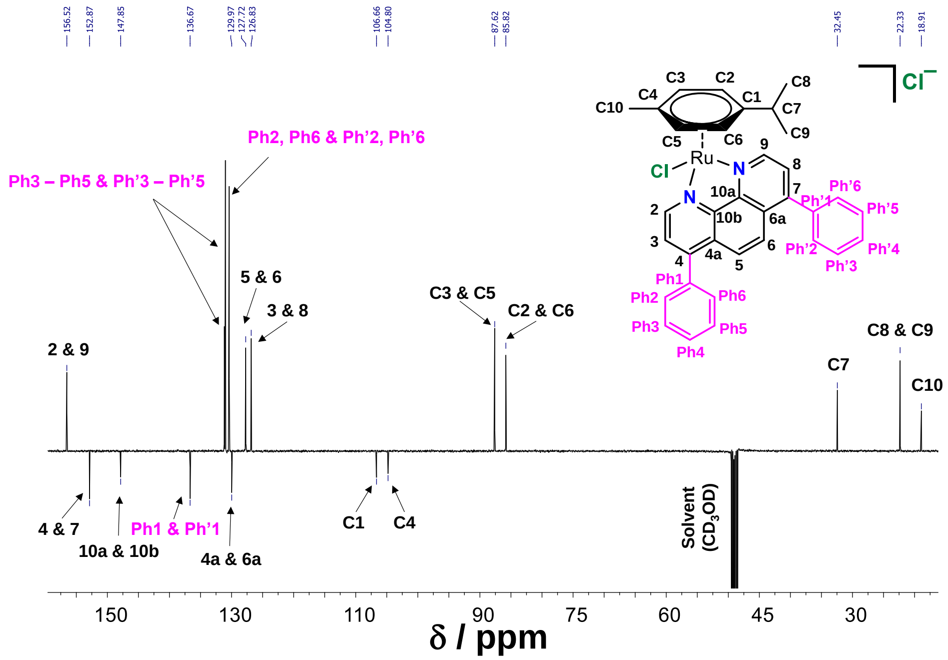

Figure S30. ^{13}C APT NMR spectrum of [RuCym(BP)Cl]Cl in CD_3OD. Attached proton test method: CH and CH_3 peaks are positive, C and CH_2 peaks are negative. Inserted structure shows numbering of peaks. {$c_{complex}$ = 10 mM, t = 25.0°C}

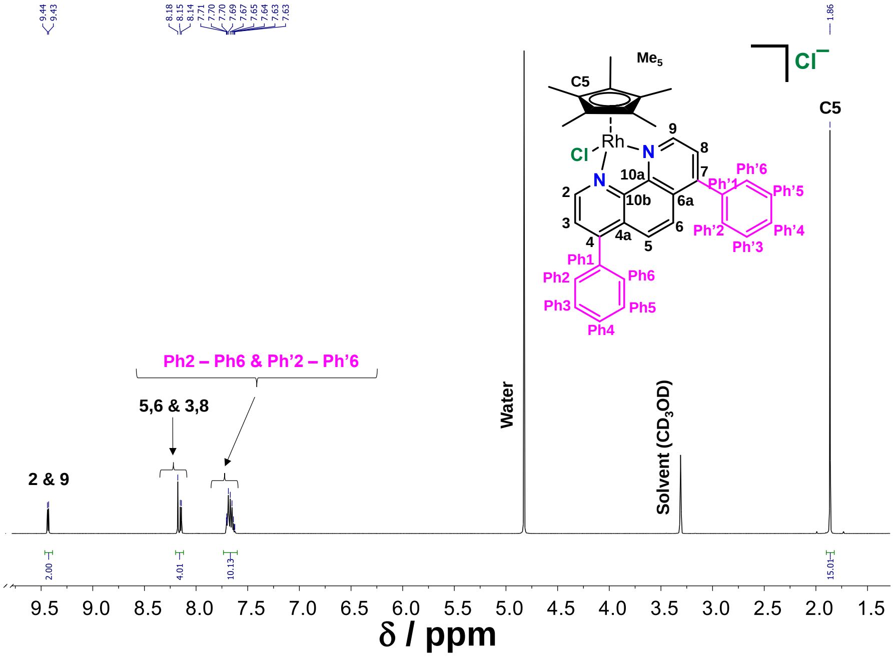

Figure S31. ^{1}H NMR spectrum of [RhCp*(BP)Cl]Cl in CD_3OD. Inserted structure shows numbering of peaks. {$c_{complex}$ = 10 mM, t = 25.0°C}

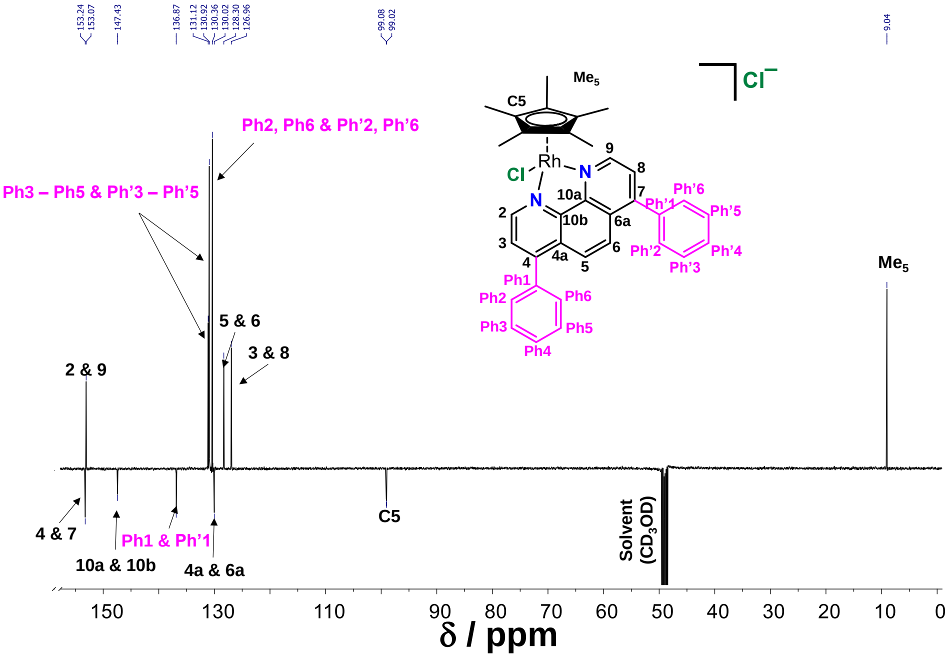

Figure S32. ^{13}C APT NMR spectrum of [RhCp*(BP)Cl]Cl in CD_3OD. Attached proton test method: CH and CH_3 peaks are positive, C and CH_2 peaks are negative. Inserted structure shows numbering of peaks. {$c_{complex}$ = 10 mM, t = 25.0°C}

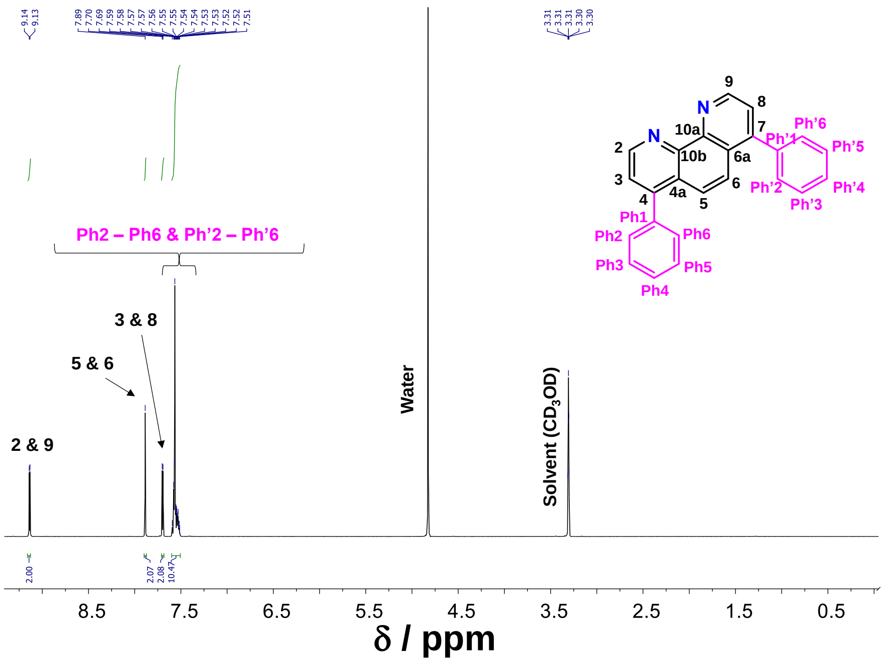

Figure S33. ^{1}H NMR spectrum of BP in CD_3OD. Inserted structure shows numbering of peaks. {c_{BP} = 10 mM, t = 25.0°C} δ 9.14 (d, J = 4.5 Hz, 1H), 7.89 (s, 1H), 7.70 (d, J = 4.5 Hz, 1H), 7.60 – 7.51 (m, 5H).

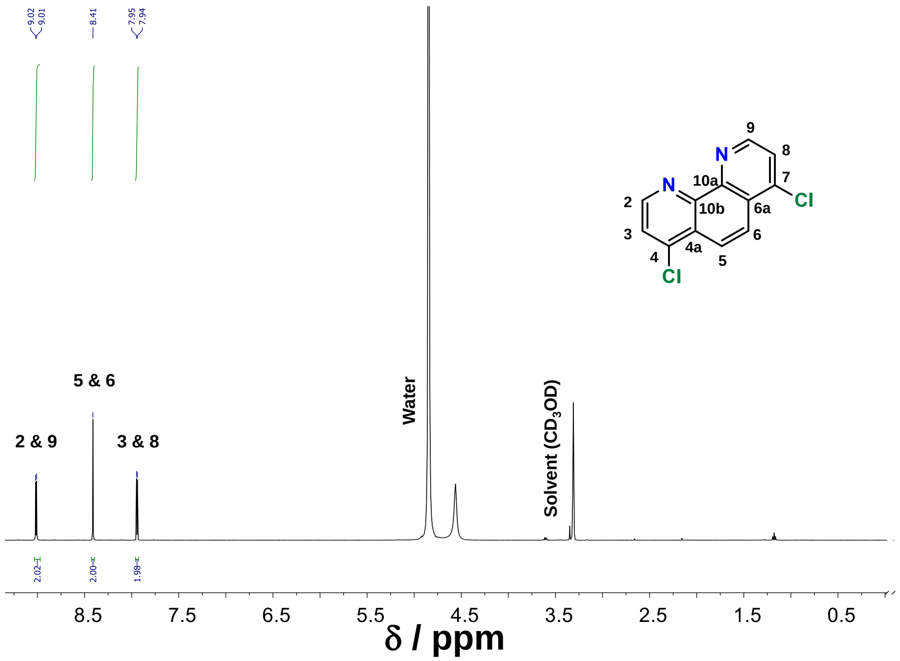

Figure S34. ^{1}H NMR spectrum of DCP in CD_3OD. Inserted structure shows numbering of peaks. {c_{DCP} = 10 mM, t = 25.0°C} δ 9.01 (d, J = 4.8 Hz, 1H), 8.41 (s, 1H), 7.94 (d, J = 4.8 Hz, 1H).

Table S7. Conditions applied for steady-state spectrofluorometric measurements.

	c_{HSA} (μM)	c_{MARKER} (μM)	$c_{complex}$ (μM)	λ_{EX} (nm)	λ_{EM} (nm)	slit widths EX/EM (nm/nm)
**Trp214 quenching**	1.2	1.2	0–25	295	300–500	3/3
**WF displacement**	1.2	1.2	0–25	310	320–500	3/3
**DG displacement**	1.3	1.2	0–25	335	420–600	4/4

References

(1) Colthup, N. B.; Daly, L. H.; Wiberley, S. E.; Colthup, N. B.; Daly, L. H.; Wiberley, S. E. Chapter 7 – Olefin Groups. *Introduction to Infrared and Raman Spectroscopy* **1990**, *1*, 247–260.

(2) Bencze, É.; Lokshin, B. V.; Mink, J.; Herrmann, W. A.; Kühn, F. E. Vibrational Spectra and Structure of the Cyclopentadienyl-Anion (Cp−), the Pentamethylcyclopentadienyl-Anion (Cp*−) and of Alkali Metal Cyclopentadienyls CpM and Cp*M (M=Li, Na, K). *J Organomet Chem* **2001**, *627* (1), 55–66. https://doi.org/10.1016/S0022-328X(01)00710-0.

(3) Amberger, H. D.; Reddmann, H. Electronic Structures of Organometallic Complexes of f Elements LXXIV: First Raman Spectroscopic Polarization Measurements on Uniformly Oriented Sandwich Complex Molecules: Bis(H5-Pentamethylcyclopentadienyl)Ruthenium. *J Organomet Chem* **2010**, *695* (22), 2455–2460. https://doi.org/10.1016/J.JORGANCHEM.2010.07.007.

(4) Hagebaum-Reignier, D.; Girardi, R.; Carissan, Y.; Humbel, S. Hückel Theory for Lewis Structures: Hückel-Lewis Configuration Interaction (HL-CI). *Journal of Molecular Structure: THEOCHEM* **2007**, *817* (1–3), 99–109. https://doi.org/10.1016/j.theochem.2007.04.026.

(5) Carissan, Y.; Hagebaum-Reignier, D.; Goudard, N.; Humbel, S. Hückel-Lewis Projection Method: A "Weights Watcher" for Mesomeric Structures. *Journal of Physical Chemistry A* **2008**, *112* (50), 13256–13262. https://doi.org/10.1021/jp803813e.

(6) Nakamoto, K. Complexes of Alcohols, Ethers, Ketones, Aldehydes, Esters and Carboxylic Acids. Complexes of Amino Acids. *Infrared and Raman Spectra of Inorganic and Coordination Compounds, Applications in Coordination, Organometallic, and Bioinorganic Chemistry* **2009**, 231–233.

(7) Colina-Vegas, L.; Villarreal, W.; Navarro, M.; De Oliveira, C. R.; Graminha, A. E.; Maia, P. I. D. S.; Deflon, V. M.; Ferreira, A. G.; Cominetti, M. R.; Batista, A. A. Cytotoxicity of Ru(II) Piano-Stool Complexes with Chloroquine and Chelating Ligands against Breast and Lung Tumor Cells: Interactions with DNA and BSA. *J Inorg Biochem* **2015**, *153*, 150–161. https://doi.org/10.1016/j.jinorgbio.2015.07.016.

(8) Savić, A.; Gligorijević, N.; Aranđelović, S.; Dojčinović, B.; Kaczmarek, A. M.; Radulović, S.; Van Deun, R.; Van Hecke, K. Antitumor Activity of Organoruthenium Complexes with Chelate

Aromatic Ligands, Derived from 1,10-Phenantroline: Synthesis and Biological Activity. *J Inorg Biochem* **2020**, *202*, 110869. https://doi.org/10.1016/j.jinorgbio.2019.110869.

(9) Youinou, M. T.; Ziessel, R. Synthesis and Molecular Structure of a New Family of Iridium-(III) and Rhodium(III) Complexes: [(H5-Me5C5)Ir(LL)X]+ and [(H5-Me5C5)Rh(LL)Cl]+; LL = 2,2′-Bipyridine or 1,10-Phenanthroline; X = Cl or H. Single Crystal Structures of [(H5-Me5C5)Ir(Bpy)Cl]Cl and [(H5-Me5C5)Rh(Phen)Cl]ClO4. *J Organomet Chem* **1989**, *363* (1–2), 197–208. https://doi.org/10.1016/0022-328X(89)88054-4.

(10) Scharwitz, M. A.; Ott, I.; Geldmacher, Y.; Gust, R.; Sheldrick, W. S. Cytotoxic Half-Sandwich Rhodium(III) Complexes: Polypyridyl Ligand Influence on Their DNA Binding Properties and Cellular Uptake. *J Organomet Chem* **2008**, *693* (13), 2299–2309. https://doi.org/10.1016/J.JORGANCHEM.2008.04.002.

(11) Blakemore, J. D.; Hernandez, E. S.; Sattler, W.; Hunter, B. M.; Henling, L. M.; Brunschwig, B. S.; Gray, H. B. Pentamethylcyclopentadienyl Rhodium Complexes. *Polyhedron* **2014**, *84*, 14–18. https://doi.org/10.1016/J.POLY.2014.05.022.

(12) Graf, M.; Ochs, J.; Metzler-Nolte, N.; Böttcher, H. C.; Mayer, P. Cytotoxic Activities of Half-Sandwich M(III) Complexes (M=Rh, Ir) Bearing Chloro-Substituted Bidentate-Coordinated Phenanthroline or Terpyridine Ligands. *Z Anorg Allg Chem* **2023**, *649* (15), e202300082. https://doi.org/10.1002/ZAAC.202300082;REQUESTEDJOURNAL:JOURNAL:15213749;PAGE:STRING:ARTICLE/CHAPTER.

(13) Scharwitz, M.; Schäfer, S.; Van Almsick, T.; Sheldrick, W. S. Chlorido(5-Penta-Methyl-Cyclo-Penta-Dien-Yl)(1,10- Phenanthroline-2 N,N′)Iridium(III) Trifluoro-Methane-Sulfonate. *Acta Crystallogr Sect E Struct Rep Online* **2007**, *63* (4), m1111–m1113. https://doi.org/10.1107/S1600536807011750/BT2291ISUP2.HKL.

(14) Ariyoshi, K.; Kotera, M.; Namioka, A.; Suzuki, T. A Specific Formation of an Iridium(III) Hydrido Complex Bearing 8-(Diphenylphosphino)Quinoline. *Polyhedron* **2020**, *179*, 114401. https://doi.org/10.1016/J.POLY.2020.114401.

(15) Gadre, S.; Manikandan, M.; Chakraborty, G.; Rayrikar, A.; Paul, S.; Patra, C.; Patra, M. Development of a Highly In Vivo Efficacious Dual Antitumor and Antiangiogenic Organoiridium Complex as a Potential Anti-Lung Cancer Agent. *J Med Chem* **2023**, *66* (19), 13481–13500. https://doi.org/10.1021/ACS.JMEDCHEM.3C00704/SUPPL_FILE/JM3C00704_SI_002.PDF.

(16) Peacock, A. F. A.; Habtemariam, A.; Moggach, S. A.; Prescimone, A.; Parsons, S.; Sadler, P. J. Chloro Half-Sandwich Osmium(II) Complexes: Influence of Chelated N,N-Ligands on Hydrolysis, Guanine Binding, and Cytotoxicity. *Inorg Chem* **2007**, *46* (10), 4049–4059. https://doi.org/10.1021/IC062350D.
